# Supplementary material for: The Clinical Kinase Index: A Method to Prioritize Understudied Kinases as Drug Targets for the Treatment of Cancer
Source: Cell Rep Med. 2020 Oct 20;1(7):100128. doi: 10.1016/j.xcrm.2020.100128 (PMC7659504; doi:10.1016/j.xcrm.2020.100128)
Supplement: Document S1. Figure S1 [file mmc1.pdf]

Cell Reports Medicine, Volume *1*

Supplemental Information

The Clinical Kinase Index: A Method to Prioritize Understudied Kinases as Drug Targets for the Treatment of Cancer

Derek Essegian, Rimpi Khurana, Vasileios Stathias, and Stephan C. Schürer

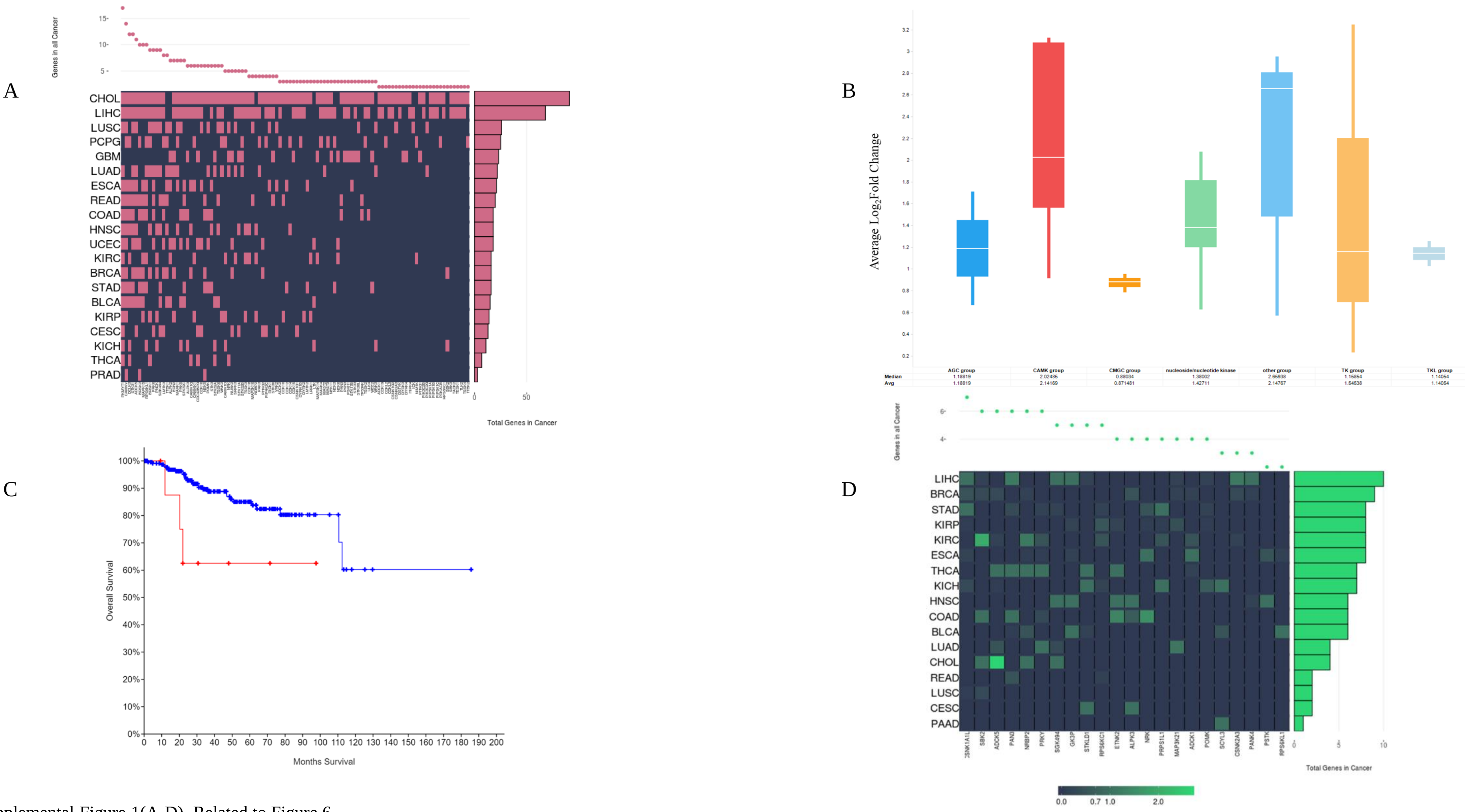

Supplemental Figure 1(A-D). Related to Figure 6.

A: Differential overexpression of Understudied kinases in each cancer cohort. 102 Understudied are significantly overexpressed in at-least two cancers.

B: Several kinase groups appear to be enriched in their overexpression in various cancers. Averaging the log2FC of all kinases across all cancers demonstrates that certain kinase transcripts are consistently very highly upregulated. Kinases from the understudied CAMK group have the highest average log2FC (2.14) and kinases from the Other group have the highest median log2FC (2.66).

C: Dark Kinase DYRK1B was mutated in 4.3% of Endometrial Carcinoma samples (UCEC) with significantly worse overall survival for patients harboring the mutated kinase ($p<.05$).

D: *Tdark* Kinases are potential prognostic biomarkers for several cancers. TNM staging scores (0-3) show that dark kinases contribute to tumor progression.

Supplemental Data 1 (A-P) Analysis of Clinically Relevant Understudied Kinases. Related to Figure 5: CKI scores of targets who have been under or are currently under clinical trial investigation versus those that have no been clinically evaluated in humans. Significant differences in distributions are signified by p-values <.05.

1A.

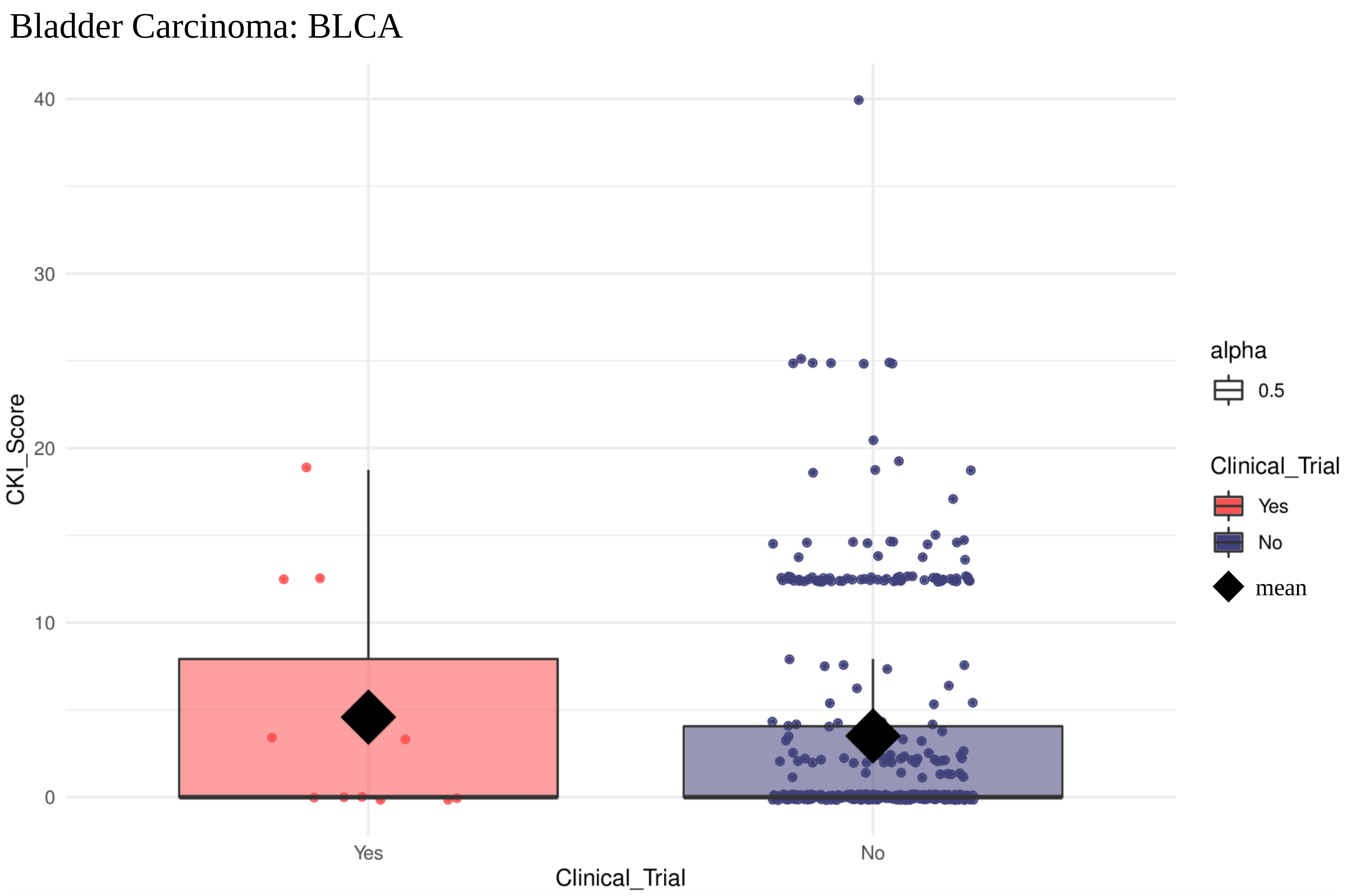
Bladder Carcinoma: BLCA
CKI_Score
40
30
20
10
0
Yes
No
Clinical_Trial
alpha
0.5
Clinical_Trial
Yes
No
mean

1B.

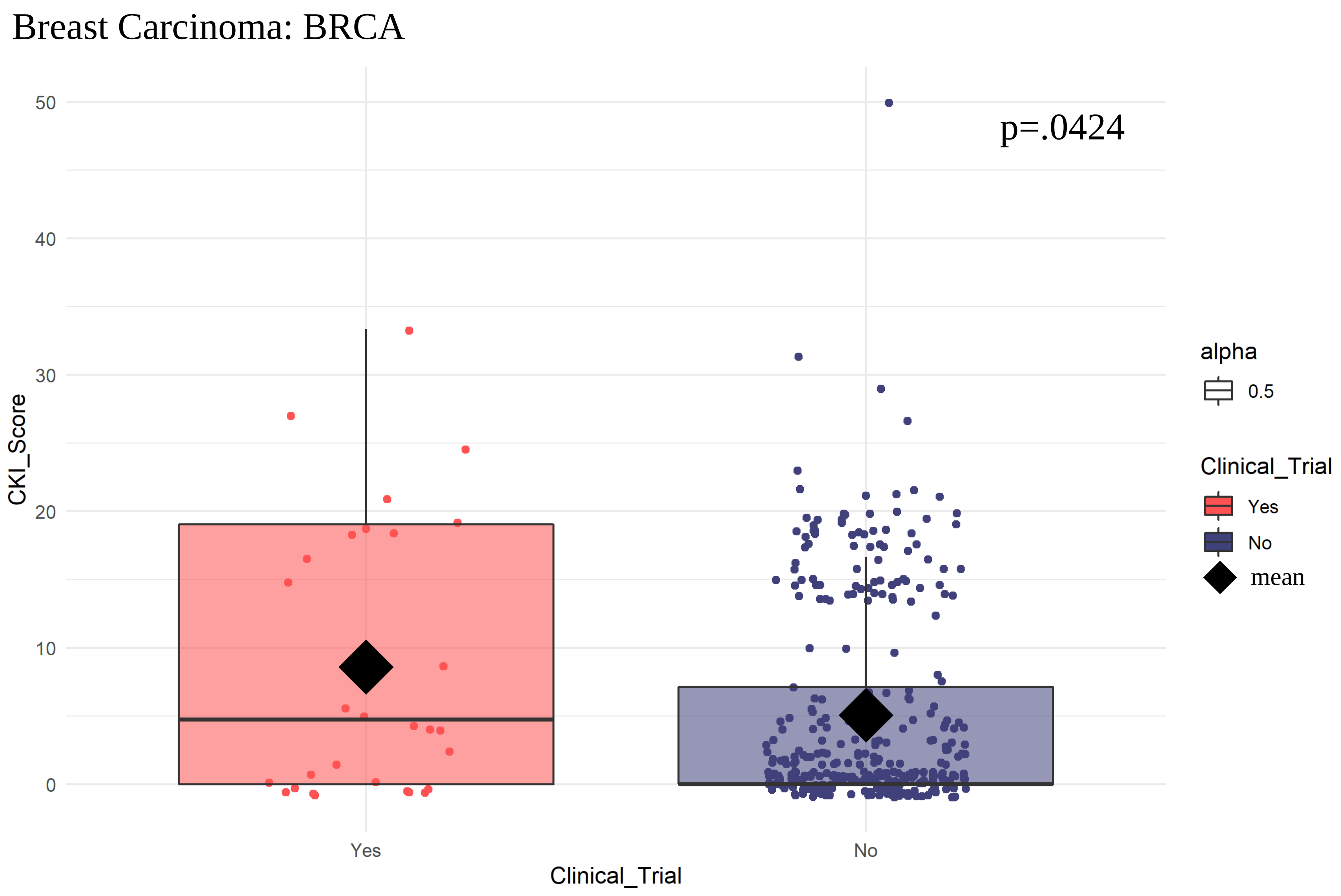
Breast Carcinoma: BRCA
p=.0424
50
40
30
20
10
0
CKI_Score
Yes
No
Clinical_Trial
alpha
0.5
Clinical_Trial
Yes
No
mean

1C.

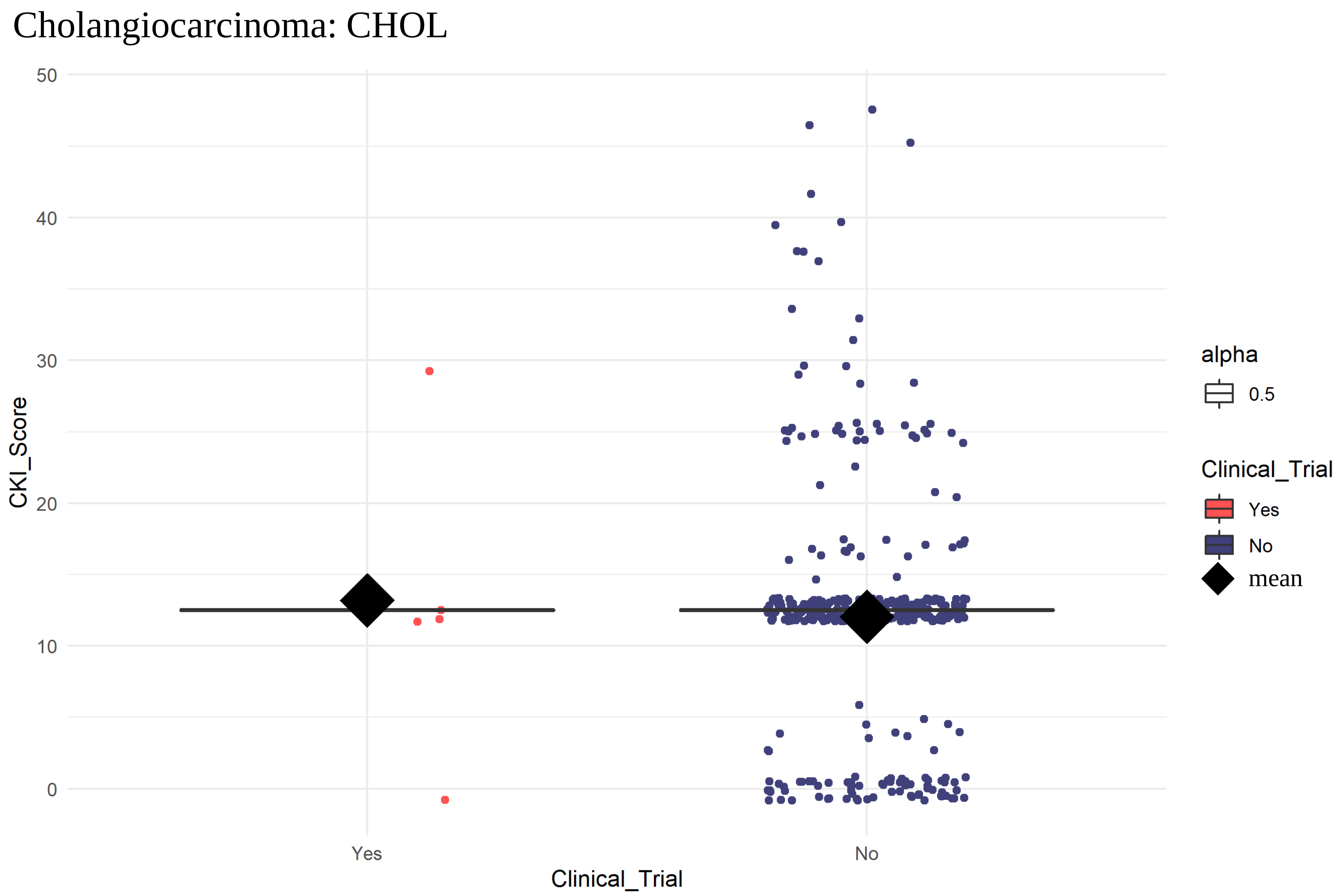

Cholangiocarcinoma: CHOL
50
40
30
20
10
0
CKI_Score
Yes
No
Clinical_Trial
alpha
0.5
Clinical_Trial
Yes
No
mean

1D.

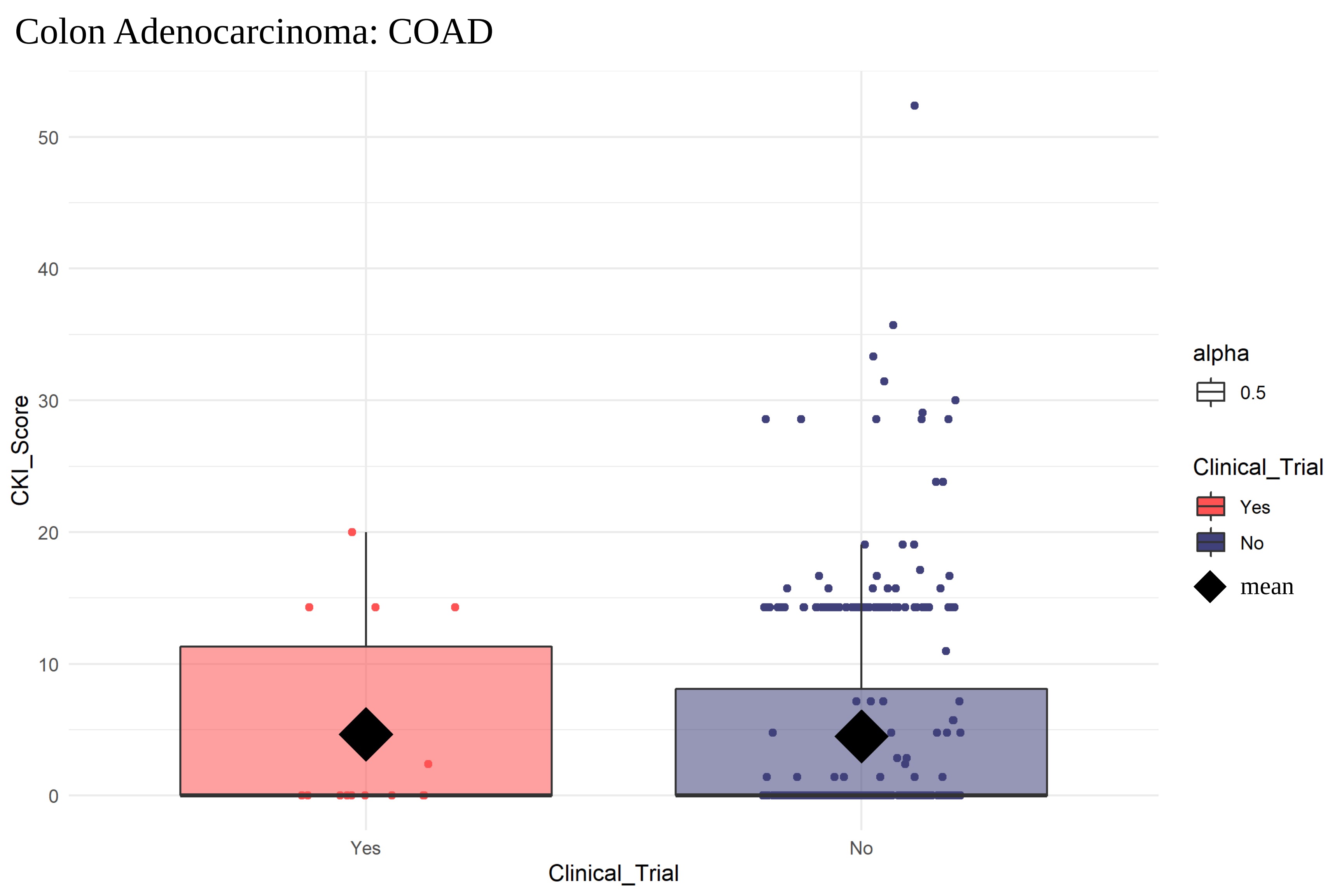
Colon Adenocarcinoma: COAD
CKI_Score
0
10
20
30
40
50
Yes
No
Clinical_Trial
alpha
0.5
Clinical_Trial
Yes
No
mean

1E.

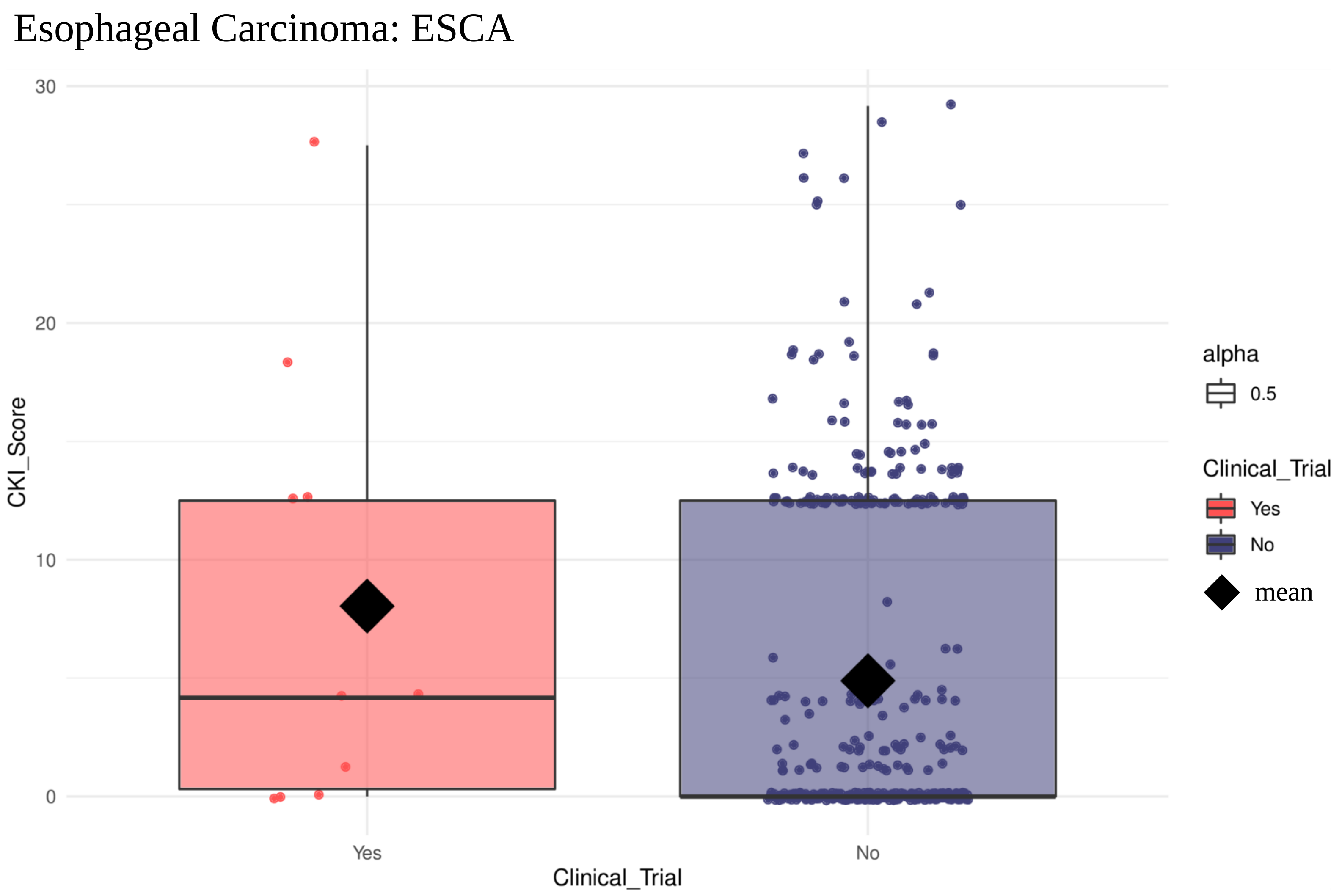
Esophageal Carcinoma: ESCA
30
20
10
0
CKI_Score
Yes
No
Clinical_Trial
alpha
0.5
Clinical_Trial
Yes
No
mean

1F.

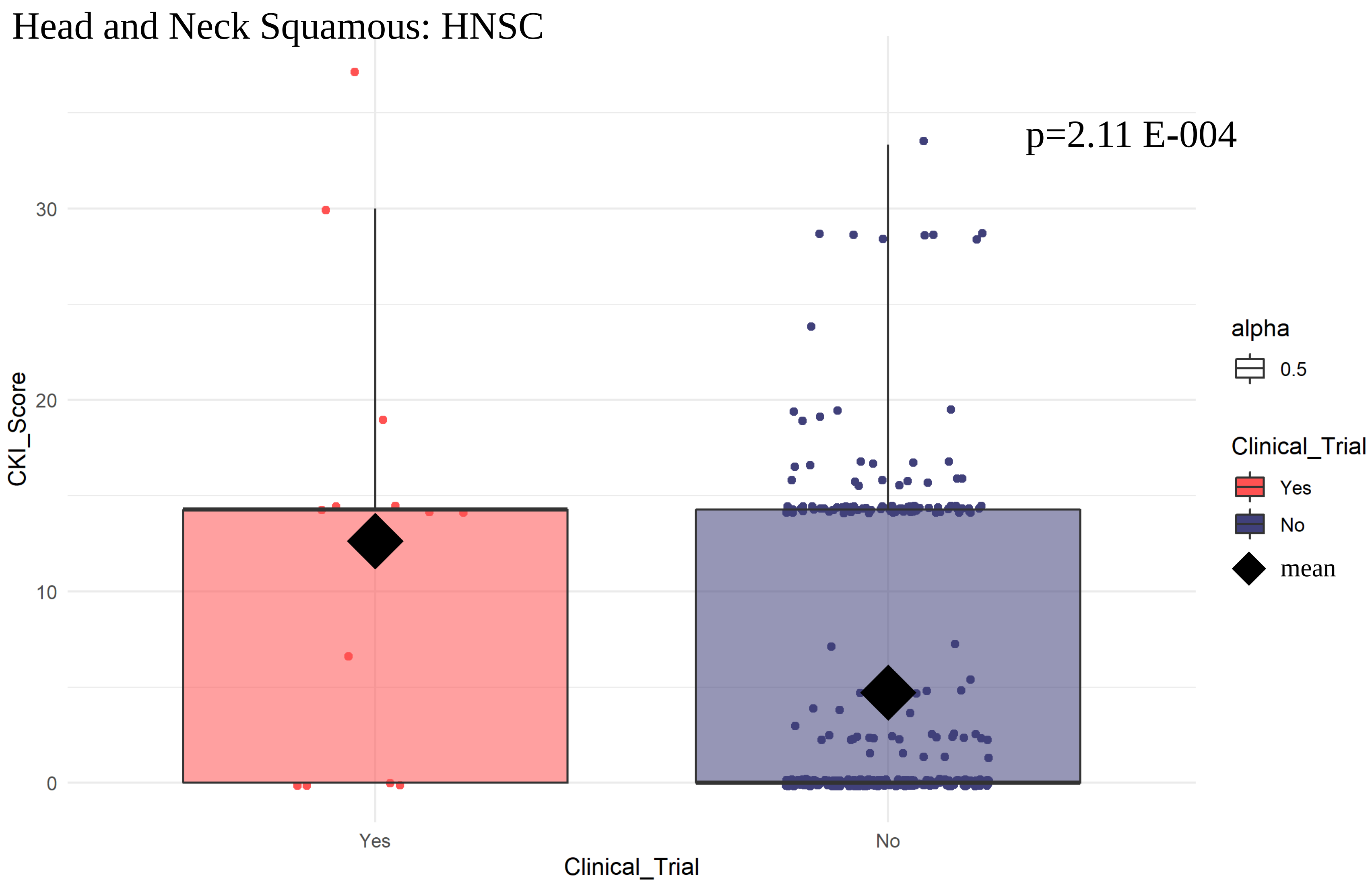
Head and Neck Squamous: HNSC
p=2.11 E-004
CKI_Score
30
20
10
0
Yes
No
Clinical_Trial
alpha
0.5
Clinical_Trial
Yes
No
mean

1G.

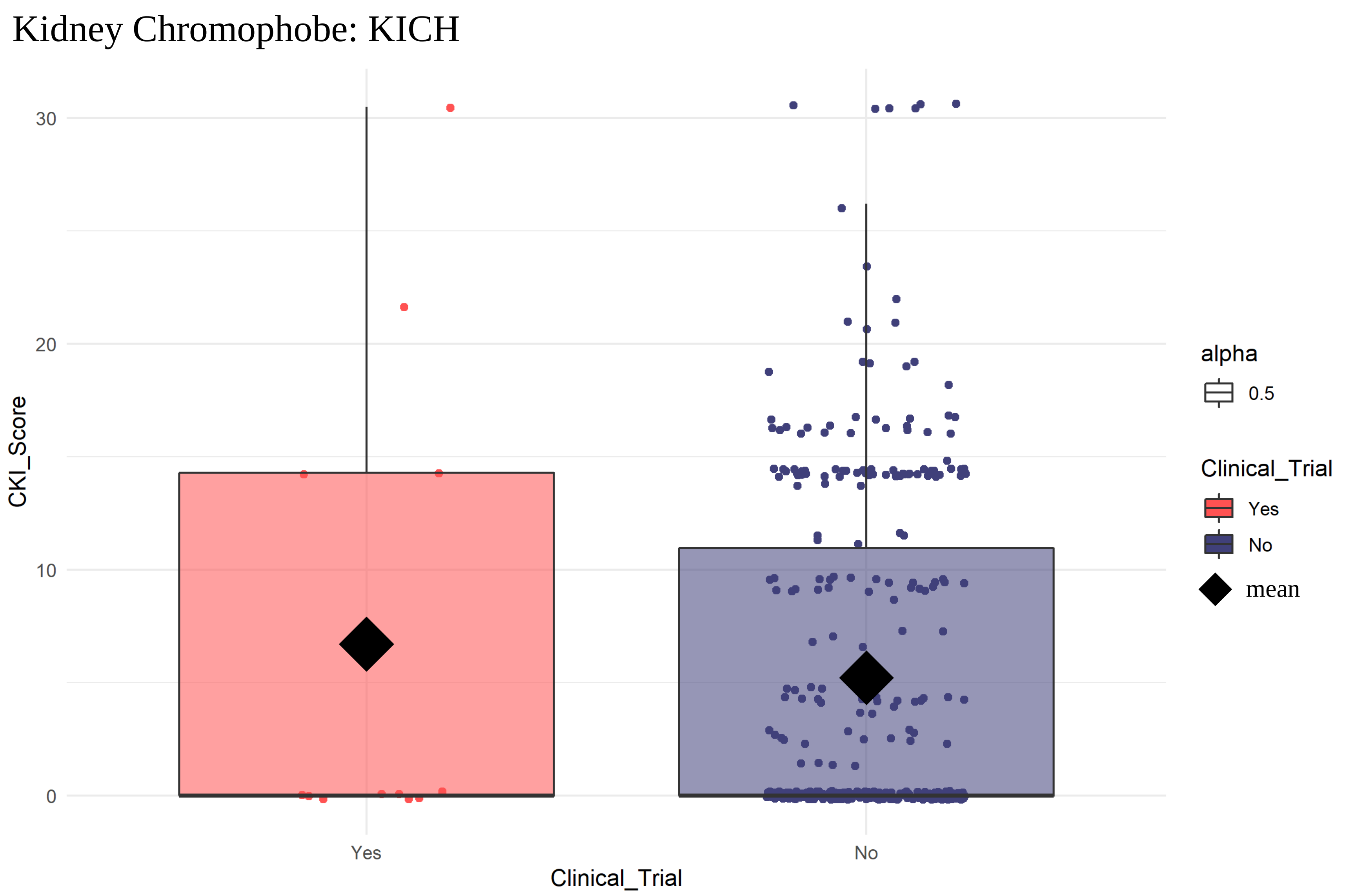

Kidney Chromophobe: KICH
30
20
10
0
CKI_Score
Yes
No
Clinical_Trial
alpha
0.5
Clinical_Trial
Yes
No
mean

1H.

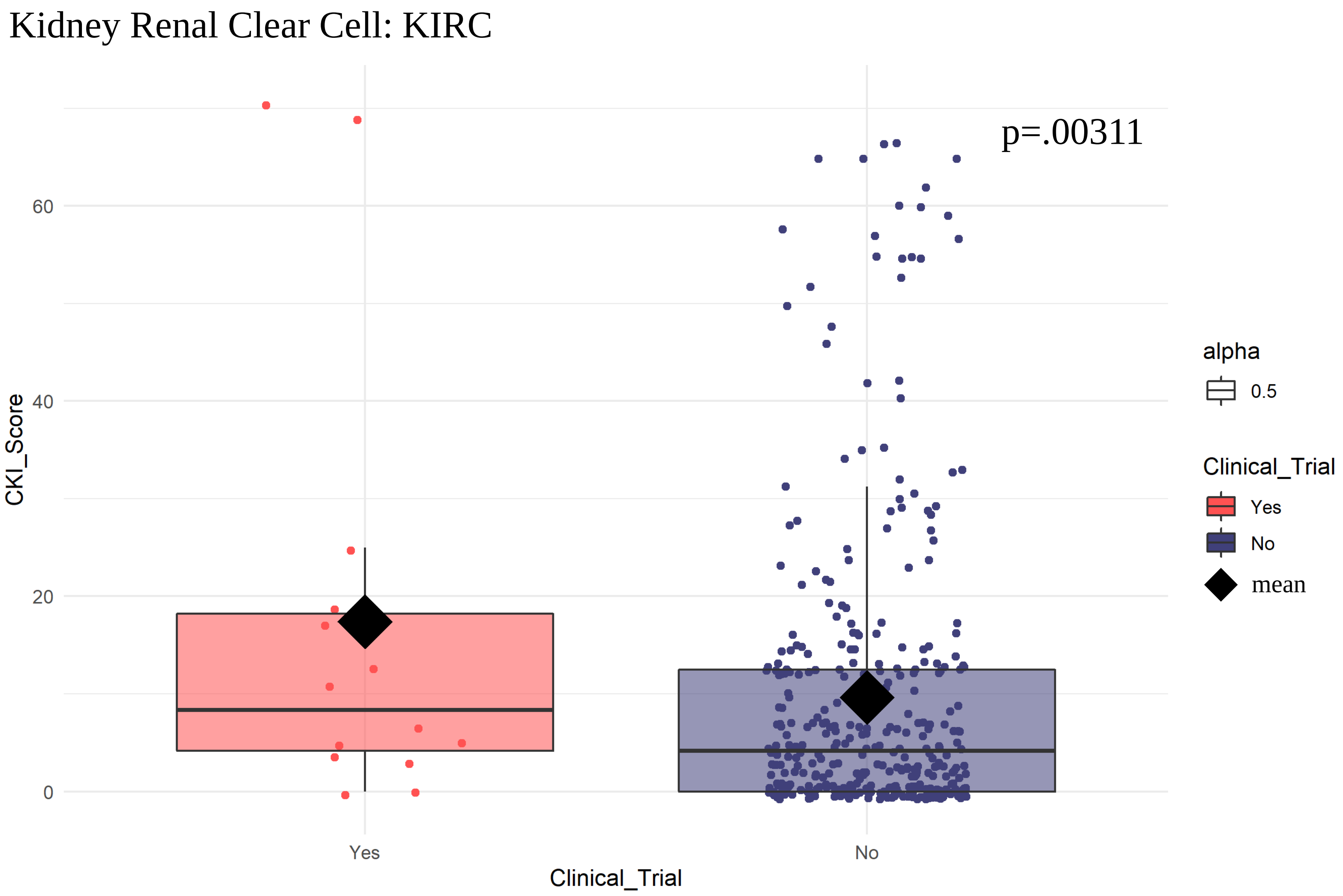
Kidney Renal Clear Cell: KIRC
p=.00311
CKI_Score
60
40
20
0
Yes
No
Clinical_Trial
alpha
0.5
Clinical_Trial
Yes
No
mean

1I.

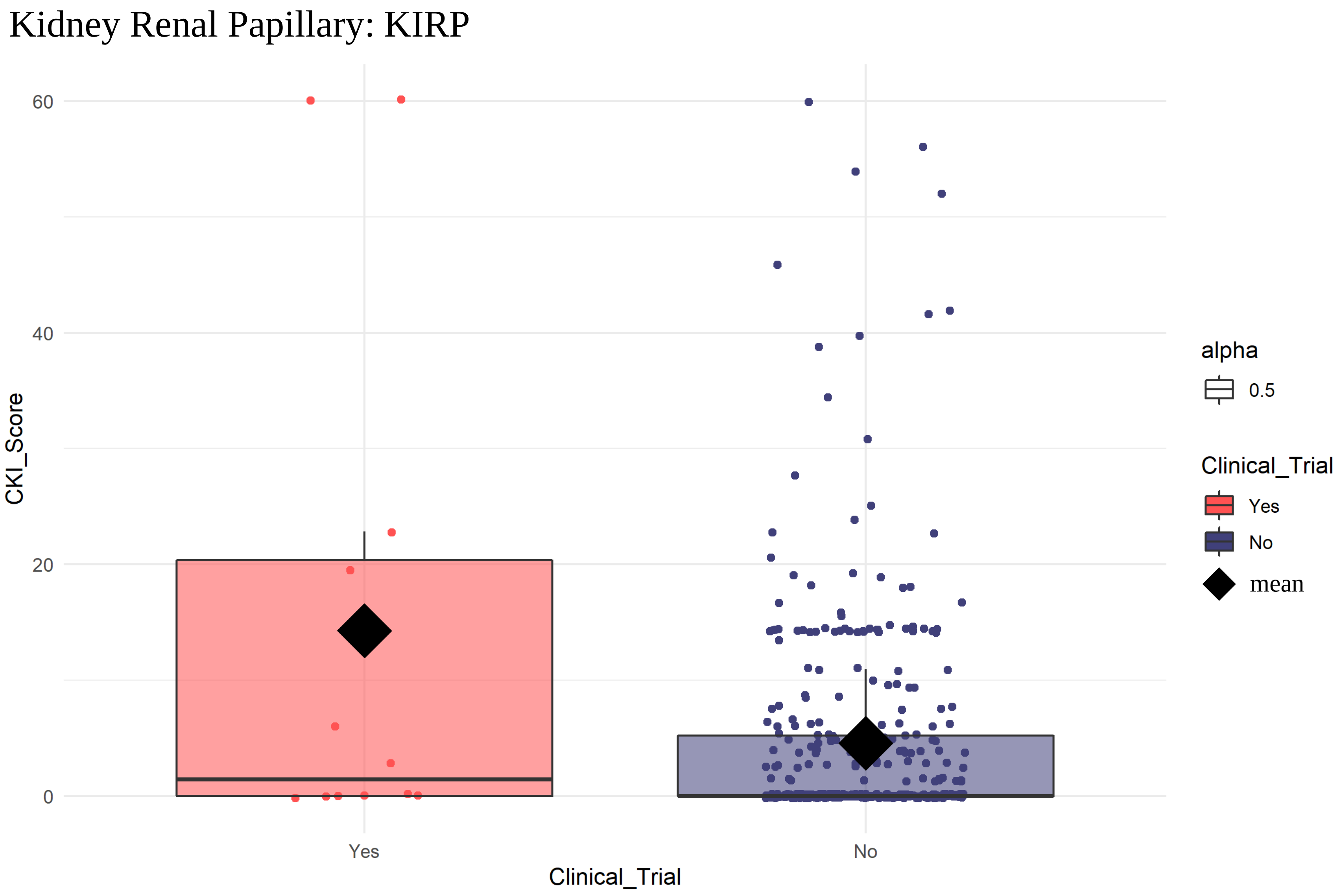

Kidney Renal Papillary: KIRP
60
40
20
0
CKI_Score
Yes
No
Clinical_Trial
alpha
0.5
Clinical_Trial
Yes
No
mean

1J.

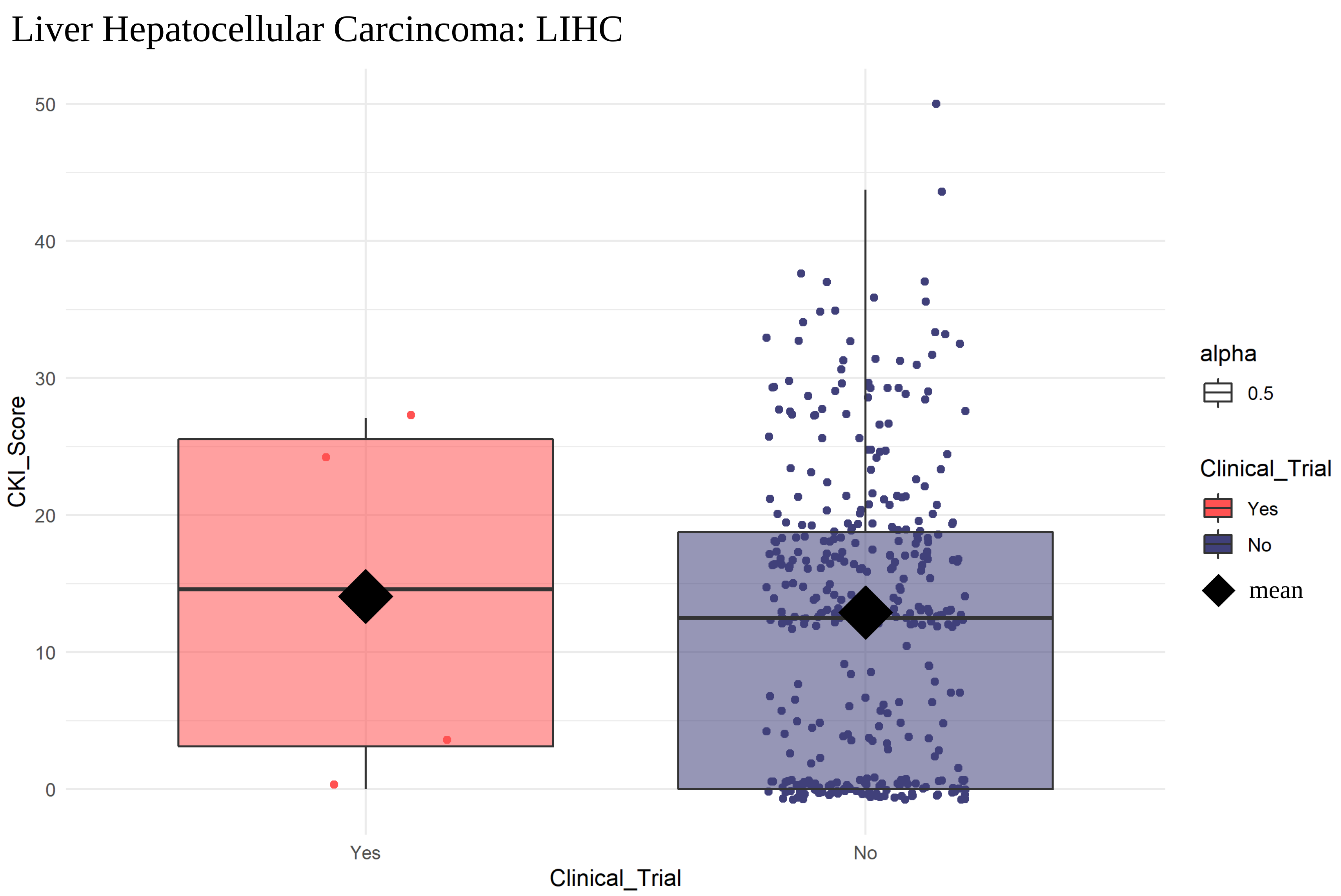
Liver Hepatocellular Carcincoma: LIHC
CKI_Score
50
40
30
20
10
0
Yes
No
Clinical_Trial
alpha
0.5
Clinical_Trial
Yes
No
mean

1K.

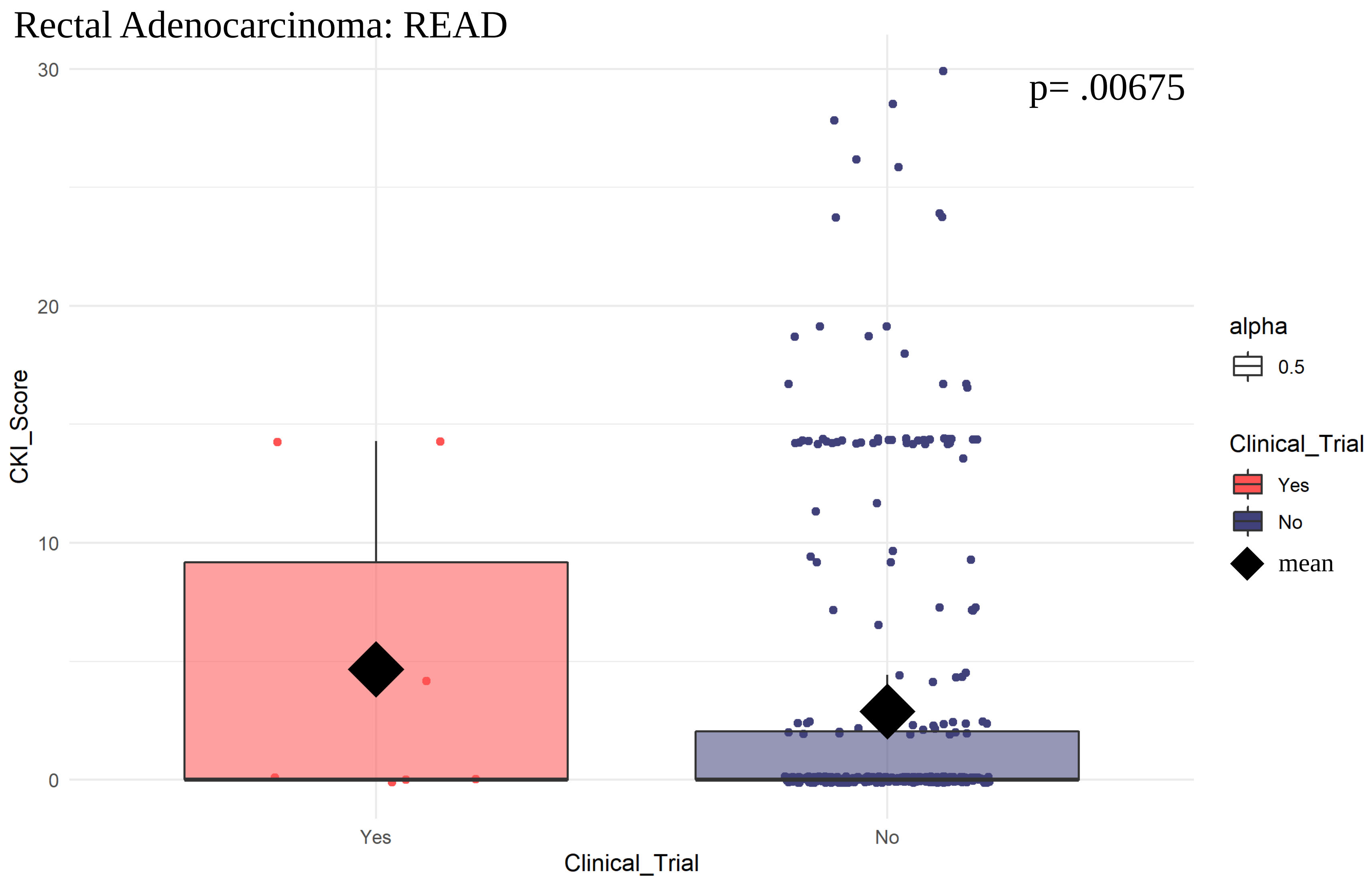
Rectal Adenocarcinoma: READ
p= .00675
30
20
10
0
CKI_Score
Yes
No
Clinical_Trial
alpha
0.5
Clinical_Trial
Yes
No
mean

1L.

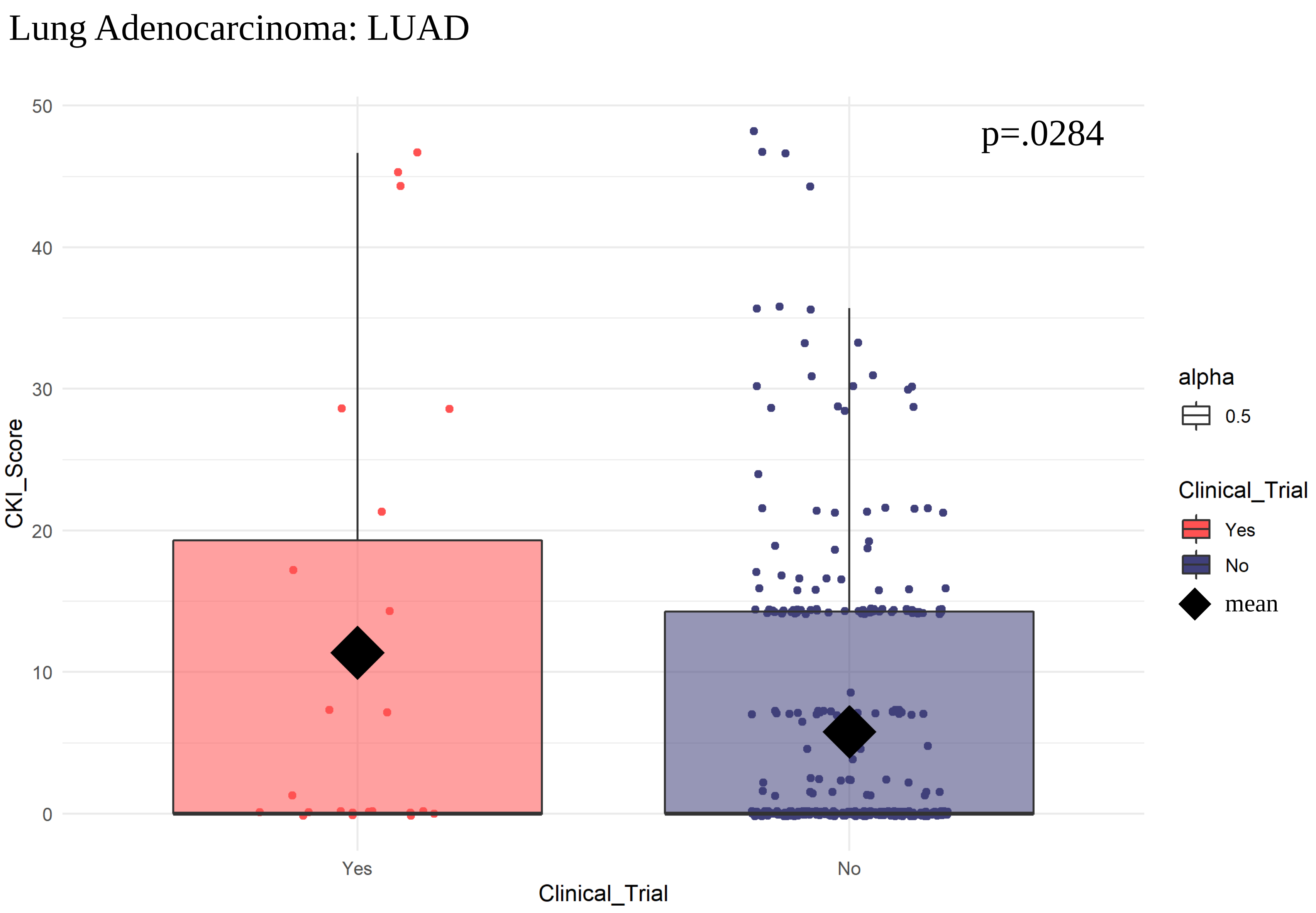

Lung Adenocarcinoma: LUAD
p=.0284
CKI_Score
50
40
30
20
10
0
Yes
No
Clinical_Trial
alpha
0.5
Clinical_Trial
Yes
No
mean

1M.

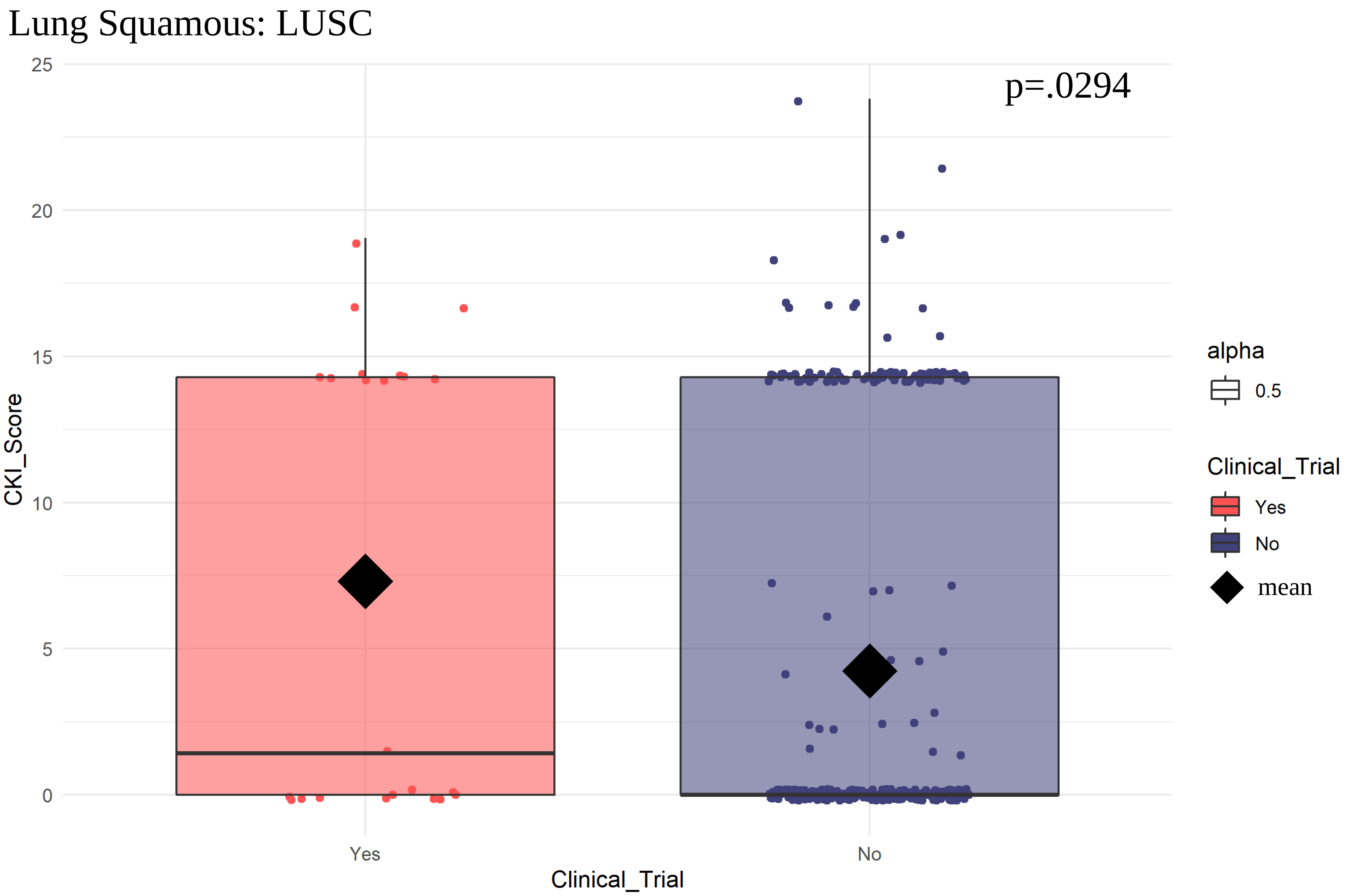
Lung Squamous: LUSC
p=.0294
25
20
15
10
5
0
CKI_Score
Yes
No
Clinical_Trial
alpha
0.5
Clinical_Trial
Yes
No
mean

1N.

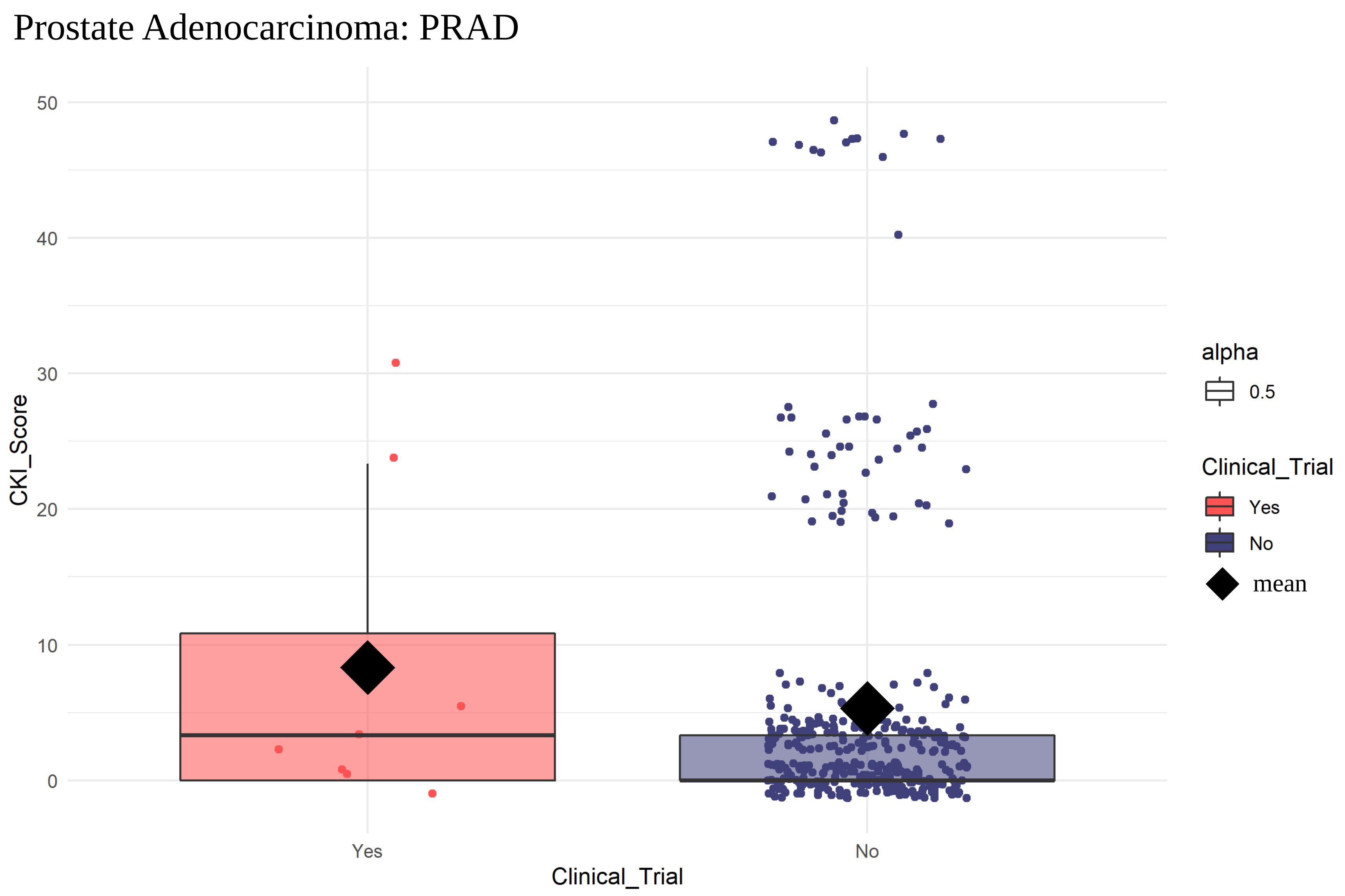
Prostate Adenocarcinoma: PRAD
50
40
30
20
10
0
CKI_Score
Yes
No
Clinical_Trial
alpha
0.5
Clinical_Trial
Yes
No
mean

1O.

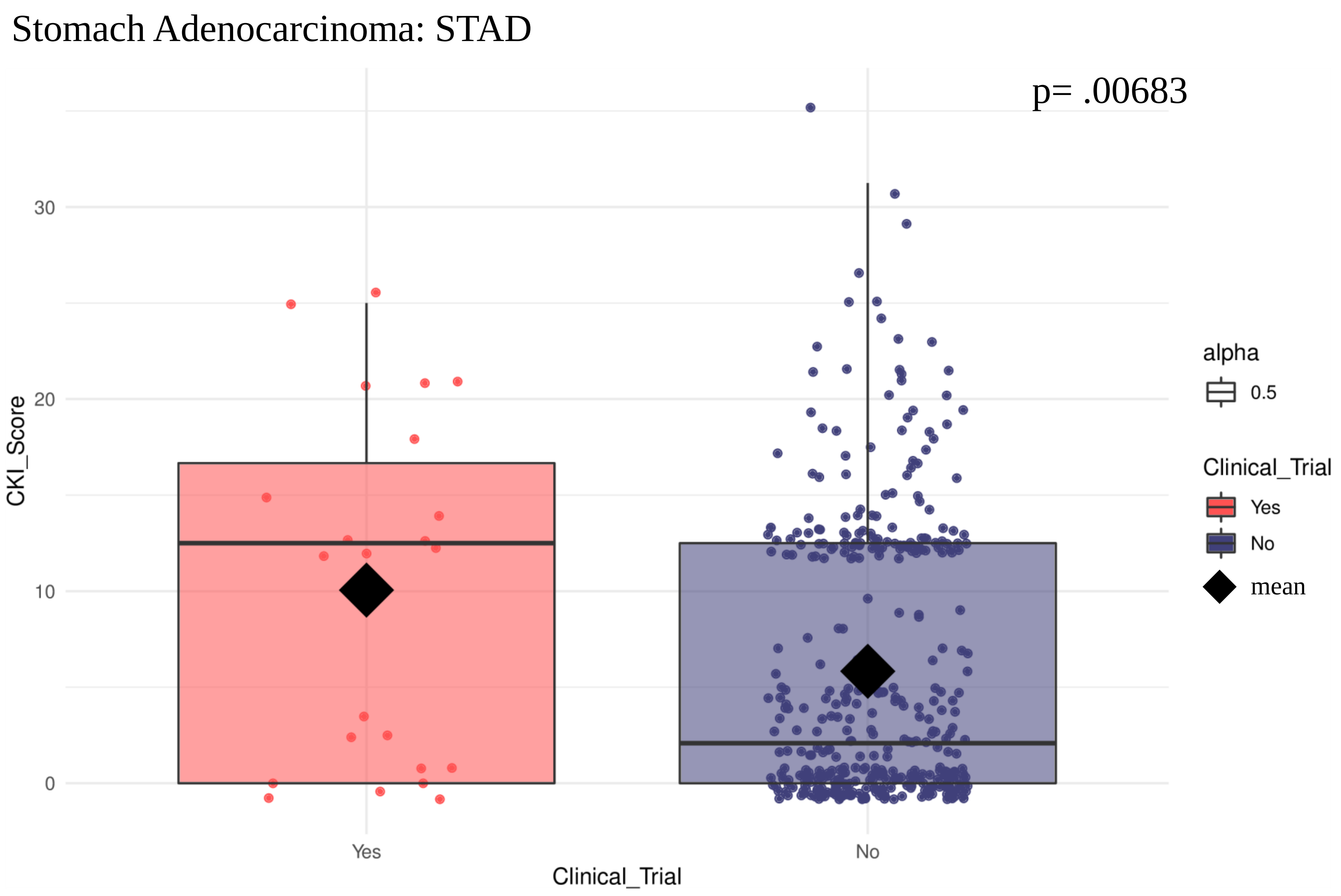
Stomach Adenocarcinoma: STAD
p= .00683
CKI_Score
30
20
10
0
Yes
No
Clinical_Trial
alpha
0.5
Clinical_Trial
Yes
No
mean

1P.

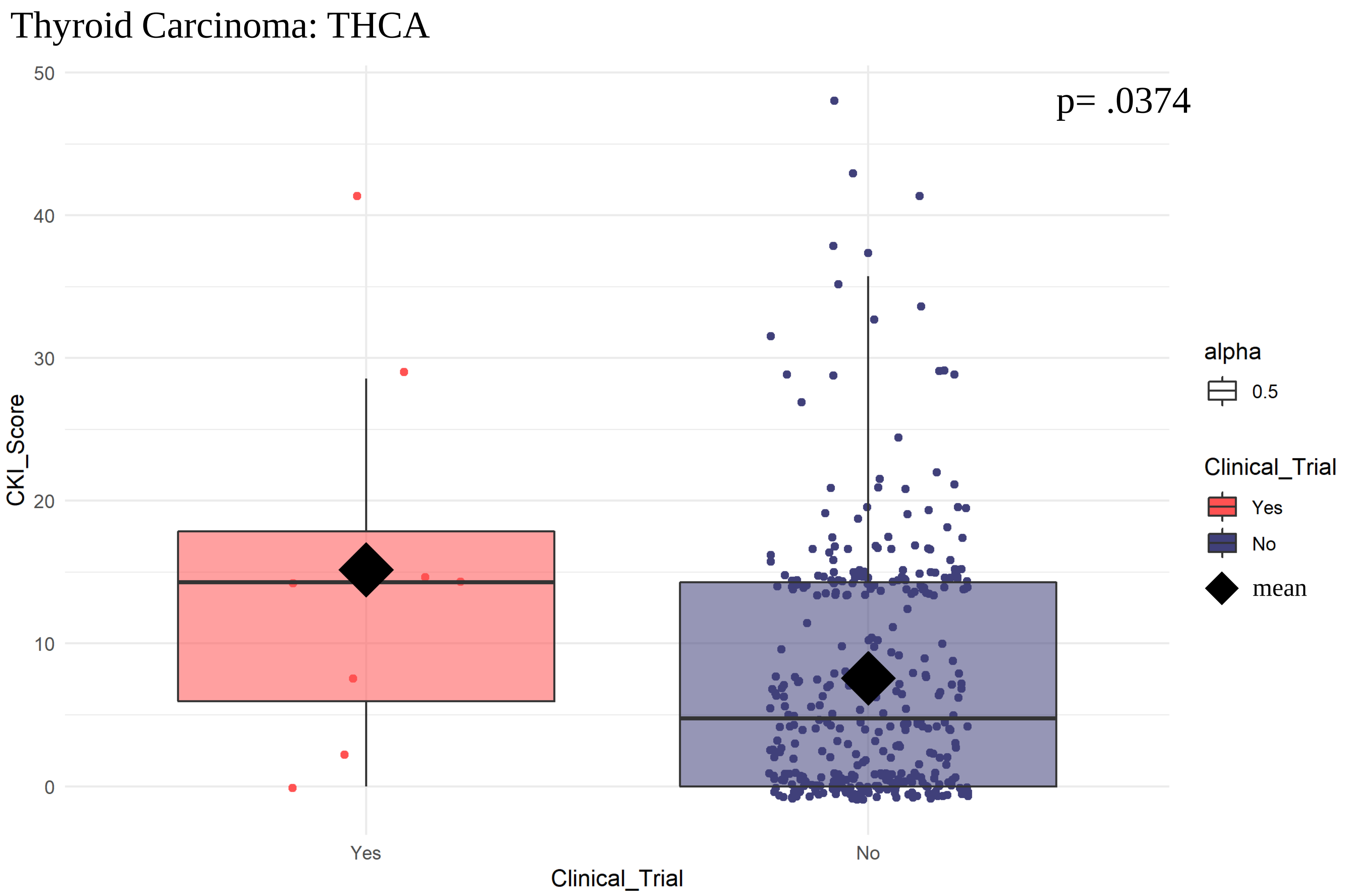
Thyroid Carcinoma: THCA
p= .0374
CKI_Score
Clinical_Trial
Yes
No
0
10
20
30
40
50
alpha
0.5
Clinical_Trial
Yes
No
mean

Supplemental Data 2 (A-N) CKI Score for MOA Targets. Related to Figure 5.

2A.

Average CKI Score per Cancer, TDL

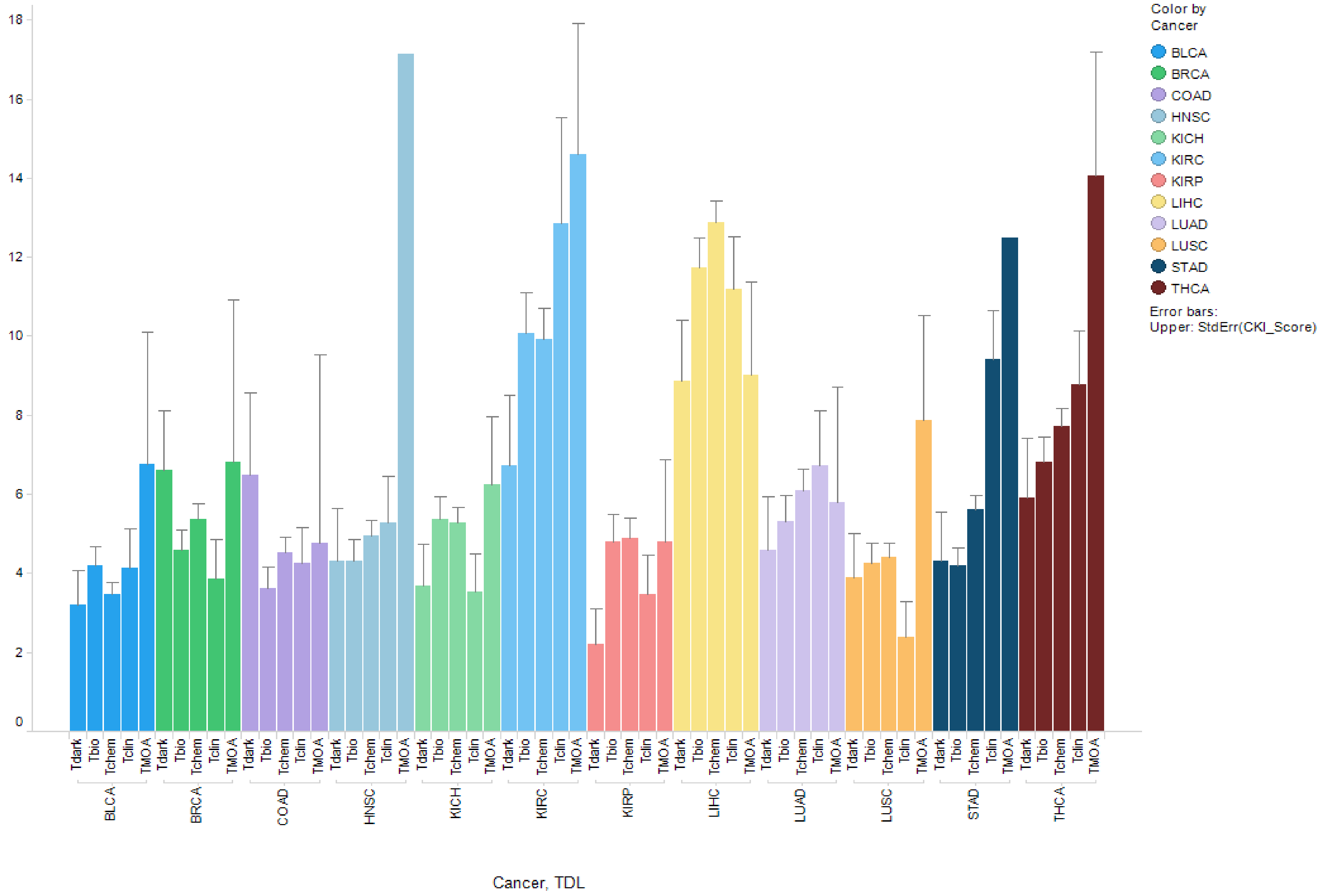

2B.

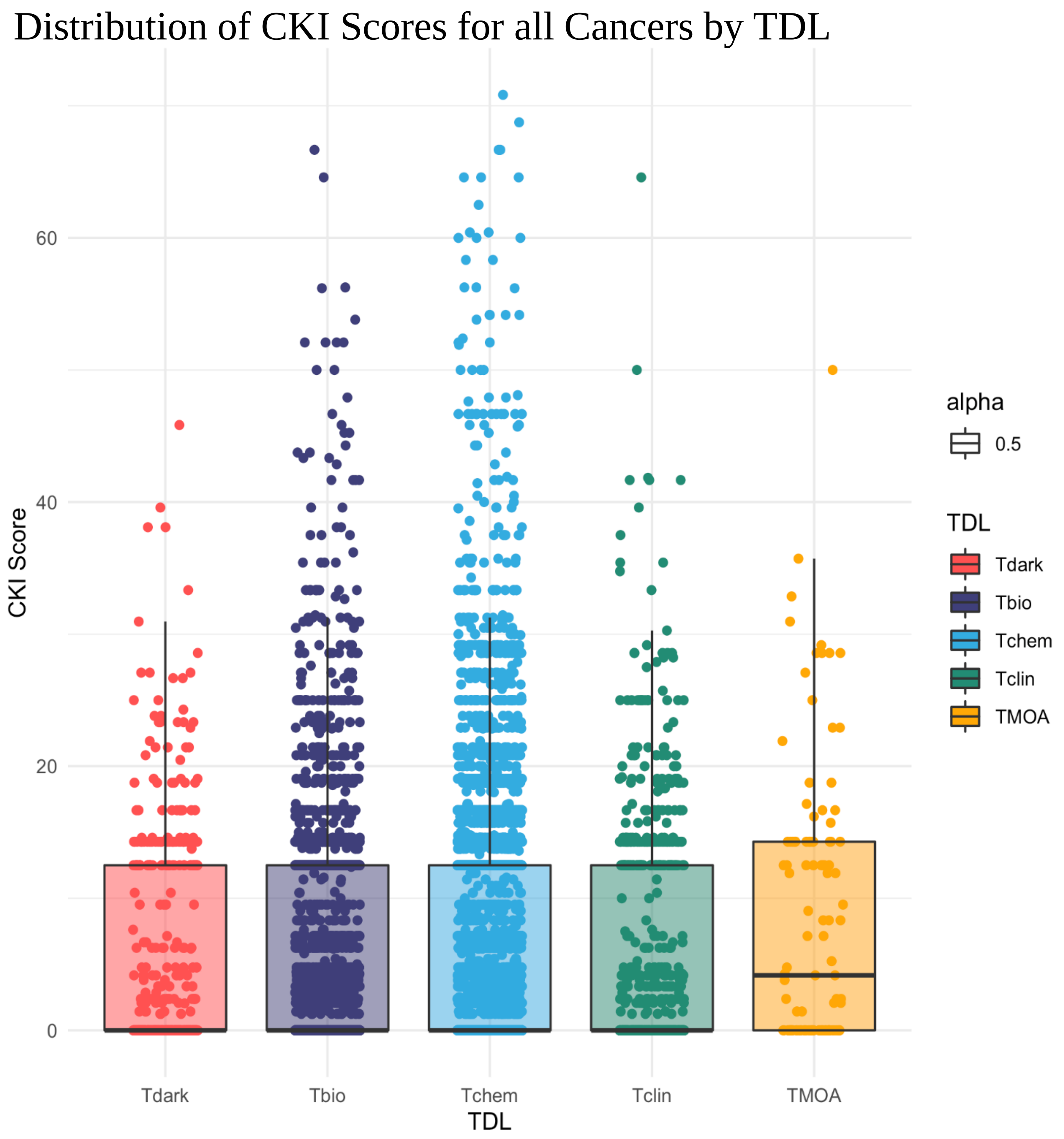
Distribution of CKI Scores for all Cancers by TDL
CKI Score
60
40
20
0
Tdark
Tbio
Tchem
Tclin
TMOA
TDL
alpha
0.5
TDL
Tdark
Tbio
Tchem
Tclin
TMOA

2C.

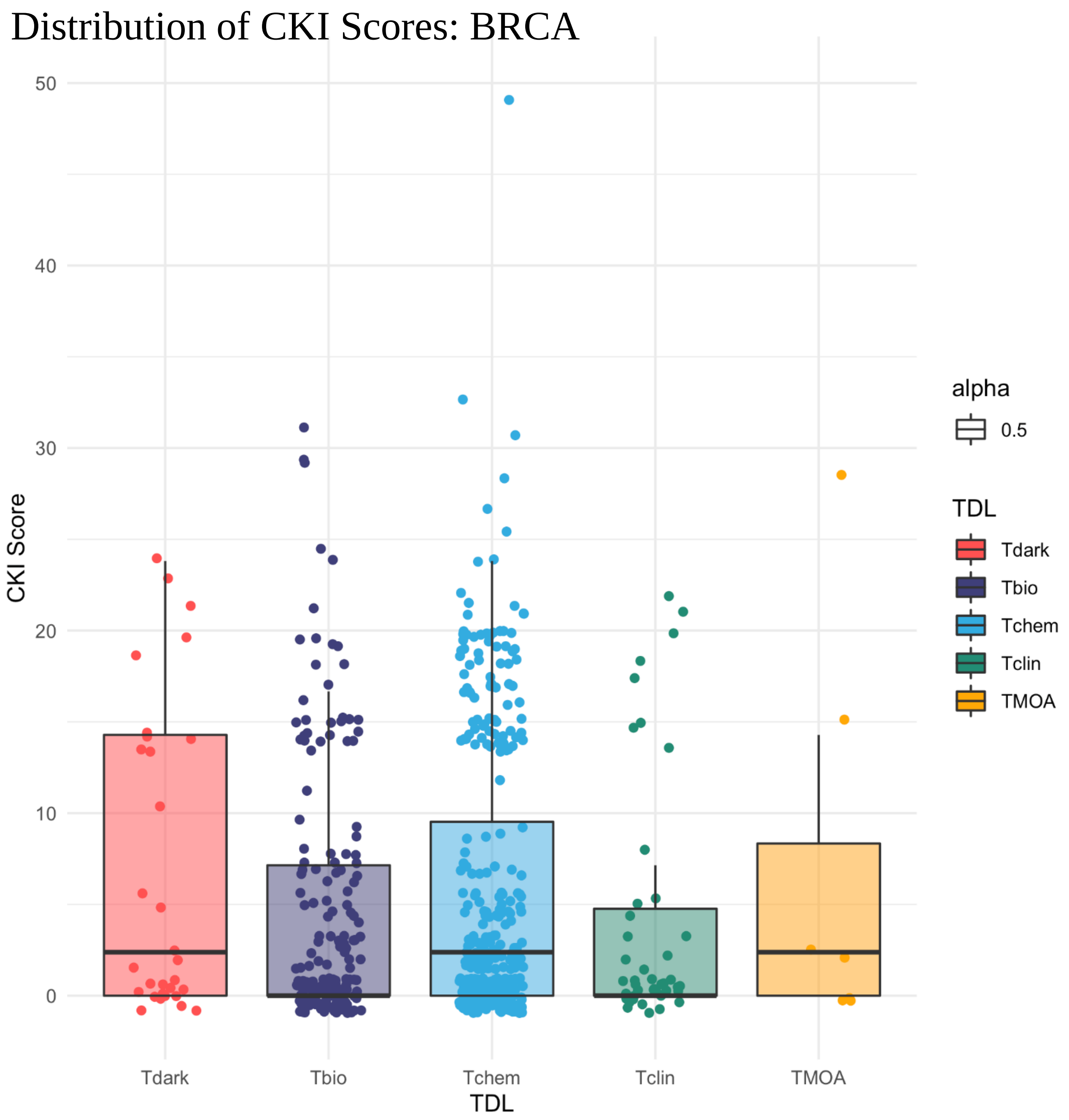
Distribution of CKI Scores: BRCA
CKI Score
50
40
30
20
10
0
Tdark
Tbio
Tchem
Tclin
TMOA
TDL
alpha
0.5
TDL
Tdark
Tbio
Tchem
Tclin
TMOA

2D.

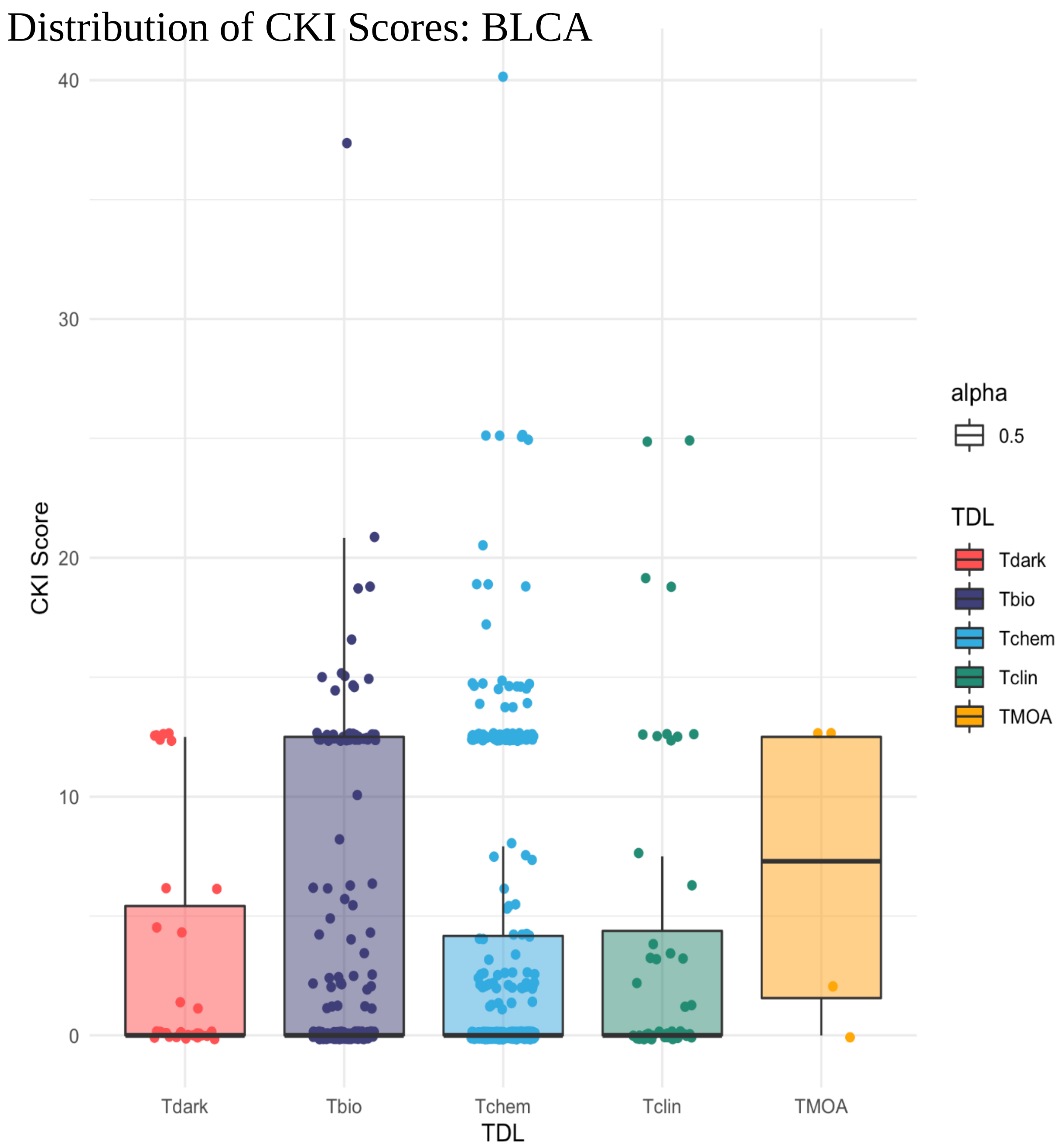

Distribution of CKI Scores: BLCA
CKI Score
40
30
20
10
0
Tdark
Tbio
Tchem
Tclin
TMOA
TDL
alpha
0.5
TDL
Tdark
Tbio
Tchem
Tclin
TMOA

2E.

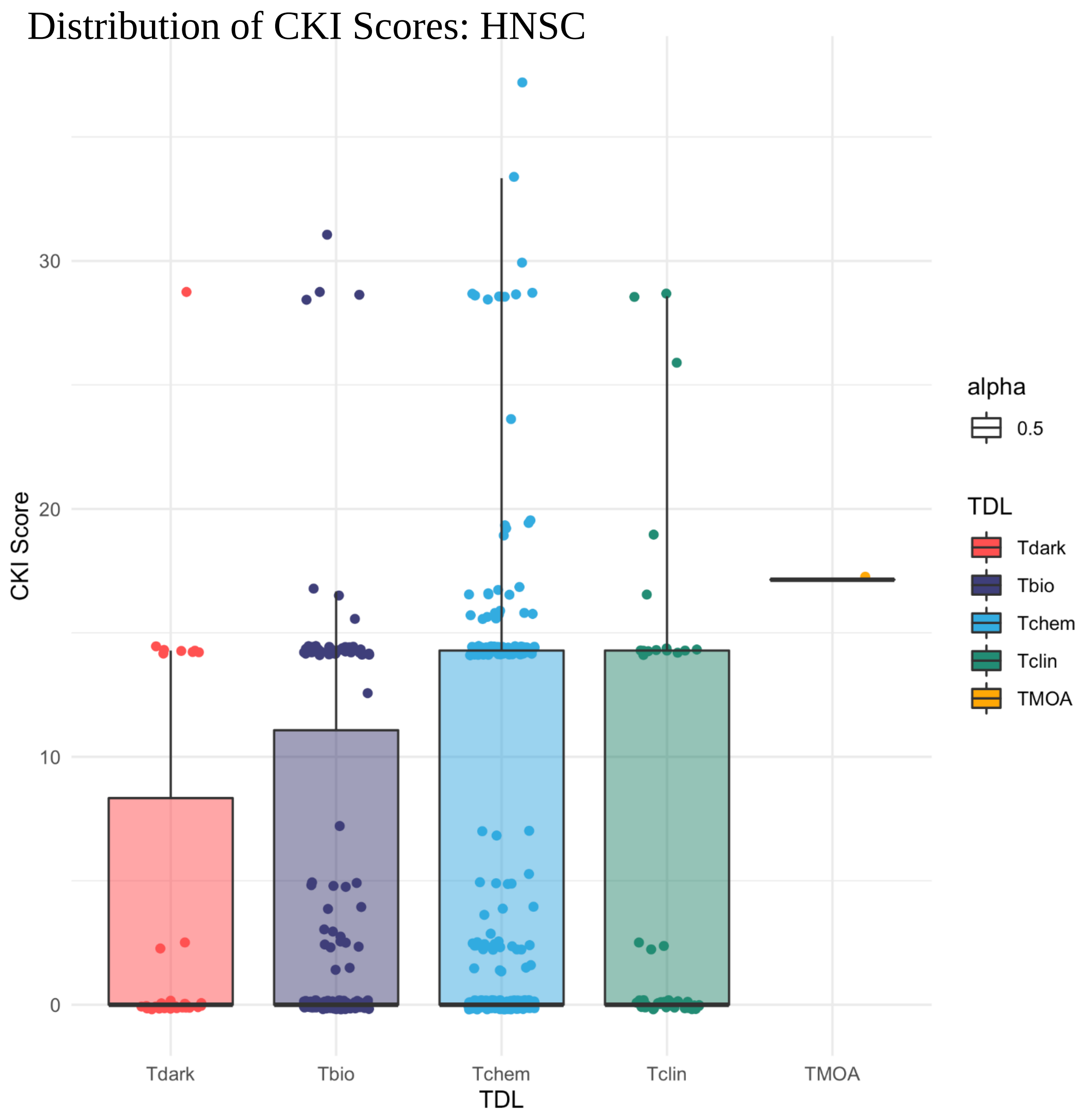

Distribution of CKI Scores: HNSC
CKI Score
30
20
10
0
Tdark
Tbio
Tchem
Tclin
TMOA
TDL
alpha
0.5
TDL
Tdark
Tbio
Tchem
Tclin
TMOA

2F.

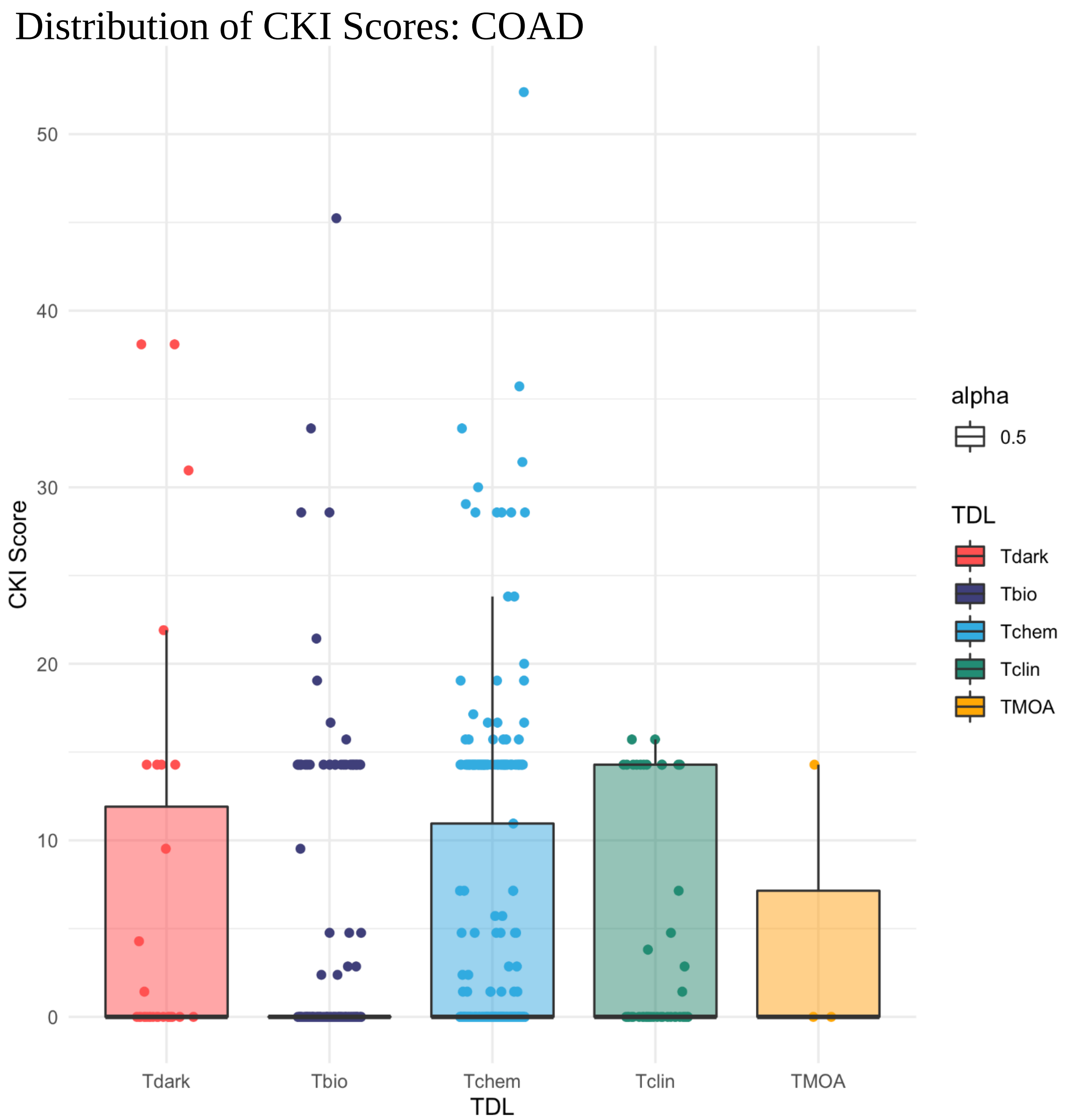

Distribution of CKI Scores: COAD
CKI Score
50
40
30
20
10
0
Tdark
Tbio
Tchem
Tclin
TMOA
TDL
alpha
0.5
TDL
Tdark
Tbio
Tchem
Tclin
TMOA

2G.

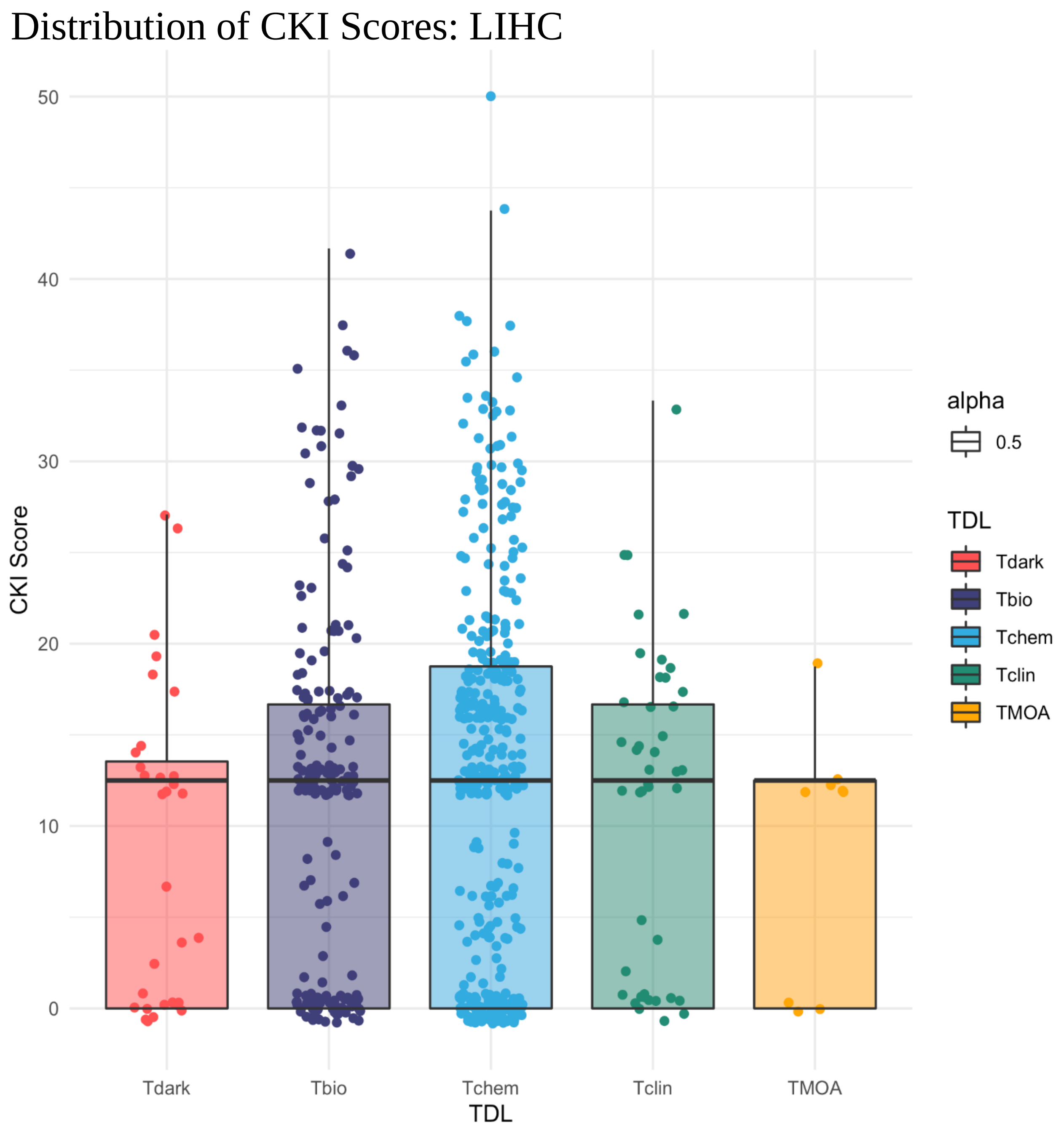
Distribution of CKI Scores: LIHC
CKI Score
50
40
30
20
10
0
Tdark
Tbio
Tchem
Tclin
TMOA
TDL
alpha
0.5
TDL
Tdark
Tbio
Tchem
Tclin
TMOA

2H.

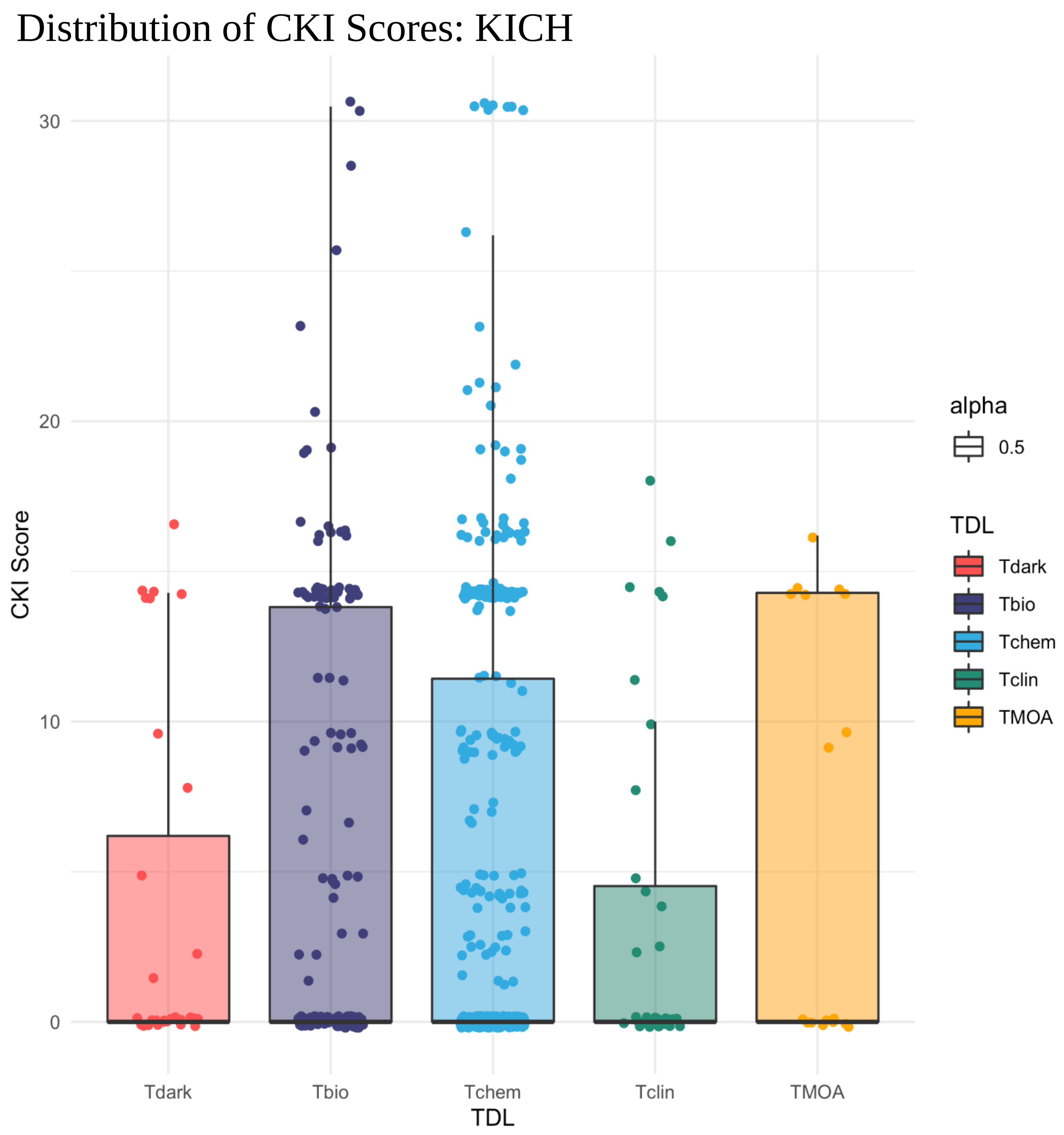
Distribution of CKI Scores: KICH
CKI Score
30
20
10
0
Tdark
Tbio
Tchem
Tclin
TMOA
TDL
alpha
0.5
TDL
Tdark
Tbio
Tchem
Tclin
TMOA

2I.

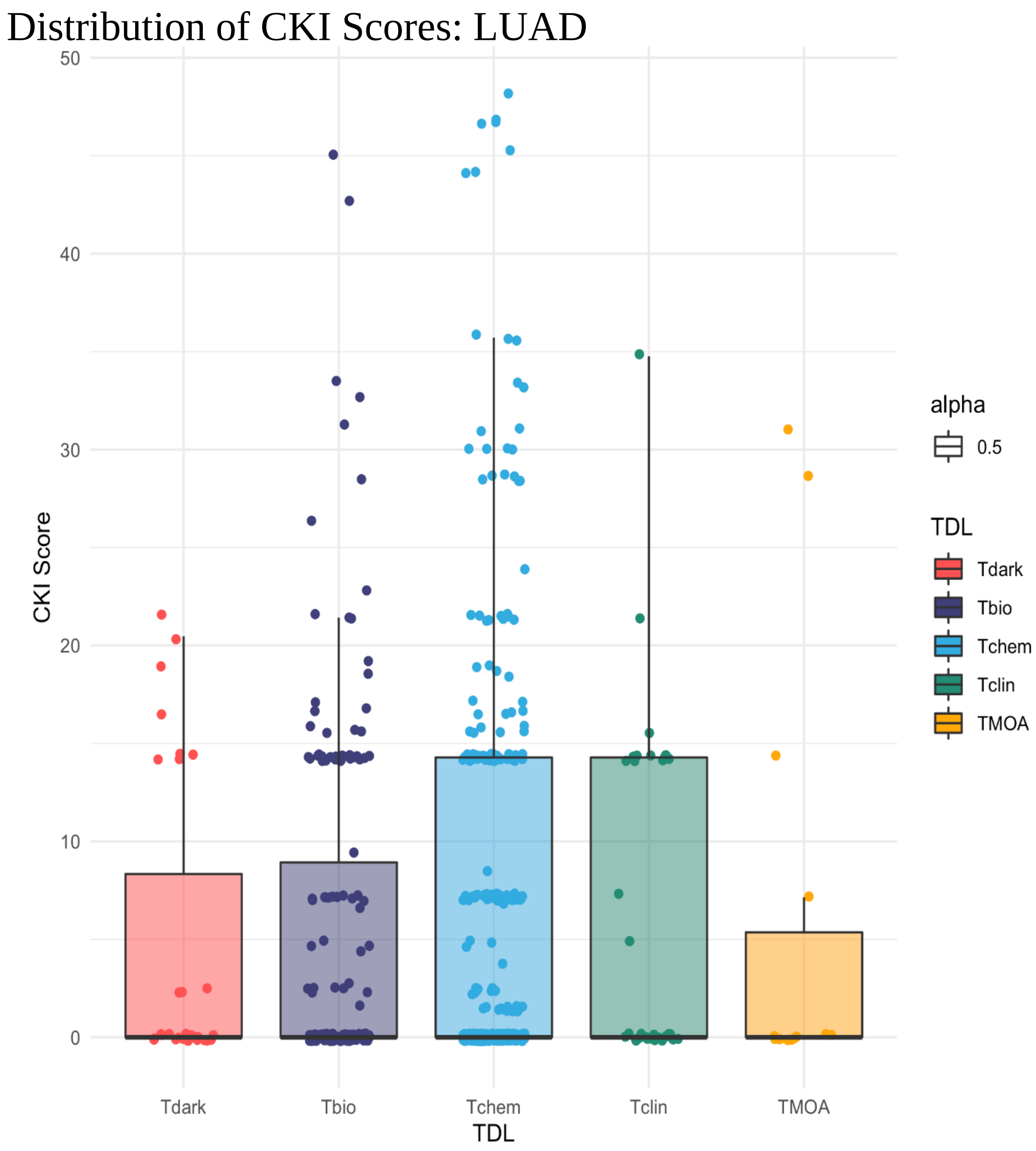

Distribution of CKI Scores: LUAD
50
40
30
20
10
0
CKI Score
Tdark
Tbio
Tchem
Tclin
TMOA
TDL
alpha
0.5
TDL
Tdark
Tbio
Tchem
Tclin
TMOA

2J.

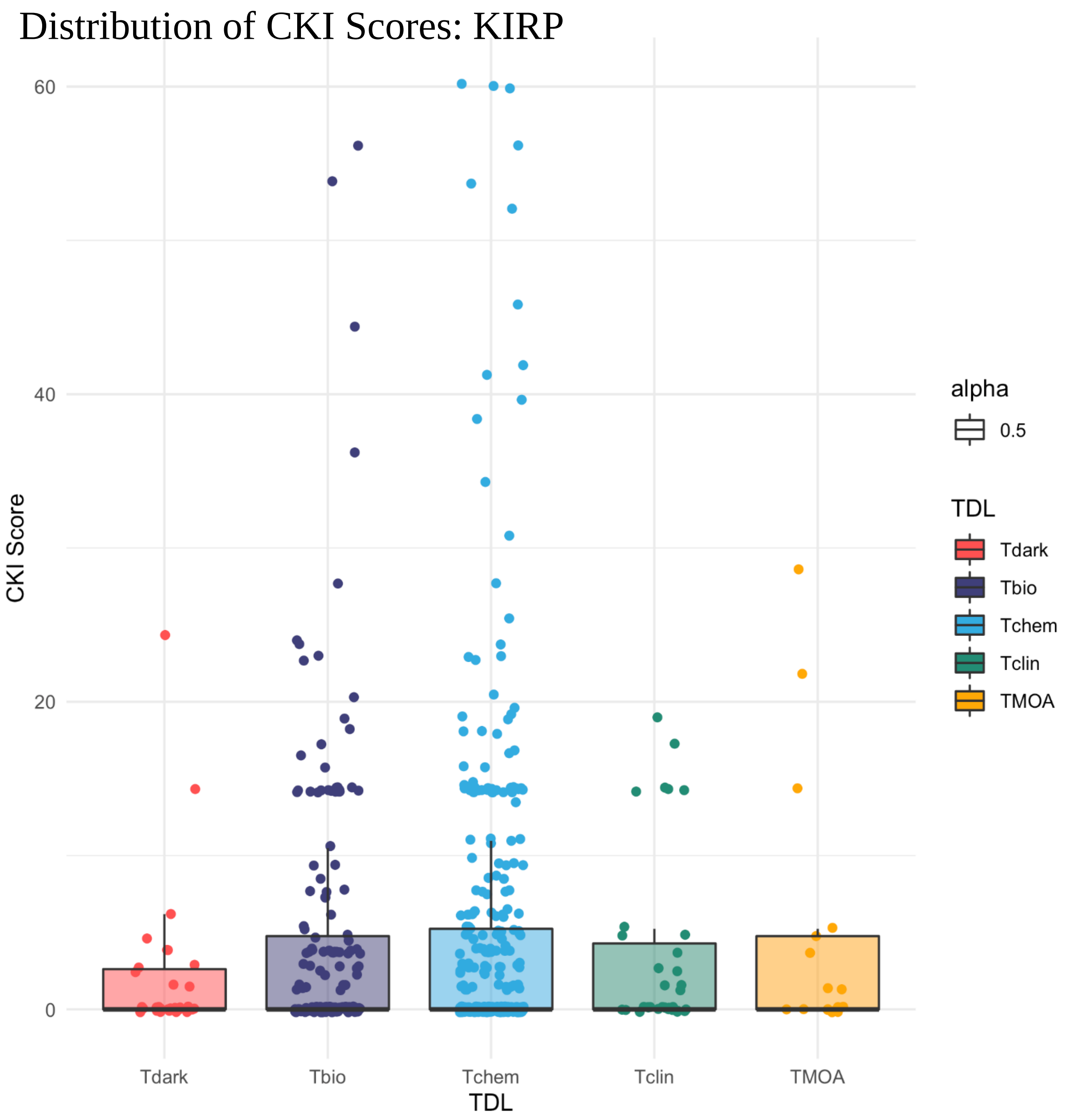
Distribution of CKI Scores: KIRP
CKI Score
60
40
20
0
Tdark
Tbio
Tchem
Tclin
TMOA
TDL
alpha
0.5
TDL
Tdark
Tbio
Tchem
Tclin
TMOA

2K.

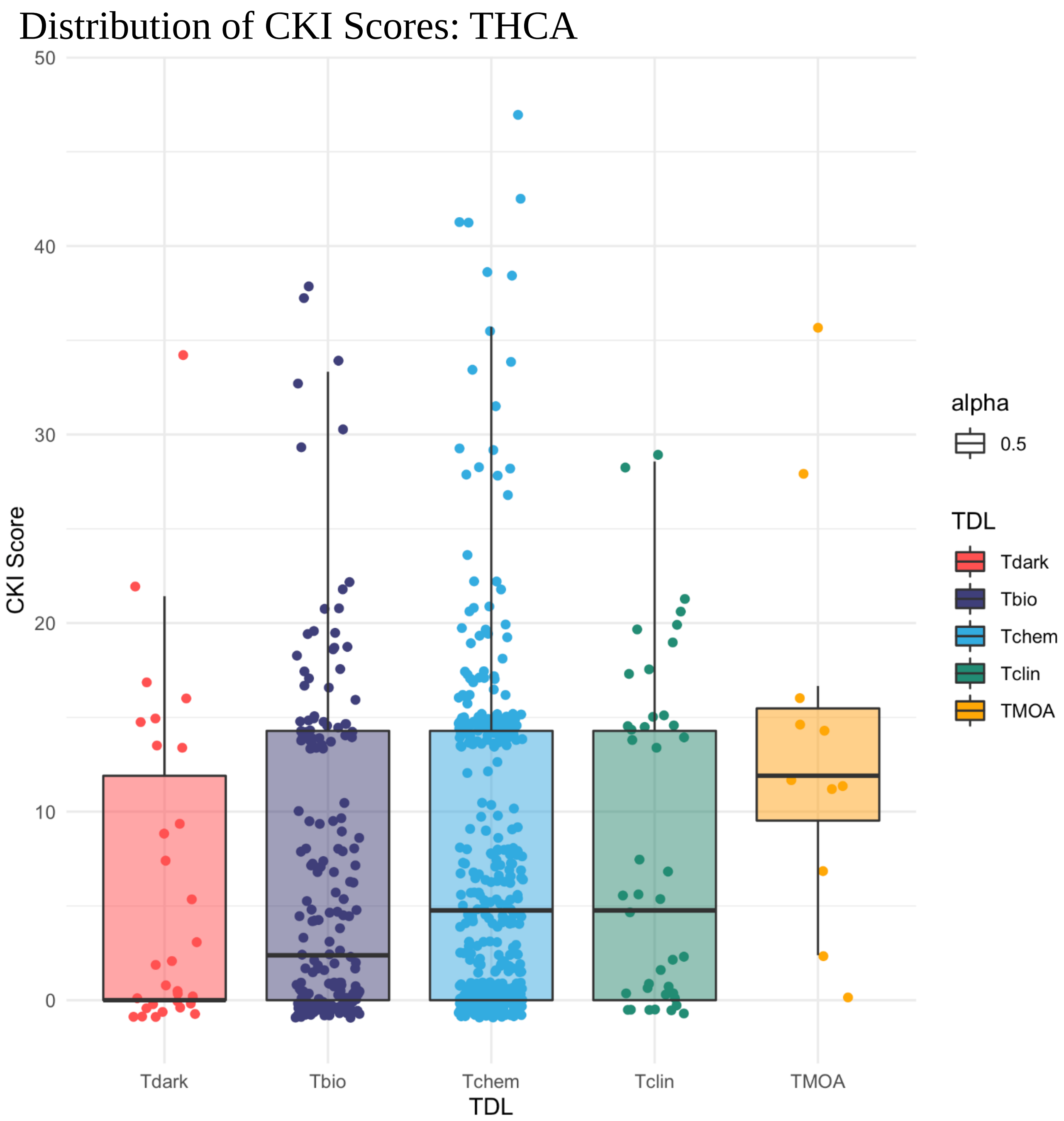
Distribution of CKI Scores: THCA
CKI Score
TDL
Tdark
Tbio
Tchem
Tclin
TMOA
0
10
20
30
40
50
alpha
0.5

2L.

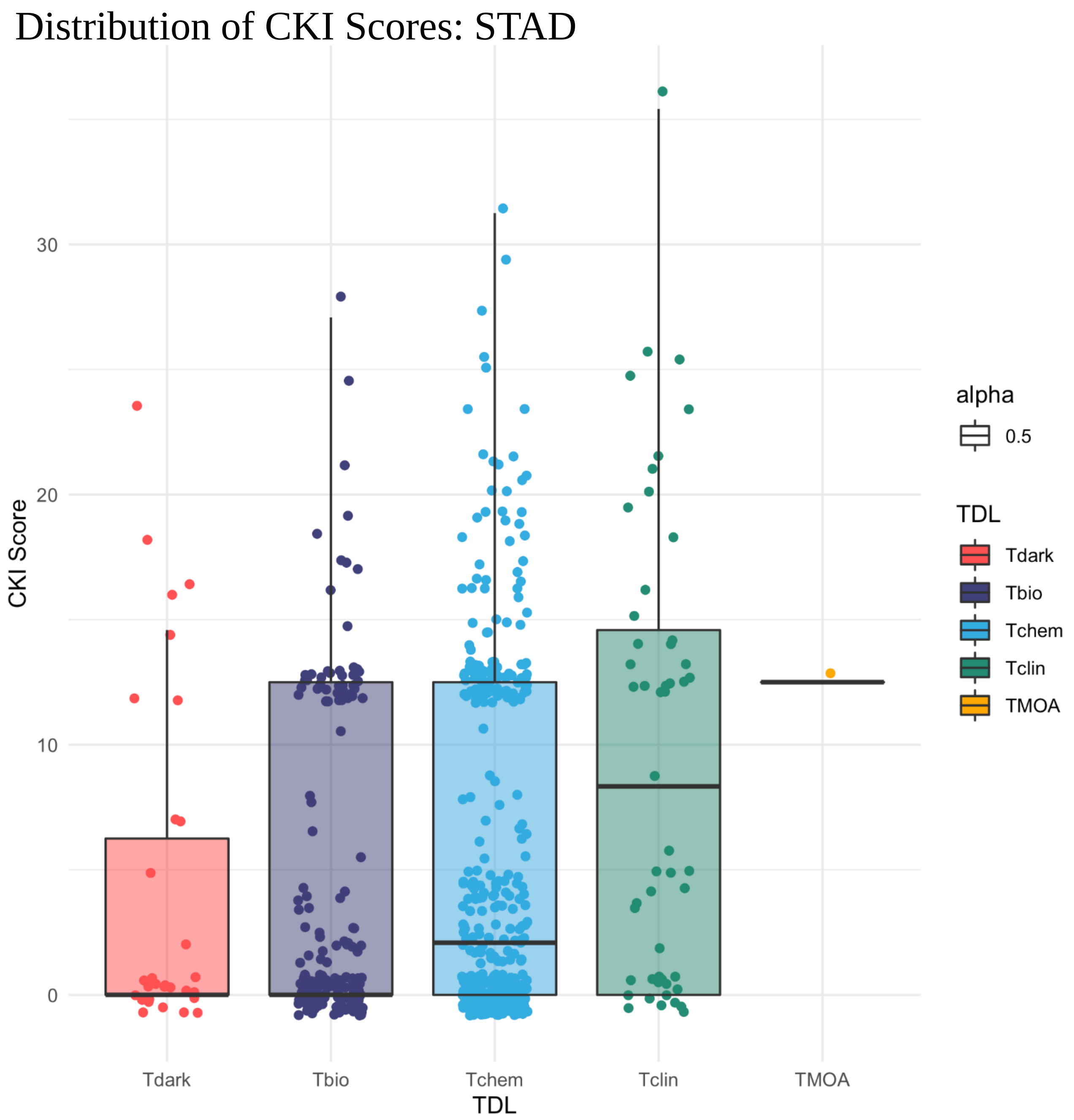

Distribution of CKI Scores: STAD
CKI Score
30
20
10
0
Tdark
Tbio
Tchem
Tclin
TMOA
TDL
alpha
0.5
TDL
Tdark
Tbio
Tchem
Tclin
TMOA

2M.

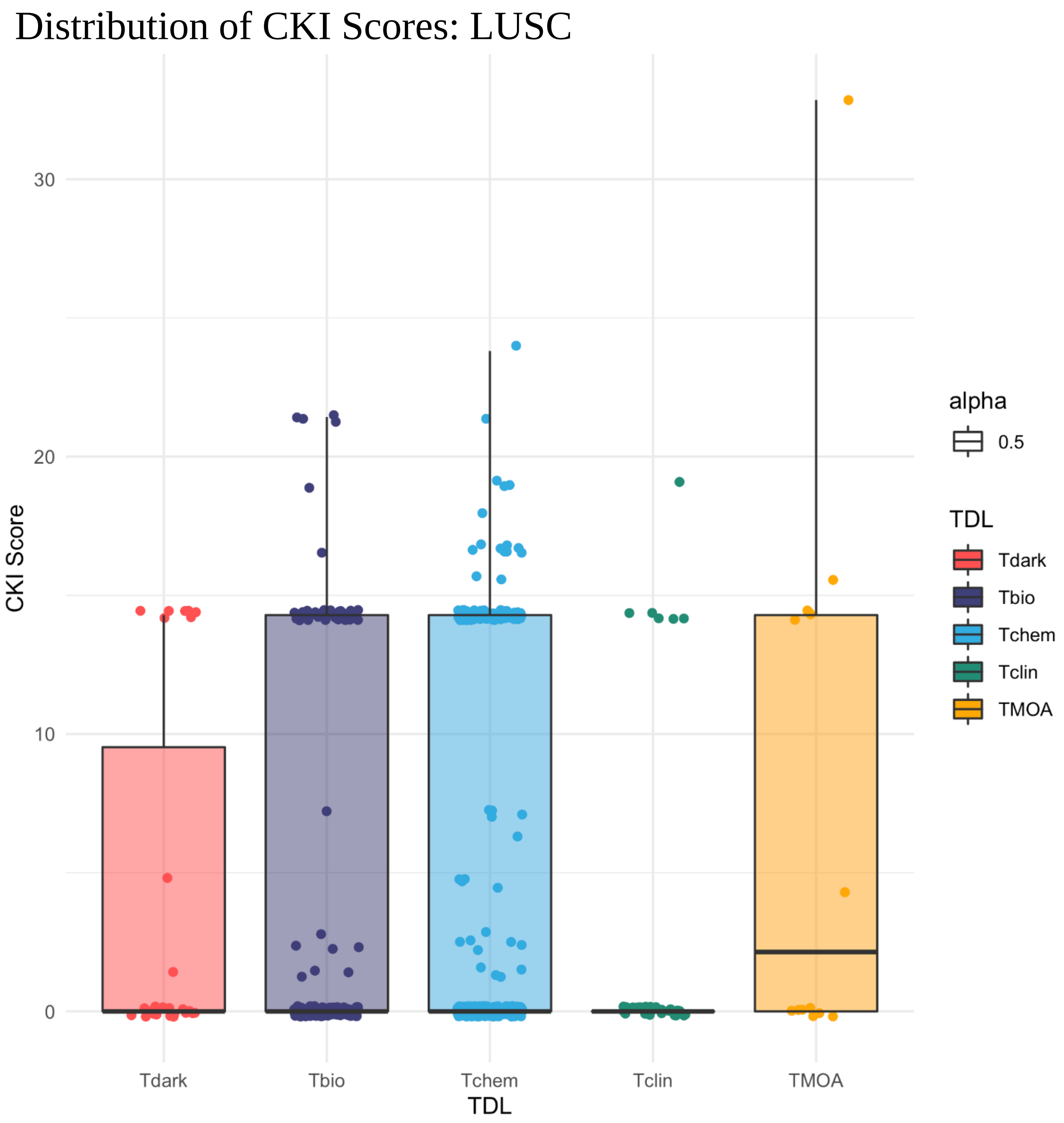

Distribution of CKI Scores: LUSC
CKI Score
30
20
10
0
Tdark
Tbio
Tchem
Tclin
TMOA
TDL
alpha
0.5
TDL
Tdark
Tbio
Tchem
Tclin
TMOA

2N.

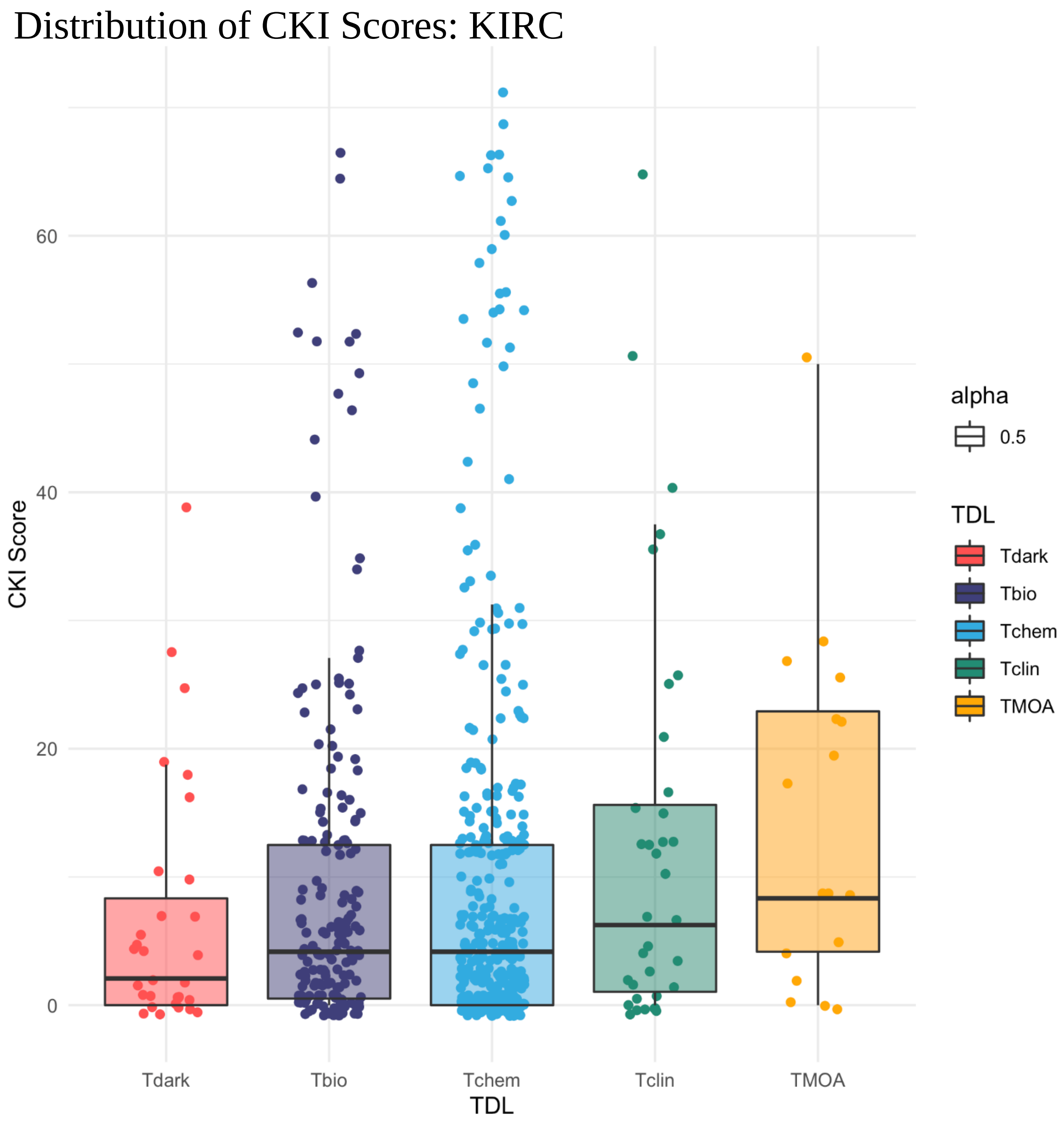
Distribution of CKI Scores: KIRC
CKI Score
60
40
20
0
Tdark
Tbio
Tchem
Tclin
TMOA
TDL
alpha
0.5
TDL
Tdark
Tbio
Tchem
Tclin
TMOA
