# Supplementary material for: The Clinical Kinase Index: A Method to Prioritize Understudied Kinases as Drug Targets for the Treatment of Cancer
Source: Cell Rep Med. 2020 Oct 20;1(7):100128. doi: 10.1016/j.xcrm.2020.100128 (PMC7659504; doi:10.1016/j.xcrm.2020.100128)
Supplement: Document S2. Article plus Supplemental Information [file mmc9.pdf]

Cell Reports
Medicine

Article

The Clinical Kinase Index: A Method to Prioritize Understudied Kinases as Drug Targets for the Treatment of Cancer

Graphical Abstract

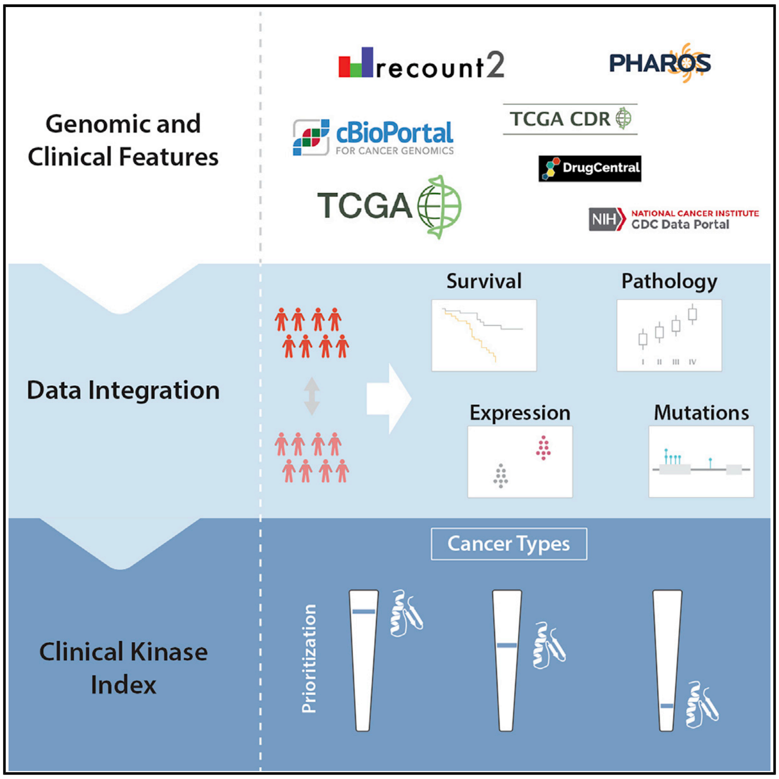

Highlights

- CKI is a novel method to prioritize dark kinases as cancer drug targets
- Expression of understudied kinases in tumors is prognostic of poor outcomes
- Dark kinases are likely clinically relevant cancer targets
- Cancer cell dependency correlates with tumor pathology and survival

Authors

Derek Essegian, Rimpi Khurana, Vasileios Stathias, Stephan C. Schürer

Correspondence

sschurer@med.miami.edu

In Brief

Essegian et. al conduct a kinome-wide pan-cancer analysis and provide an intuitive interface to prioritize understudied kinases as prospective novel cancer drug targets. While many of the approved kinase drug targets rank high in each cancer cohort, several understudied kinases also appear to be clinically relevant and warrant further exploration.

Essegian et al., 2020, Cell Reports Medicine 1, 100128
October 20, 2020 © 2020 The Authors.
https://doi.org/10.1016/j.xcrm.2020.100128

CellPress

Cell Reports Medicine

CellPress
OPEN ACCESS

Article

The Clinical Kinase Index: A Method to Prioritize Understudied Kinases as Drug Targets for the Treatment of Cancer

Derek Essegian,[1,4] Rimpi Khurana,[1,4] Vasileios Stathias,[1,2] and Stephan C. Schürer[1,2,3,5,*]
[1]Department of Pharmacology, Miller School of Medicine, University of Miami, Miami, USA
[2]Sylvester Comprehensive Cancer Center, University of Miami, Miami, USA
[3]Institute for Data Science & Computing, University of Miami, Miami, USA
[4]These authors contributed equally
[5]Lead Contact
*Correspondence: sschurer@med.miami.edu
https://doi.org/10.1016/j.xcrm.2020.100128

SUMMARY

The approval of the first kinase inhibitor, Gleevec, ushered in a paradigm shift for oncological treatment—the use of genomic data for targeted, efficacious therapies. Since then, over 48 additional small-molecule kinase inhibitors have been approved, solidifying the case for kinases as a highly druggable and attractive target class. Despite the role deregulated kinase activity plays in cancer, only 8% of the kinome has been effectively "drugged." Moreover, 24% of the 634 human kinases are understudied. We have developed a comprehensive scoring system that utilizes differential gene expression, pathological parameters, overall survival, and mutational hotspot analysis to rank and prioritize clinically relevant kinases across 17 solid tumor cancers from The Cancer Genome Atlas. We have developed the clinical kinase index (CKI) app (http://cki.ccs.miami.edu) to facilitate interactive analysis of all kinases in each cancer. Collectively, we report that understudied kinases have potential clinical value as biomarkers or drug targets that warrant further study.

INTRODUCTION

The human genome encodes about 634 kinases (pseudokinases included). However, as of 2019, only 49 kinases (8%) are currently primary targets of FDA-approved small-molecule cancer drugs[1–3] (Figure 1). Furthermore, 70% of these approved cancer kinase drug targets belong to the tyrosine kinase (TK) group. For several cancers, though, targeting the TK group has not been an effective strategy, despite overwhelming evidence of receptor TK dysregulation in those tumors.[4] For example, TK inhibitors (TKIs) have shown little to no clinical efficacy in the treatment of bladder, esophageal, prostate, brain, and stomach cancers.[4–8] While it has been firmly established that aberrant kinase activity indeed leads to cancer progression and metastasis in many cancers, researchers have not fully elucidated the ideal cancer-specific kinase targets or novel drug combinations to improve the standard of care.

To promote the study and validation of novel kinase targets, we herein describe a kinase-prioritization index using data from The Cancer Genome Atlas (TCGA) across 17 cancer types for researchers to use as a starting point for further investigation (Table S1 provides an overview of the available cancer data). By combining differential gene expression (DGE), Kaplan-Meier (KM) survival, and mutational hotspot and clinical/pathological correlation analyses, we have developed a scoring system, the clinical kinase index (CKI), to prioritize *clinically* relevant kinase targets for each cancer cohort (Figure 2). We define "clinically relevant" as having a correlation or relationship to critical clinical benchmarks, such as tumor grade and American Joint Committee on Cancer (AJCC) tumor, node, metastasis (TNM) staging.[9] In short, we have analyzed and highlighted kinases whose mRNA expression levels appear to be prognostic and associated with the progression of cancer. Since kinase activity does not always correspond with mRNA expression levels (e.g., mTOR oncogenic activity is due to an increase in activation via phosphorylation[10]), we also leveraged TCGA genomic data and prioritized kinases that confer a selective advantage to tumor development as measured by the accrual and clustering of mutations at specific regions of their amino acid sequence. Moreover, by integrating our data with external target annotation resources, we evaluated the CKI scores based on a number of clinical classifications, such as target development level (TDL),[11] which classifies a target based on available target validation knowledge (Tclin, Tchem, Tbio, and Tdark); kinase family class (which corresponds to phylogenecity and substrates); clinical trial data; and MOA (mechanism of action) of approved drugs for each cancer type.[1,11–13]

With over 175 kinase drugs currently in clinical trials, new targets are being evaluated, including AKT, aurora kinases, CHEK1, and CDK1.[14] Despite the large number of drugs that are being

This is an open access article under the CC BY license (http://creativecommons.org/licenses/by/4.0/).

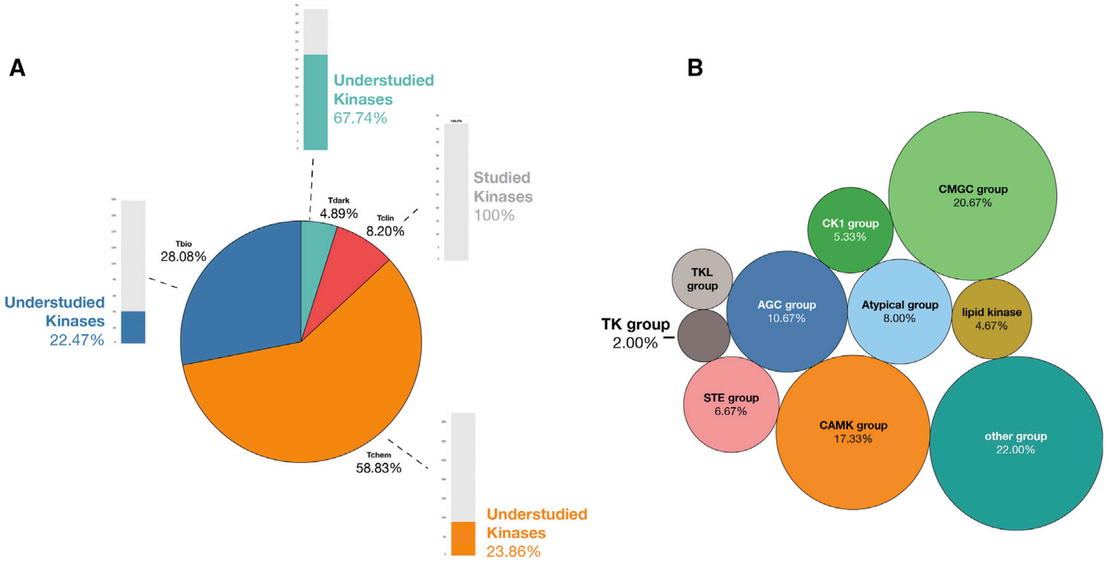

Figure 1. Overview of Kinase TDLs

(A) There are 634 kinases annotated on Pharos. In total, 8.2% are considered Tclin, and 151 of 634 are considered "understudied"; these represent kinases that bear Tdark, Tbio, and Tchem annotations.

(B) Certain groups of kinases are historically understudied. The CMGC group, CAMK group, and "other" group kinases are enriched for understudied kinases, while TKs have been extensively explored.

investigated, the majority of trials are for well-known, previously approved kinase targets such as EGFR, VEGFR, phosphatidylinositol 3-kinase (PI3K), and mTOR.[14] Nevertheless, there are still no small-molecule drugs that target kinases in the calmodulin-dependent protein kinase (CaMK), CK1, or AGC groups of kinases as their primary target (Ki < 10 nM), notwithstanding increased evidence for their clinical relevance in cancer.[15,16] The extent of such a misrepresentation has been highlighted by the NIH-funded Illuminating the Druggable Genome (IDG; https://druggablegenome.net/) project, where analysis concludes that 23.8% (151) of the 634 kinases are "understudied," as they lack sufficient GeneRIFs, antibodies, citations in the literature, and potent chemical probes.[11] Consistent with this is the fact that the current kinase inhibitors target not only a narrow range of targets, but also a narrow range of pathways including angiogenesis, cell adhesion, immune system signaling (cytokine, T cell receptor, B cell receptor), and anti-apoptotic pathways.[17] For example, all kinase inhibitors for renal cell carcinoma target angiogenic pathways.[18] It is likely that the most optimal strategy for treating cancers is targeting multiple orthogonal pathways that work in a synergistic manner, as opposed to targeting kinases with overlapping pathways.[19–21] It has already been shown that this strategy may prevent or reduce the incidence of resistance pathways and kinome reprogramming, which is inevitable upon singular treatment with a highly specific kinase drug such as the EGFR inhibitor, lapatinib.[22,23] The CKI is a tool that may be used to facilitate the exploration of clinically relevant new kinase drug targets for this purpose.

Along with this report, we have made available the CKI app (http://cki.ccs.miami.edu/) to access, interactively explore, download, and analyze all data and results of this study. With this study, we provide a resource for the scientific community where the clinical relevance of kinase genes across solid-tumor cancers can quickly be evaluated, especially for understudied kinases and cancers for which no approved first-line kinase therapy exists.

RESULTS

The CKI Predicts Clinically Validated Kinase Targets

The CKI (http://cki.ccs.miami.edu/) serves the purpose of ranking and categorizing the clinical relevance of the entire kinome, with a special focus on understudied and dark kinases (Figure 3). To generate a CKI score for each kinase in each cancer cohort, four parameters were taken into consideration: DGE, KM survival, mutational hotspots, and clinical-pathological features. For each DGE, KM survival, and mutational hotspot, kinases received a score of 1 if there was statistical significance (see Method Details). Clinical and pathological data varied by cancer cohort; thus, a clinical score was generated using ANOVA analysis to determine if kinase expression correlated with progression through stage or grade. For each clinical-pathological parameter, there was a maximum score of 1. The raw CKI score for each kinase in a cancer cohort is the sum of the scores from DGE, survival, mutational analysis, and clinical scores. Raw scores were then divided by the total possible score and converted to percentages. For comparison across cancer cohorts, a rank-ordered list was also generated, which is available for analysis on the CKI app.

We have observed several trends in the CKI that may underscore the validity of our model and prioritization system. First, the CKI accurately prioritizes currently in-trial or approved drug

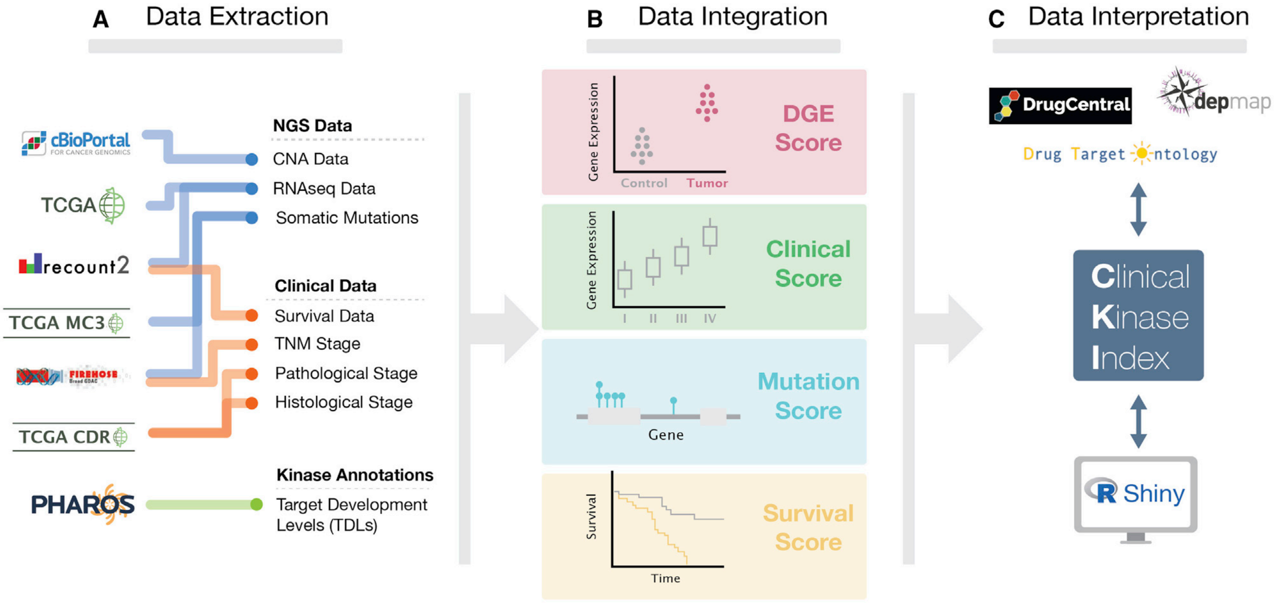

Figure 2. Outline of the CKI Workflow

(A) Data were extracted from multiple sources for curation, filtering, and normalization.

(B) mRNA levels and mutational hotspots were analyzed to generate DGE analyses and mutational frequencies. mRNA levels were also correlated with survival and clinical/pathological outcomes. Various statistical methods were employed, and all significant correlations were scored.

(C) Final CKI scores were generated and mapped to other data annotations for further analysis and interpretation.

targets for several cancers in the TCGA. As such, the average and median CKI scores for kinases that have been under clinical investigation are statistically significantly higher than those that have not in BRCA, head and neck squamous carcinoma (HNSC), renal clear cell (KIRC), lung adenocarcinoma (LUAD), lung squamous carcinoma (LUSC), rectal adenocarcinoma (READ), stomach adenocarcinoma (STAD), and thyroid carcinoma (THCA) cancers ($p < 0.05$; Table S2; Data S1A–S1P). Furthermore, kinases that are MOA targets for approved drugs have significantly higher average and median CKI scores than non-MOA targets across all cancers ($p < 0.0001$) ($p < 0.05$ for select cancer cohorts; Data S2A–S2N; Table S2). These results generally suggest that less-studied kinases that rank as high as clinical kinases should be prioritized for further validation and clinical investigation. The majority of the high-scoring targets have been or are currently under investigation. For example, breast tumor kinase (*PTK6*) is the highest-ranking kinase for BRCA. This kinase is regarded as the key regulator in the oncogenic transformation of breast cancer, is overexpressed in >80% of breast tumors, and is a highly attractive drug target[24–26] but has yet to enter clinical study. Other high-ranking kinases include those that are already in clinical trial or are FDA-approved targets, including PI3K kinases, MAP kinases, cyclin-dependent kinases, and aurora kinases. In the LUAD cohort, *EGFR* and *MEK1* rank highly; they represent targets of an approved lung cancer drug and compounds under investigation in several clinical trials.[27–30] Similarly, *MET* and *RET* are among the high-scoring Tclin kinases for thyroid cancer. On average, we find that Tclin kinases had higher CKI scores, suggesting that many of the most clinically relevant or prognostic cancer kinases have already been studied extensively or are targets of approved drugs. The highest-scoring kinase in all datasets is *PLK1* (Tchem), with a CKI of 70.83 in kidney renal papillary cancer (KIRP). *PLK1*, like other Tchem kinases, ranks very highly and is an attractive target that has been under clinical investigation.[31,32] *PLK1* is overexpressed in a number of cancers, and its activity has been linked to tumor growth, metastasis, and drug resistance.[31] *AURKA* and *AURKB* also consistently scored high across every cancer cohort, as did other cell-cycle- and mitoses-related kinases (*BUB1B*, *BUB1*, *CDK1*).

In addition to Tclin kinases, multiple understudied kinases exhibit a high CKI score, thus indicating they are likely clinically relevant targets. Understudied kinases rank among the top 20 kinase genes for every cancer cohort scored. Several of these kinases appear to have mRNA levels that are prognostic of survival and tumor progression in multiple cancers. For example, *ERN2* (Tbio) is the second-highest-scoring kinase in BRCA and cholangiocarcinoma (CHOL) and sixth in LIHC. Because of its understudied nature, there are little to no cancer-related publications available to assess the literature evidence of *ERN2* as a potential target. *ERN2* does code for the protein IRE1β, which is part of the unfolded-protein stress response pathway.[33] The unfolded protein response (UPR) pathway is a pro-survival pathway that is hijacked by cancer cells and thus has been a topic of discussion in the context of drug development.[34] Other understudied kinases that score favorably in the majority of cancer cohorts include *PKMYT1* (Tchem), *DCLK3* (Tchem), *BRSK1* (Tchem), *ADCK5* (Tdark), and *LMTK3* (Tbio).

To compare the CKI scores across each cancer cohort, we performed a Spearman correlation rank analysis (see Method

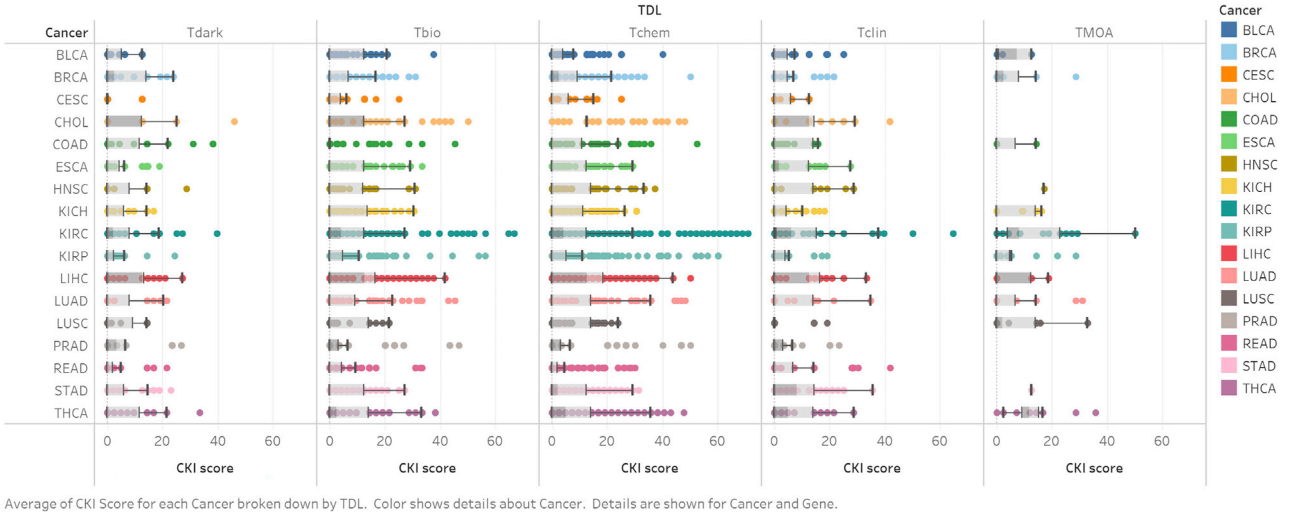

Figure 3. CKI scores by TDL and TCGA Cancer Cohort
Most cancers have average Tclin or Tchem scores that are significantly higher than the other TDLs. COAD, BRCA, LUSC, and PRAD have Tdark kinases that score, on average, higher than Tclin kinases. Furthermore, cancer-specific MOA targets tend to score the highest in the CKI. See also Table S2.

Details). Correlation between similar cancer cohorts based on tissue was the strongest, with LUSC and LUAD having significant overlap in top-ranking kinases, as was the same in KIRC with KIRP and colon adenocarcinoma (COAD) with READ (Figure 4). The top 25% of differentially overexpressed kinases in each cancer were also used as input for MSigDB[35] (gene set enrichment analysis) to compare kinase gene set enrichment overlap between each cancer. Spearman correlation using rank-ordered gene sets shows varying degrees of overlap among all cancer cohorts, but the strongest overlap was between LIHC and CHOL (85 gene sets in common). These data suggest that while many of the same key players are involved in the progression of multiple cancers, unique kinases emerge in each cohort. All cohorts were highly enriched for genes in the "FIRESTEIN_PROLIFERATION" and "MODULE_244" gene sets, which are genes required for the proliferation of colon cancer cells and genes responsible for DNA damage repair, respectively.[36]

CKI Scores Are Supported by Achilles DepMap Data

Project Achilles and the Dependency Map (DepMap)[37] are efforts to identify genes essential for cancer cell proliferation and survival. Combining RNAi and CRISPR systematic loss-of-function screens in over 700 cancer cell lines for over 17,000 genes, researchers were able to obtain gene-level "dependency" scores while accounting for off-target shRNA/Cas-9 effects and other molecular features using the DEMETER2 computational method.[37] We sought to benchmark the predictivity of our clinical kinase score against this experimentally derived data to explore the hypothesis that cancer kinase dependency in cell models correlates with clinical and pathological features associated with the dysregulation and overexpression of these kinases. Although we would not expect cell line data to perfectly correlate with clinical data, there should be some indication of clinical relevance extrapolated from cellular dependency. We used three independent datasets obtained from the DepMap portal to carry out these analyses: combined RNAi (Broad, Novartis, and Marcotte), shRNA Achilles (Avana), and CRISPR (Sanger). Each set had a different number of cell lines and disease models represented and a differing number of kinases annotated. For example, 565 of the 634 kinase genes in our dataset were present in the Achilles database. Of the 69 kinases that were not present in Achilles, 21 (30%) were understudied, which further underscores the inherent bias against these genes. Kinase genes were extracted from the datasets and were annotated as "dependent" if the dependency score was < -1 (a cutoff defined by all DepMap studies[37]) and "not dependent" if the dependency score was > -1. Kruskall-Wallis tests (one-way ANOVA) were performed to determine if there was a significant difference in the distribution of mean CKI score in each cohort (dependent versus non-dependent) in the combined RNAi, Achilles, and Sanger datasets per disease type. Cell lines were grouped by tissue type: for example, all cell lines annotated "lung" were used to analyze CKI scores in both the LUSC and LUAD TCGA cohorts. Similarly, all cell lines annotated "kidney" were analyzed with kidney chromophobe carcinoma (KICH), KIRC, and KIRP cohorts. The results support the hypothesis that gene dependency relates to clinical relevance. "Dependent" kinases score significantly higher on the CKI than "not dependent" kinases for many cell lines, across all cancers and datasets (p value < 0.05) (Figure 5; Table S3). Of note, some Tclin kinases in our model scored low on the CKI but had significant dependency scores. Further investigation revealed that cyclin-dependent kinases (CDKs) tend to score "low," as mRNA levels typically do not correspond with activity in tumors. CDKs, as their name suggest, have activity that is dependent on the abundance of cyclins. Also, as noted above, kinases such as mTOR have activity that is related to their post-translational modification. Despite these outliers, we can make the case that our kinase target ranking based on clinical phenotypes has a strong relationship with cancer-cell dependency in validated cancer cellular models.

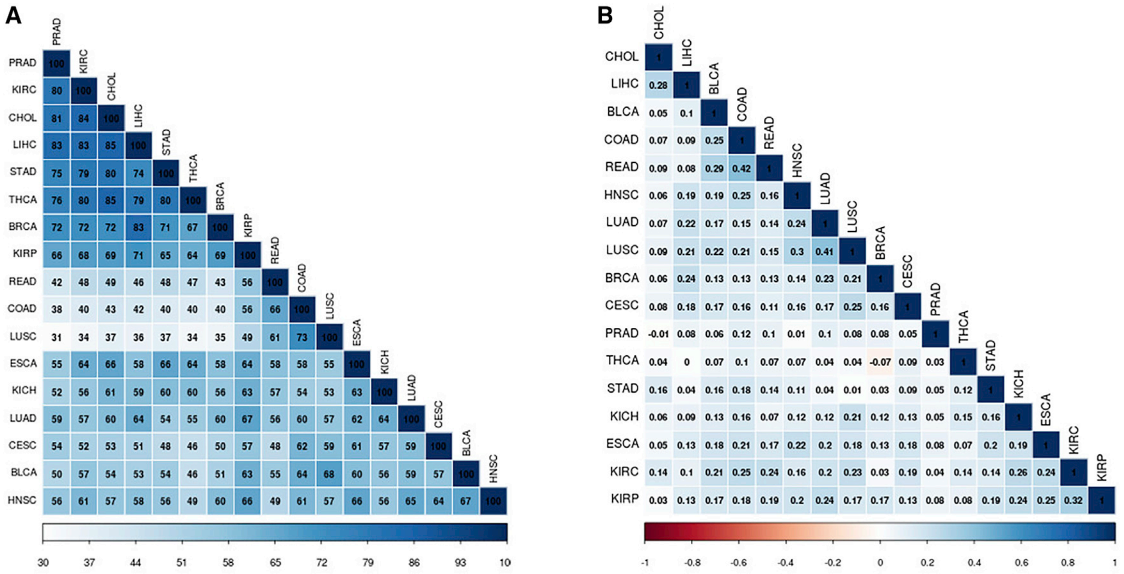

Figure 4. Overlap Analysis between Cancer Cohorts

(A) Gene set enrichment analysis was performed on the top-25%-scoring kinases for each cancer cohort using MSigDB. Pairwise overlap of 100 gene sets was calculated. Cohorts that share the highest degree of gene set enrichments include LIHC and CHOL, KIRC and CHOL, and THCA and CHOL.

(B) Spearman-rank correlation between CKIs of each cancer cohort. The greatest degree of correlation was present between cancers of the same tissue, including COAD and READ or LUAD and LUSC. THCA and BRCA had a slight negative correlation, suggestive of opposing clinical relevance of the same kinases in the different cancer cohorts.

Additionally, we employed both a Spearman rank correlation and a linear regression analysis to determine the strength of the relationship between the two variables, CKI score and dependency score, for each cell line in the DepMap datasets. When considering TMOA kinases (cancer-specific kinases that are annotated as MOA targets for an approved drug), there exists a moderate to strong relationship ($p < 0.05$, $R < -0.6$) between CKI and dependency (Table S3). This suggests that targets with a low CKI score are likely to be not dependent in a particular cell line. Conversely, targets with a high CKI score are likely to be dependent. For example, in the UACC-893 breast cancer cell line, MOA target ERBB4 has a dependency score of only -0.18 and a corresponding CKI score of 3.65. However, MOA target PI3KCA has a dependency score of -1.8 and a CKI score of 14.38.

Finally, to evaluate the CKI as a predictive model for cellular dependency, receiver operating curves (ROC) were generated for cell lines from the DepMap datasets that showed significant differences in CKI scores between dependent and not-dependent kinases. A rank-ordered list of kinases (by CKI score) was generated per cancer type along with dependency scores for the corresponding cellular model. ROC curves were also generated using the average dependency score across all cell lines for a particular cancer model type. The dependency cutoff of < -1 was used to define the active (dependent) class. In breast cancer cell lines, CKI tended to be most predictive of dependency, as the ROC scores generated from the average dependency scores across all breast cancer cell lines in D2, Achilles, and Sanger datasets were 0.98, 0.78, and 0.76, respectively. Overall, we can conclude that the cellular dependency is “fair” to “excellent” at predicting clinical relevance as defined by the CKI (Table S3).

Understudied Kinases Are Highly Overexpressed across Multiple Cancer Types

The NIH IDG project research consortium (https://druggablegenome.net/) has curated a list of “understudied kinases,” which was constructed using a combination of bibliometric and other measures including lack of R01 funding, limited GeneRIF and Gene Ontology (GO) annotation, and lack of available potent and specific chemical probes. These include kinases that bear the TDLs of Tchem, Tbio, and Tdark. Despite being largely ignored by the scientific community, understudied kinases are gaining more attention due to their novelty and therefore opportunities and potential clinical importance.[15,16] Of the 151 understudied kinases, 22% are from the “other” group (which includes the subfamilies of *BUB*, *AUR*, and *PLK*), 20.7% are of the CGMC group (which contains MAP kinases and CDKs), and 17.3% are CaMKs. The rest of the understudied kinases include AGC and atypical kinases, with the lowest number of kinases belonging to the well-studied TK and tyrosine kinase-like (TKL) groups (Figures 2A and 2B).

Since DGE analysis is one of the most effective, scalable, and predictive methods for target prioritization,[38,39] we proceeded with a comprehensive analysis of the expression patterns of each of the 624 kinases across 20 TCGA cancer types (see STAR Methods for inclusion criteria). In total, 424 of the 624 kinases studied were found to be significantly differentially overexpressed (adjusted p value < 0.05) in at least two cancers in the TCGA (Figure 6A; Table S4). *BUB1*, *BUB1B*, and *PLK1* are all significantly overexpressed in every solid cancer analyzed in this study. In addition to being commonly differentially overexpressed, the average $\log_2$FC between normal and tumor cells

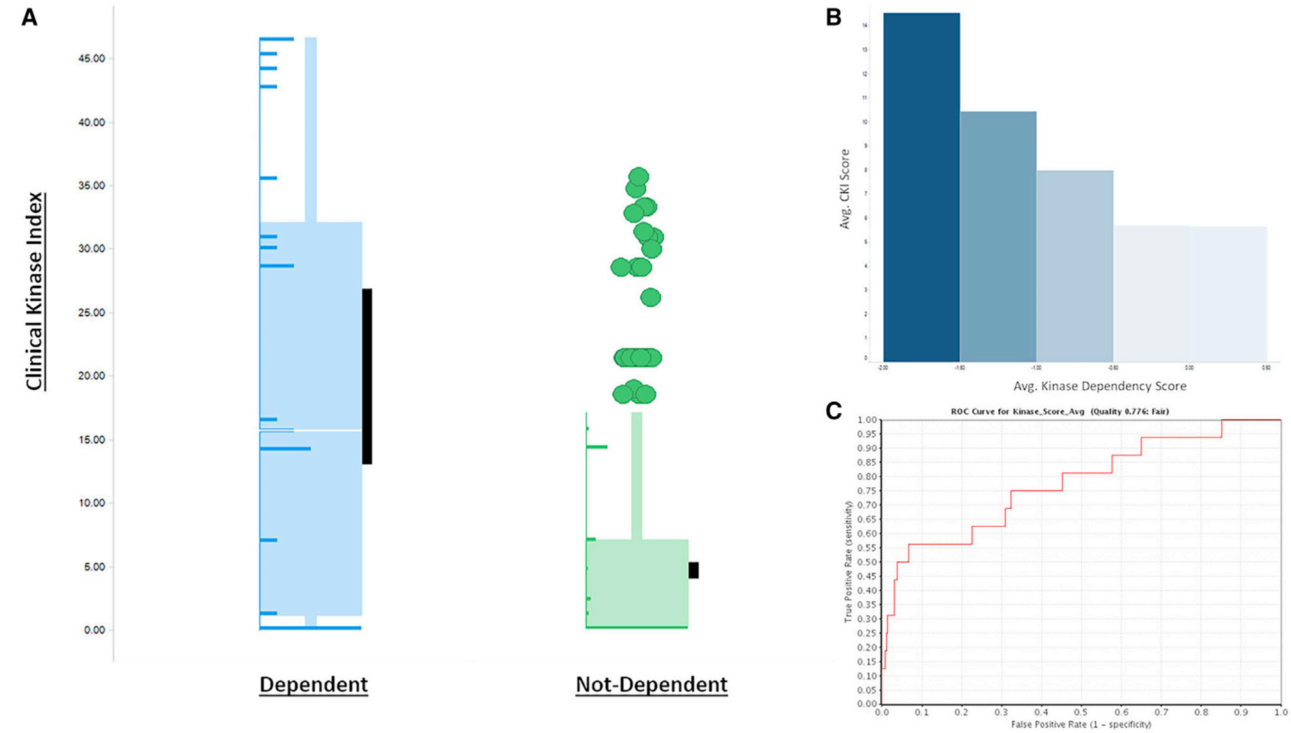

Figure 5. Comparison of DepMap Scores and CKI

(A) Kinases from the ACH-000851 cell line (lung cancer) were divided into "dependent" (DepMap Score < −1.0) and "not-dependent" groups. LUSC CKI scores were compared between the groups using the Kruskall-Wallis test. Kinases that are dependent in lung cancer cell lines also score significantly higher in the CKI ($p = 2.13E{-7}$). Black bars represent the 95% confidence interval of the mean. Distribution of scores is depicted as horizontal bar graphs superimposed on the boxplot.

(B) As DepMap scores (binned) increase (becoming less dependent), CKI scores decrease.

(C) ROC curve data using average CKI score and average dependency score with a cutoff of < −1.0 shows that DepMap data may be a predictive model of clinical relevance (ROC = 0.776).

is >2 for these kinases. Interestingly, these three kinases all interact with one another in the kinetochore-microtubule spindle assembly checkpoint during mitosis.[40,41] Several other kinases involved in mitosis/cell cycle are overexpressed in the majority of cancers (*AURKA*, *AURKB*, *CDK1*),[42–44] underscoring a hallmark of cancer, cell-cycle dysregulation. Many campaigns are well underway in the pre-clinical and early clinical trial phases to assess the efficacy of targeting such cell-cycle kinases, some with great potential.[45,46]

The DGE analysis also included 149 understudied kinases across 20 cancers from the TCGA. In total, 102 understudied kinases were shown to be differentially overexpressed in at least two cancers (Figure S1A). In every cancer cohort, the ratio of overexpressed understudied kinases compared to well-studied kinases was nearly 1:1. Several kinase groups appear to be enriched in their overexpression in various cancers. Averaging the $\log_2$FC of all kinases across all cancers demonstrates that certain kinase transcripts are consistently very highly upregulated. Kinases from the historically understudied CaMK group have the highest average $\log_2$FC (2.14) (Figure S1B). Twenty-four of the 26 understudied CaMKs are overexpressed in at least one cancer. The most commonly overexpressed CaMK is the pseudokinase *CAMKV*, which is significantly upregulated in 14 cancer cohorts. The understudied multi-functional CaMKs (*CAMK1D*, *CAMK1G*, *CAMKK1*, *PNCK*) are overexpressed in 12 cancers, with *PNCK* being overexpressed in 9 alone. *PNCK* (Tbio) mRNA expression and activity has recently been linked to renal cell carcinoma progression and survival,[17] breast cancer tumor microenvironment remodeling,[47] and decreased sensitivity to chemotherapies such as temozolomide.[48] *PNCK* is highly overexpressed in KIRC (>5 $\log_2$FC), LUSC (>6 $\log_2$FC), and LIHC (>6 $\log_2$FC). In fact, *PNCK* is the most significantly overexpressed kinase in these cancers, suggestive of a tumor-specific differential need for *PNCK* or CaMK activity compared to normal tissue. GTex and other human proteomic/transcriptomic studies show *PNCK* has very low expression levels in normal adult tissue, with the highest expression of mRNA and protein found in the dentate gyrus of the hippocampus. Non-specific CaMK inhibitor KN-93 has been shown in pre-clinical cancer cell models to induce cell-cycle arrest and apoptosis.[49,50] Despite these data, none of the CaMKs are currently targeted by FDA drugs, nor are they being evaluated in clinical or (published) pre-clinical studies.

Mutational Hotspots Found in Several Understudied Kinases May Correlate with Overall Survival

Many studies have demonstrated that somatically acquired mutations in kinase domains lead to tumorigenesis and promote

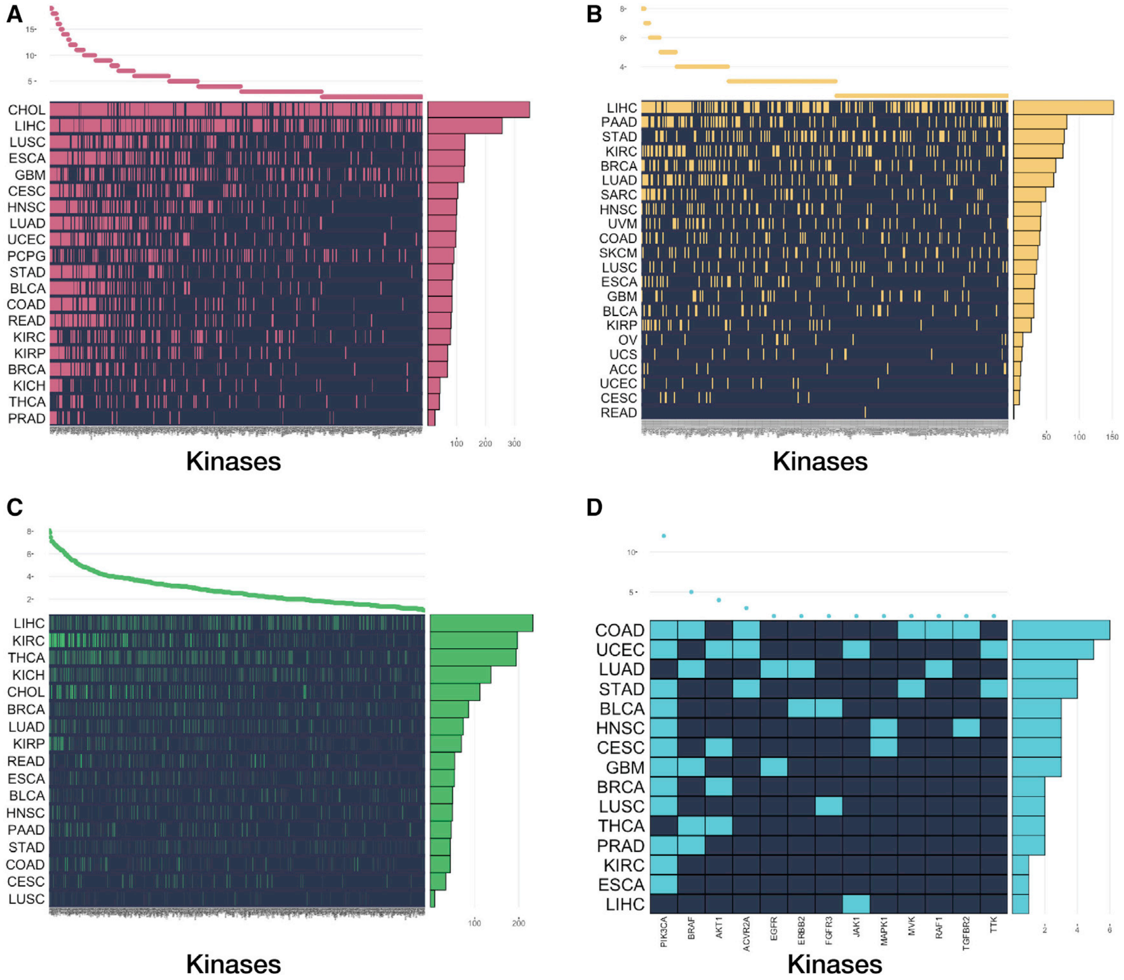

Figure 6. Four Analyses That Contributed to the CKI

The x axis contains all kinase names in the analysis, while the y axis represents the TCGA cancer. The plot on the top of the graph shows how many cancers a particular kinase is "significant" in. The plot on the right side of the graph represents how many significant kinases a particular cancer has for this analysis.

(A) 424 kinases are differentially overexpressed in at least two cancers.

(B) 317 kinases are prognostic of survival in at least two cancers.

(C) Many kinases have expressions that correlate with T, N, and M staging. This heat-map quantifies the extent to which each kinase correlates with the clinical outcome, TNM staging (total score out of 3, max score of 1 per parameter)

(D) 13 kinases have significant hotspot mutations in at least two cancers. See also Table S7.

cancer progression.[27,51,52] As mutations accumulate in a precancerous cell, some mutations confer selective advantage through the promotion of tumorigenic functions (such as unchecked cell-cycle progression, immune evasion, invasion, and metastasis), whereas others are effectively neutral "passengers" and are the byproduct of "driver" mutations. The discovery of frequent mutations in various kinase active sites has given rise to a new approach in drug development. Selectively targeting the mutated version of the kinase (versus the wild-type version) has led to great clinical success in oncology. For example, *BRAF* V600E inhibitors have greatly improved survival outcomes in melanoma patients with said mutation.[51] Thus, it is important to prioritize kinases with driver mutations as potential novel drug targets. Many cancer genes form mutational hotspots that disrupt their functional domains or active sites, leading to gain- or loss-of-function.[53] Therefore, we performed a mutational hotspot analysis on the entire kinome across 20 cancer types for which there were genetic data available. A hotspot mutation is defined as a mutation that occurs in a set of tumor samples significantly more frequently than what is observed by background mutations characterized by genes, cancer types, mutation types, and sequence contexts.[54] Hotspot mutations also affect an amino acid position and include missense, nonsense, insertion, and deletion mutations. In total, 42 kinases were found to have significant hotspot mutations, with kinases from the TK family being mutated most frequently. The most commonly mutated kinases were *PIK3CA* (12 cancers), *BRAF* (5 cancers), and *AKT1* (4 cancers), all of which have mutant-targeted inhibitors in clinic or in trial (Figure 6D; Table S5).[51,55,56] Eight understudied kinases were found to

have hotspot mutations in at least one cancer (*CDC42BPA*, *DYRK1B*, *DYRK4*, *LMTK3*, *MAPK15*, *NEK7*, *TSSK1B*, and *TTBK1*), with *DYRK4*, *LMTK3*, *MAPK15*, and *NEK7* all having significant hotspot mutations in STAD. Additional study showed that *DYRK1B* was mutated in 4.3% of endometrial carcinoma (UCEC) samples, with significantly worse overall survival for patients harboring the mutated kinase ($p < 0.05$; Figure S1C). Further work must be done to determine which of these kinase mutations are driver mutations and what downstream genomic and transcriptomic effects these mutations have on tumor progression. STAD and COAD were the cancers with the highest kinase mutational burden, with 16 and 11 kinases significantly mutated, respectively. There are currently no first-line treatments for gastric adenocarcinoma that include kinase inhibitors. As a highly heterogeneous disease, genomic data obtained from studies such as this may usher in a new era of personalized medicine for gastric cancer with novel kinase inhibitors against clinically relevant but rarely amplified and mutated kinases.

Comparing our mutational analysis to other pan-cancer TCGA analysis confirms that few kinases are significantly mutated, and few mutations are prognostic of survival. Smith and Sheltzer[57] identified *all* non-silent mutations with >2% frequency and used Cox-proportional hazard analysis to detect genes prognostic of survival. Analysis of their hazard ratios as *Z*-scores does show that Tdark kinase *NRK* is significantly mutated and prognostic in HNSC. Additionally, understudied kinases *CAMK1D*, *PNCK*, and *NEK3* have mutations with significant pan-cancer prognostic value, with *PNCK* being mutated frequently in LUAD and *NEK3* in COAD and READ ($p < 0.05$; Table S5). Various large-scale mutational analyses of tumors all confirm that the vast majority of somatically acquired mutations are passenger mutations of little or no functional consequence that arise simply as a result of the random mutagenic processes underlying the development of cancer.[58] It is rare, then, that a single kinase is commonly mutated (with the well-known exception, BRAF, which is mutated in over 60% of melanoma cases),[58] suggesting that several infrequently mutated kinases most likely contribute to tumorigenesis and progression.

Copy-Number Alterations and Gene Amplifications Are Frequent among Dark Kinases

There are 31 "dark kinases" (Tdark), about which very little information is known. Specifically, these kinases have fewer than 50 antibodies in antibodypedia, fewer than 3 gene RIFs, and a Jensen Lab PubMed text mining score of less than 5.[12] Tdark kinases are further characterized by poorly defined roles in wider signaling networks, poorly defined function and regulation, poorly defined kinase substrates, lack of activation-loop phospho-antibodies or immunohistochemistry-grade antibodies, and lack of selective chemical tools for functional characterization.[11] Over a quarter of the dark kinases belong to the "other" group of kinases. Dark kinases are also highly represented among the AGC group (13.3%), atypical kinases (10%), and non-protein "small-molecule kinases" (10%). A number of kinases from the so called "ignorome" *are*, in fact, known to interact with FDA-approved multi-kinase inhibitors. For example, according to DrugCentral data,[1,3] crizotinib, ruxolitinib, nintedanib, vandetanib, bosutinib, sorafenib, and sunitinib all inhibit understudied kinases in the low nanomolar range (including *SBK3*, *STK32A*, *RIOK1*, *CDK15*, and *CSNK1A1L*), one of which is Tdark (*CSNK1A1L*).[1] Thus, it is possible that some therapeutic effect or anti-cancer phenotype can be achieved through inhibition of dark kinases. Homology of kinase ATP binding sites across the kinome and the tendency of many kinase inhibitor chemotypes to inhibit multiple kinases strongly suggests that Tdark kinases can be effectively targeted with small-molecule inhibitors. To promote the pre-clinical study of these dark targets, we must first evaluate and analyze the available clinical data to prioritize the kinases based on several criteria including mutational status, frequency of genetic alteration, and DGE compared to normal samples. If these genomic or transcriptomic variations then correlate with clinical or pathological outcomes, the kinases could be explored in depth as potential oncogenic drivers.

In total, 22 of the dark kinases are significantly overexpressed in at least one TCGA cancer cohort. Increased expression of 15 Tdark kinases correlates with decreased overall survival across multiple cancers (Table S6). For example, high *ADCK5* mRNA levels are a negative prognostic indicator in KIRC, liver hepatocellular carcinoma (LIHC), UCEC, and uveal melanoma (UVM). In breast cancer (BRCA), overexpression of six dark kinases is associated with decreased overall survival (*ALPK3*, *CSNK1A1L*, *CSNK2A3*, *NRK*, *POMK*, and *PSKH1*) (Tables S2 and S3). We have also found that many Tdark kinases have altered genetics in the TCGA dataset. Although no Tdark kinases were detected to have significant hotspot mutations, by querying all dark kinases across 9,519 samples in the TCGA (for which copy number alterations [CNA]/copy number variants [CNV] data are available) in 24 cancer cohorts, we found that these genes have significantly altered copy numbers in 36% of patients. *NRBP2*, *POMK*, *ADCK5*, *SCYL3*, *PSKH2*, and *ETNK2* are amplified in over 5%–10% of all patients (Table S5). While CNA and CNVs do not linearly correlate with increases or decreases in mRNA expression, the potential increased expression of many kinases in primary human tumors and their location in focal amplification peaks with other cancer promoting genetic alterations suggests that dark kinases have important functions for the tumor cell phenotype that have not been characterized to date.

It was expected that Tclin kinases would generally score higher on our CKI than other less-characterized proteins. Kinase targets for which there are approved clinical drugs would be assumed to have disease-modifying properties in each cancer, such as effects on survival and tumor progression and size. In BLCA, CHOL, HSNC, KIRC, LUAD, READ, and STAD cohorts, CKI scores were highest for Tclin and MOA-cancer specific targets (TMOA). While this is generally the case for MOA targets (see above), in several cancer cohorts (BRCA, PRAD, COAD, LUSC), Tdark kinases scored higher on the CKI scale than known clinical targets (Tclin) (Tables S2 and S7). This suggests that these dark kinases may have similar phenotypic effects on cancer, comparable to well-known targets of approved drugs. Additional analysis of the dark kinome in these cohorts was performed to further explore clinical relevance. Out of 1,108 breast cancer patients, for example, dark kinases are altered in 54% of samples. Overall survival is significantly worse in patients with

dark kinase alterations than in those without (p = 4.90e−3). Multiple kinases are commonly amplified including *ADCK5* (14% of samples), *ETNK2* (13%), *NRBP2* (15%), *PSKH2* (12%), *RPS6KC1* (11%), and *SCYL3* (10%). Gene amplification of UCKL1 (6%) and SBK2 (3%) correlated negatively with overall survival and progression-free survival in a KM univariate analysis ($p < 0.05$; Table S5). Cox-proportional hazard ratios on Tdark kinases and their CNAs reveal that *SBK2*, *ETNK2*, *PSKH2*, *SCYL3*, and *NRBP2* are all significantly prognostic for survival across all cancers.[57] Genes that are amplified in a peak are likely involved in the driving of oncogenic pathways and alterations. *ETNK2* is significantly focally amplified in breast cancer tumors (Q = 7.6e−5) and located in a peak with 94 other genes.[59] *ADCK5*, *ETNK2*, *PSKH2*, *RPS6KC1*, and *SCYL3* are all significantly focally amplified in breast cancer samples as well but are not located in a peak region. Similar trends were confirmed in prostate, lung, and colon cancers (Table S5).

CaMKs Are Potential Prognostic Biomarkers

The identification and prioritization of novel biomarkers in cancer can be achieved by the integration of gene expression data with clinical data of patient samples. Genes that are prognostic for various cancers typically have expressions that correlate with overall survival (OS), progression-free survival (PFS), and tumor progression (TNM staging). Despite the abundance of studies exploring the prognostic value of many genes and kinases, very few prognostic biomarkers exist in clinical practice.[60] FDA-approved prognostic biomarkers typically include RNA expression panels of multiple genes. It is often true that good prognostic biomarkers may also be drug target candidates if functional characterization of the gene proves as such.[60] To assess the potential of utilizing kinase expression as prognostic biomarkers, we aggregated for each kinase, RNA sequencing (RNA-seq), and clinical data from 17 TCGA cancer types and performed KM survival analysis with logrank tests and ANOVA tests between the different tumor stages and grades. Kinases were scored separately for significance in each clinical parameter (TNM staging and histological grade when available; see Method Details).

In our analysis, 357 kinases were shown to be prognostic of M-stage, 522 for N-stage, and 552 for T-stage. In total, 24 of 31 dark kinases showed correlation with TNM staging in at least one cancer (Figure 6C). The average clinical score per kinase phylogenic group showed significant differences in each cancer cohort (pairwise t test, $p < 0.05$). The kinases with the highest average clinical scores include *NEK2*, *TRIB3*, *MELK*, *EPHA2*, *SPEG*, and *BRSK1*, several of which are CaMKs. *TRIB3* (Tbio), for example, shows correlation with metastasis in five cancer cohorts. Literature and other studies confirm that this kinase, a member of the CaMK family, may play a role in promoting metastasis in lung and colorectal cancers via induction by the transcription factor NF-kappaB.[61–63] CaMK members *CHEK2*, *TRIB2*, *STK17A*, and *STK17B* are also shown to correlate with TNM staging, further highlighting the potential clinical use for CaMKs as novel cancer targets (Table S7). In total, 317 out of 624 kinases were shown to correlate with survival in at least two cancers (Figure 6B; Table S6). The kinases with the highest survival scores have been well described in the literature as predictive and prognostic biomarkers in multiple cancers, notably *PGK1*, *PLK1*, and *AURKA*.[60,64,65] Less-studied kinases such as *ALPK3* (Tdark) (Figure S1D) and *SPEG* (Tbio) also were shown to correlate with survival in six cancer cohorts (Table S6).

DISCUSSION

The typical 20-year-long, multi-billion-dollar drug discovery pipeline all begins with target identification and prioritization.[66] This is arguably one of the most important steps, as drug failure in the clinic is mostly due to a lack of efficacy or due to toxicities from poor target choice.[66,67] The use of large-scale omics data has streamlined this process by allowing researchers to combine multiple parameters to evaluate a protein's potential as a drug target or biomarker. Often, the first glimmer of target potential arises from the analysis of RNA and protein expression in disease tissue compared to healthy tissue. TCGA is one such publicly available database where a breadth of information (transcriptomic, genomic, proteomic, clinical, pathological, and histological) is available for this investigation. An ideal drug target, as described by Bayer,[68] is one that is first and foremost "druggable" and "assayable"; this is the case for most kinases due to the presence of both a well-defined pocket and ATPase activity. This target will also have an activity that is disease specific (i.e., it is differentially expressed and active in diseased tissue compared to normal tissue). This can be determined preliminarily by DGE analysis and proteomic or phosphoproteomic studies. The target should not be uniformly distributed and expressed throughout the body, a characteristic that can be checked using expression databases such as GTex[69] or The Human Protein Atlas.[70] Also, immunoprecipitation (IP) is a critical consideration. Therefore, new targets can be attractive, such as "dark" and understudied kinases where there are few or no known small-molecule inhibitors. Finally, and the most difficult parameter to satisfy using informatics alone, kinase targets have to be disease modifying or have a proven function in the pathophysiology of disease. While dysregulation of the kinome as a whole has been indicated in the initiation and progression of nearly every cancer type,[71] disease-modifying kinases must be identified per cancer type using a combination of genetic perturbations and biochemical analyses. Our various analyses, including correlation of CKI with MOA targets clinical studies and cross-validating CKI with experimental datasets such as DepMap,[72] demonstrate that CKI can prioritize drug targets for "disease-modifying" potential, before further target validation studies are pursued. In totality, combining multiple large-scale multi-omics datasets can be a useful first step in prioritizing novel kinase target lists prior to conducting any additional cell-based or animal-based experiments.

In this study, we systematically integrated DGE, KM survival, and mutational hotspot and clinical/pathological correlation analyses in order to prioritize clinically relevant kinase targets across 17 TCGA cancer types. Our clinically focused pan-cancer and pan-kinome analysis highlighted multiple understudied kinases with the potential of promising druggable target opportunities. Moreover, through the development of our accompanying CKI app, which is freely available at http://cki.ccs.miami.

edu/, we are facilitating the discovery, exploration, and analysis of our data by the scientific community. Our plethora of rich metadata annotations (e.g., TDL, approved MOA, clinical trial status, and kinase phylogenetic and functional classifications) offers researchers a quick and intuitive way to explore the entire kinome and identify understudied kinases with a high therapeutic potential. This app allows a comprehensive view of the kinome based on cancer-type-specific differential expression, survival data, and TNM pathological staging. Researchers can obtain a prioritized list of dark and understudied kinases based on multiple criteria (e.g., cancer type, kinase family). This capability can drive a more efficient drug target prioritization by the research communities. For each dark or understudied kinase, researchers can obtain cancer-type-specific analysis and rank-ordered prioritizations. As more information is discovered and more bioinformatics tools and workflows are available, such as phosphoproteomics and active kinome profiling, the CKI will be updated and optimized to continually enrich the dataset for clinically relevant kinase targets.

All scoring, classifications, normalized data, and statistical analyses are available via the CKI app. To use the app, one can simply select the "Gene" tab and choose a kinase of interest. The CKI scores for each cancer for this kinase will be generated in table form, along with TDL, rank, kinase group and family, and whether or not this kinase has an approved drug MOA. Many annotations from the Drug Target Ontology (DTO)[18] are available as facets to filter and select kinase targets. To start from a disease of interest, one can select a TCGA cancer and the kinase of interest in the "Disease" tab. A kinase table is generated with the specific TCGA cancer and kinase sorted by CKI score. Next, in a sub-tab, a volcano plot will be generated where all differentially expressed genes are displayed. One may click points on the plot to see the specific gene, its count-per-million, and logFC (compared to normal tissue). If the "Study" sub-tab is selected, cancer and gene may be chosen to display boxplots representing mRNA levels for each T, N, and M stage. Finally, if the "Survival" sub-tab is selected, a KM plot is generated for the cancer and gene pair of interest. All data tables may be downloaded via the "Download Data" tab.

Comparing the average CKI scores of "understudied" versus "studied" kinases across all cancers and within individual cancer cohorts suggests that there are many understudied kinases with clinical relevance comparable to currently approved kinase drug targets. The highest-scoring understudied kinase is PKMYT1. PKMYT1 is a member of the WEE1 family of kinases that negatively regulates the G2/M transition of the cell cycle by phosphorylating and inactivating CDK1.[73] *PKMYT1* is overexpressed in 17 of the 20 cancers analyzed in this study. Cox-regression analysis of *PKMYT1* reveals this kinase is a powerful prognostic and predictive biomarker for survival in KIRC and KIRP cohorts. Other computational work has identified *PKMYT1* as a novel drug target for kidney cancer, using co-expression analysis to reveal *PKMYT1* clusters with other important cell-cycle genes.[74] *PKMYT1* is classified as Tchem with several active compounds, including PD-0166285, IC_{50} = 7 nM and PD-173955, Kd = 44 nM. Although these compounds are not considered chemical probes, they provide starting points for the development of a selective probe or a viable lead compound.

While many of the high-scoring novel kinases are also beginning to be discussed in other studies, many are too "dark" and thus warrant more exploration. Even in large-scale datasets and studies, dark kinases are excluded from the analyses due to a lack of validated antibodies, assays, and chemical probes and a general lack of interest and knowledge of the biology of these targets. A large-scale concerted effort must be taken to effectively bring these "dark" kinases into the light. Such target validation efforts must include RNAi/CRISPR knockdown/knockout cell lines and mice models, elucidation of co-expression and regulatory networks, substrate identification and assay development, development of highly specific chemical probes, and determination of co-crystal structures to facilitate the optimization of lead compounds for future drug development efforts. Much of this work is currently pursued in the IDG project (https://druggablegenome.net/). The results of this paper will help us, and other groups, select and prioritize their target of choice based on the clinical and biological focus of their own research interests. 2018 was a record year for FDA approvals, yet only 3 of the 39 drugs targeted novel kinase targets and moved TDLs from Tchem to Tclin.[2] The rate at which kinase targets move from Tbio or Tdark (little known biology and no potent chemical probes) to Tchem (a small-molecule probe with Kd < 30 nm) is alarmingly slow. No more than 20 IDG targets move from Tbio to Tchem each year, a number encompassing *all* target types (kinases, GPCRs, ion channels). By this pace, it would take decades to wholly illuminate the kinome. Thus, the biases against understudied targets must be lifted, particularly in grant funding, so that there can a full-fledged concerted effort to explore the druggable genome.

Limitations of Study

The CKI is a tool for researchers to use to aid the target prioritization or identification process. All data used to generate the index and annotate the kinases have come from large-scale, validated datasets. Target validation, however, requires multifactorial experiments. Here, we offer a first step in the process and seek to encourage others to follow up in their cellular or animal model of choice to further evaluate understudied kinases as novel drug targets.

STAR★METHODS

Detailed methods are provided in the online version of this paper and include the following:

- KEY RESOURCES TABLE
- RESOURCE AVAILABILITY
  - Lead Contact
  - Materials Availability
  - Data and Code Availability
- METHOD DETAILS
  - Inclusion Criteria: TCGA Datasets
  - Inclusion Criteria: Kinases
  - Data Extraction and Preprocessing
  - Clinical Data
  - Differential Gene Expression Analysis
  - Survival Analysis

○ Mutational Hotspot Analysis
○ Clinical Analysis
○ Scoring
○ Clinical Kinase Index (CKI) Web Application (https://schurerlab.shinyapps.io/CKIApp/)
○ DepMap Analysis
○ External Tools

- QUANTIFICATION AND STATISTICAL ANALYSIS
- ADDITIONAL RESOURCES

SUPPLEMENTAL INFORMATION

Supplemental Information can be found online at https://doi.org/10.1016/j.xcrm.2020.100128.

ACKNOWLEDGMENTS

This work was supported by NIH grants U54HL127624 (BD2K LINCS Data Coordination and Integration Center, DCIC), U24TR002278 (Illuminating the Druggable Genome Resource Dissemination and Outreach Center, IDG-RDOC), U01LM012630 (BD2K, Enhancing the efficiency and effectiveness of digital curation for biomedical 'big data'), and P30CA240139 (NCI Sylvester Cancer Center Support Grant), and the State of Florida Biomedical Research Program, Bankhead Coley grant 9BC13.

AUTHOR CONTRIBUTIONS

D.E., V.S., and S.C.S. conceived the project, and R.K. carried out statistical analyses. D.E. and S.C.S. analyzed and interpreted data, and R.K. developed the web app. D.E. wrote the manuscript, and D.E., V.S., and R.K. made figures.

DECLARATION OF INTERESTS

The authors declare no competing interests.

Received: October 29, 2019
Revised: April 25, 2020
Accepted: September 24, 2020
Published: October 20, 2020

REFERENCES

1. Ursu, O., Holmes, J., Knockel, J., Bologa, C.G., Yang, J.J., Mathias, S.L., Nelson, S.J., and Oprea, T.I. (2017). DrugCentral: online drug compendium. Nucleic Acids Res. *45*, D932–D939.
2. Ursu, O., Glick, M., and Oprea, T. (2019). Novel drug targets in 2018. Nat. Rev. Drug Discov. Published online March 26, 2019. https://doi.org/10.1038/d41573-019-00052-5.
3. Ursu, O., Holmes, J., Bologa, C.G., Yang, J.J., Mathias, S.L., Stathias, V., Nguyen, D.T., Schürer, S., and Oprea, T. (2019). DrugCentral 2018: an update. Nucleic Acids Res. *47*, D963–D970.
4. Wallerand, H., Robert, G., Bernhard, J.C., Ravaud, A., and Patard, J.J. (2010). Tyrosine-kinase inhibitors in the treatment of muscle invasive bladder cancer and hormone refractory prostate cancer. Arch. Esp. Urol. *63*, 773–787.
5. Vincenzi, B., Imperatori, M., Silletta, M., Marrucci, E., Santini, D., and Tonini, G. (2015). Emerging kinase inhibitors of the treatment of gastric cancer. Expert Opin. Emerg. Drugs *20*, 479–493.
6. Petrylak, D.P., Tangen, C.M., Van Veldhuizen, P.J., Jr., Goodwin, J.W., Twardowski, P.W., Atkins, J.N., Kakhil, S.R., Lange, M.K., Mansukhani, M., and Crawford, E.D. (2010). Results of the Southwest Oncology Group phase II evaluation (study S0031) of ZD1839 for advanced transitional cell carcinoma of the urothelium. BJU Int. *105*, 317–321.
7. Morishita, A., Gong, J., and Masaki, T. (2014). Targeting receptor tyrosine kinases in gastric cancer. World J. Gastroenterol. *20*, 4536–4545.
8. Westphal, M., Maire, C.L., and Lamszus, K. (2017). EGFR as a Target for Glioblastoma Treatment: An Unfulfilled Promise. CNS Drugs *31*, 723–735.
9. Kruck, S., Bedke, J., Hennenlotter, J., Ohneseit, P.A., Kuehs, U., Senger, E., Sievert, K.D., and Stenzl, A. (2010). Activation of mTOR in renal cell carcinoma is due to increased phosphorylation rather than protein overexpression. Oncol. Rep. *23*, 159–163.
10. Oprea, T.I., Bologa, C.G., Brunak, S., Campbell, A., Gan, G.N., Gaulton, A., Gomez, S.M., Guha, R., Hersey, A., Holmes, J., et al. (2018). Unexplored therapeutic opportunities in the human genome. Nat. Rev. Drug Discov. *17*, 377.
11. Nguyen, D.T., Mathias, S., Bologa, C., Brunak, S., Fernandez, N., Gaulton, A., Hersey, A., Holmes, J., Jensen, L.J., Karlsson, A., et al. (2017). Pharos: Collating protein information to shed light on the druggable genome. Nucleic Acids Res. *45*, D995–D1002.
12. Wishart, D.S., Knox, C., Guo, A.C., Shrivastava, S., Hassanali, M., Stothard, P., Chang, Z., and Woolsey, J. (2006). DrugBank: a comprehensive resource for in silico drug discovery and exploration. Nucleic Acids Res. *34*, D668–D672.
13. Carles, F., Bourg, S., Meyer, C., and Bonnet, P. (2018). PKIDB: A Curated, Annotated and Updated Database of Protein Kinase Inhibitors in Clinical Trials. Molecules *23*, E908.
14. Axtman, A., Drewry, D., and Wells, C. (2019). CDK16: the pick of the understudied PCTAIRE kinases. Nat. Rev. Drug Discov. *18*, 489.
15. Asquith, C.R.M., East, M.P., and Zuercher, W.J. (2019). RIOK2: straddling the kinase/ATPase line. Nat. Rev. Drug Discov. Published online July 1, 2019. https://doi.org/10.1038/d41573-019-00107-7.
16. Belinky, F., Nativ, N., Stelzer, G., Zimmerman, S., Iny Stein, T., Safran, M., and Lancet, D. (2015). PathCards: multi-source consolidation of human biological pathways. Database (Oxford) *2015*, bav006.
17. Lin, Y., Mehta, S., Küçük-McGinty, H., Turner, J.P., Vidovic, D., Forlin, M., Koleti, A., Nguyen, D.T., Jensen, L.J., Guha, R., et al. (2017). Drug target ontology to classify and integrate drug discovery data. J. Biomed. Semantics *8*, 50.
18. Stathias, V., Jermakowicz, A.M., Maloof, M.E., Forlin, M., Walters, W., Suter, R.K., Durante, M.A., Williams, S.L., Harbour, J.W., Volmar, C.H., et al. (2018). Drug and disease signature integration identifies synergistic combinations in glioblastoma. Nat. Commun. *9*, 5315.
19. Cheng, F., Kovács, I.A., and Barabási, A.L. (2019). Publisher Correction: Network-based prediction of drug combinations. Nat. Commun. *10*, 1806.
20. Huang, C.T., Hsieh, C.H., Chung, Y.H., Oyang, Y.J., Huang, H.C., and Juan, H.F. (2019). Perturbational Gene-Expression Signatures for Combinatorial Drug Discovery. iScience *15*, 291–306.
21. Nussinov, R., Tsai, C.J., and Jang, H. (2017). A New View of Pathway-Driven Drug Resistance in Tumor Proliferation. Trends Pharmacol. Sci. *38*, 427–437.
22. Stuhlmiller, T.J., Miller, S.M., Zawistowski, J.S., Nakamura, K., Beltran, A.S., Duncan, J.S., Angus, S.P., Collins, K.A., Granger, D.A., Reuther, R.A., et al. (2015). Inhibition of Lapatinib-Induced Kinome Reprogramming in ERBB2-Positive Breast Cancer by Targeting BET Family Bromodomains. Cell Rep. *11*, 390–404.
23. Regan Anderson, T.M., Peacock, D.L., Daniel, A.R., Hubbard, G.K., Lofgren, K.A., Girard, B.J., Schörg, A., Hoogewijs, D., Wenger, R.H., Seagroves, T.N., and Lange, C.A. (2013). Breast tumor kinase (Brk/PTK6) is a mediator of hypoxia-associated breast cancer progression. Cancer Res. *73*, 5810–5820.
24. Xiang, B., Chatti, K., Qiu, H., Lakshmi, B., Krasnitz, A., Hicks, J., Yu, M., Miller, W.T., and Muthuswamy, S.K. (2008). Brk is coamplified with ErbB2 to promote proliferation in breast cancer. Proc. Natl. Acad. Sci. USA *105*, 12463–12468.

25. Harvey, A.J., and Crompton, M.R. (2003). Use of RNA interference to validate Brk as a novel therapeutic target in breast cancer: Brk promotes breast carcinoma cell proliferation. Oncogene *22*, 5006–5010.

26. Paez, J.G., Jänne, P.A., Lee, J.C., Tracy, S., Greulich, H., Gabriel, S., Herman, P., Kaye, F.J., Lindeman, N., Boggon, T.J., et al. (2004). EGFR mutations in lung cancer: correlation with clinical response to gefitinib therapy. Science *304*, 1497–1500.

27. Ladanyi, M., and Pao, W. (2008). Lung adenocarcinoma: guiding EGFR-targeted therapy and beyond. Mod. Pathol. *21*, S16–S22.

28. Stinchcombe, T.E., and Johnson, G.L. (2014). MEK inhibition in non-small cell lung cancer. Lung Cancer *86*, 121–125.

29. Kim, C., and Giaccone, G. (2018). MEK inhibitors under development for treatment of non-small-cell lung cancer. Expert Opin. Investig. Drugs *27*, 17–30.

30. Gutteridge, R.E., Ndiaye, M.A., Liu, X., and Ahmad, N. (2016). Plk1 Inhibitors in Cancer Therapy: From Laboratory to Clinics. Mol. Cancer Ther. *15*, 1427–1435.

31. Smith, L., Farzan, R., Ali, S., Buluwela, L., Saurin, A.T., and Meek, D.W. (2017). The responses of cancer cells to PLK1 inhibitors reveal a novel protective role for p53 in maintaining centrosome separation. Sci. Rep. *7*, 16115.

32. Oikawa, D., Kitamura, A., Kinjo, M., and Iwawaki, T. (2012). Direct association of unfolded proteins with mammalian ER stress sensor, IRE1β. PLoS ONE *7*, e51290.

33. Madden, E., Logue, S.E., Healy, S.J., Manie, S., and Samali, A. (2019). The role of the unfolded protein response in cancer progression: From oncogenesis to chemoresistance. Biol. Cell *111*, 1–17.

34. Liberzon, A., Subramanian, A., Pinchback, R., Thorvaldsdóttir, H., Tamayo, P., and Mesirov, J.P. (2011). Molecular signatures database (MSigDB) 3.0. Bioinformatics *27*, 1739–1740.

35. Liberzon, A., Birger, C., Thorvaldsdóttir, H., Ghandi, M., Mesirov, J.P., and Tamayo, P. (2015). The Molecular Signatures Database (MSigDB) hallmark gene set collection. Cell Syst. *1*, 417–425.

36. Tsherniak, A., Vazquez, F., Montgomery, P.G., Weir, B.A., Kryukov, G., Cowley, G.S., Gill, S., Harrington, W.F., Pantel, S., Krill-Burger, J.M., et al. (2017). Defining a Cancer Dependency Map. Cell *170*, 564–576.e16.

37. Iorio, F., Knijnenburg, T.A., Vis, D.J., Bignell, G.R., Menden, M.P., Schubert, M., Aben, N., Gonçalves, E., Barthorpe, S., Lightfoot, H., et al. (2016). A Landscape of Pharmacogenomic Interactions in Cancer. Cell *166*, 740–754.

38. Parca, L., Pepe, G., Pietrosanto, M., Galvan, G., Galli, L., Palmeri, A., Sciandrone, M., Ferrè, F., Ausiello, G., and Helmer-Citterich, M. (2019). Modeling cancer drug response through drug-specific informative genes. Sci. Rep. *9*, 15222.

39. Bolanos-Garcia, V.M., and Blundell, T.L. (2011). BUB1 and BUBR1: multifaceted kinases of the cell cycle. Trends Biochem. Sci. *36*, 141–150.

40. Izumi, H., Matsumoto, Y., Ikeuchi, T., Saya, H., Kajii, T., and Matsuura, S. (2009). BubR1 localizes to centrosomes and suppresses centrosome amplification via regulating Plk1 activity in interphase cells. Oncogene *28*, 2806–2820.

41. Keen, N., and Taylor, S. (2004). Aurora-kinase inhibitors as anticancer agents. Nat. Rev. Cancer *4*, 927–936.

42. Prevo, R., Pirovano, G., Puliyadi, R., Herbert, K.J., Rodriguez-Berriguete, G., O'Docherty, A., Greaves, W., McKenna, W.G., and Higgins, G.S. (2018). CDK1 inhibition sensitizes normal cells to DNA damage in a cell cycle dependent manner. Cell Cycle *17*, 1513–1523.

43. Otto, T., and Sicinski, P. (2017). Cell cycle proteins as promising targets in cancer therapy. Nat. Rev. Cancer *17*, 93–115.

44. Beltran, H., Oromendia, C., Danila, D.C., Montgomery, B., Hoimes, C., Szmulewitz, R.Z., Vaishampayan, U., Armstrong, A.J., Stein, M., Pinski, J., et al. (2019). A Phase II Trial of the Aurora Kinase A Inhibitor Alisertib for Patients with Castration-resistant and Neuroendocrine Prostate Cancer: Efficacy and Biomarkers. Clin. Cancer Res. *25*, 43–51.

45. Diamond, J.R., Eckhardt, S.G., Pitts, T.M., van Bokhoven, A., Aisner, D., Gustafson, D.L., Capasso, A., Sams, S., Kabos, P., Zolman, K., et al. (2018). A phase II clinical trial of the Aurora and angiogenic kinase inhibitor ENMD-2076 for previously treated, advanced, or metastatic triple-negative breast cancer. Breast Cancer Res. *20*, 82.

46. Sang, L.J., Ju, H.Q., Liu, G.P., Tian, T., Ma, G.L., Lu, Y.X., Liu, Z.X., Pan, R.L., Li, R.H., Piao, H.L., et al. (2018). LncRNA CamK-A Regulates Ca^{2+}-Signaling-Mediated Tumor Microenvironment Remodeling. Mol. Cell *72*, 601.

47. Owusu, M., Bannauer, P., Ferreira da Silva, J., Mourikis, T.P., Jones, A., Majek, P., Caldera, M., Wiedner, M., Lardeau, C.-H., Mueller, A.C., et al. (2019). Mapping the Human Kinome in Response to DNA Damage. Cell Rep. *26*, 555–563.e6.

48. Rokhlin, O.W., Guseva, N.V., Taghiyev, A.F., Glover, R.A., and Cohen, M.B. (2010). KN-93 inhibits androgen receptor activity and induces cell death irrespective of p53 and Akt status in prostate cancer. Cancer Biol. Ther. *9*, 224–235.

49. Rodriguez-Mora, O.G., LaHair, M.M., McCubrey, J.A., and Franklin, R.A. (2005). Calcium/calmodulin-dependent kinase I and calcium/calmodulin-dependent kinase kinase participate in the control of cell cycle progression in MCF-7 human breast cancer cells. Cancer Res. *65*, 5408–5416.

50. Rivalland, G., and Mitchell, P. (2016). Combined BRAF and MEK inhibition in BRAF-mutant NSCLC. Lancet Oncol. *17*, 860–862.

51. Torkamani, A., Verkhivker, G., and Schork, N.J. (2009). Cancer driver mutations in protein kinase genes. Cancer Lett. *281*, 117–127.

52. Mayakonda, A., Lin, D.C., Assenov, Y., Plass, C., and Koeffler, H.P. (2018). Maftools: efficient and comprehensive analysis of somatic variants in cancer. Genome Res. *28*, 1747–1756.

53. Chen, T., Wang, Z., Zhou, W., Chong, Z., Meric-Bernstam, F., Mills, G.B., and Chen, K. (2016). Hotspot mutations delineating diverse mutational signatures and biological utilities across cancer types. BMC Genomics *17*, 394.

54. Stirrups, R. (2019). Alpelisib plus fulvestrant for PIK3CA-mutated breast cancer. Lancet Oncol. *20*, e347.

55. Xing, Y., Lin, N.U., Maurer, M.A., Chen, H., Mahvash, A., Sahin, A., Akcakanat, A., Li, Y., Abramson, V., Litton, J., et al. (2019). Phase II trial of AKT inhibitor MK-2206 in patients with advanced breast cancer who have tumors with PIK3CA or AKT mutations, and/or PTEN loss/PTEN mutation. Breast Cancer Res. *21*, 78.

56. Smith, J.C., and Sheltzer, J.M. (2018). Systematic identification of mutations and copy number alterations associated with cancer patient prognosis. eLife *7*, e39217.

57. Vogelstein, B., Papadopoulos, N., Velculescu, V.E., Zhou, S., Diaz, L.A., Jr., and Kinzler, K.W. (2013). Cancer genome landscapes. Science *339*, 1546–1558.

58. Mermel, C.H., Schumacher, S.E., Hill, B., Meyerson, M.L., Beroukhim, R., and Getz, G. (2011). GISTIC2.0 facilitates sensitive and confident localization of the targets of focal somatic copy-number alteration in human cancers. Genome Biol. *12*, R41.

59. Goossens, N., Nakagawa, S., Sun, X., and Hoshida, Y. (2015). Cancer biomarker discovery and validation. Transl. Cancer Res. *4*, 256–269.

60. Hong, B., Zhou, J., Ma, K., Zhang, J., Xie, H., Zhang, K., Li, L., Cai, L., Zhang, N., Zhang, Z., and Gong, K. (2019). TRIB3 Promotes the Proliferation and Invasion of Renal Cell Carcinoma Cells via Activating MAPK Signaling Pathway. Int. J. Biol. Sci. *15*, 587–597.

61. Hua, F., Li, K., Yu, J.J., and Hu, Z.W. (2015). The TRIB3-SQSTM1 interaction mediates metabolic stress-promoted tumorigenesis and progression via suppressing autophagic and proteasomal degradation. Autophagy *11*, 1929–1931.

62. Wennemers, M., Bussink, J., Scheijen, B., Nagtegaal, I.D., van Laarhoven, H.W., Raleigh, J.A., Varia, M.A., Heuvel, J.J., Rouschop, K.M., Sweep, F.C., and Span, P.N. (2011). Tribbles homolog 3 denotes a poor prognosis

in breast cancer and is involved in hypoxia response. Breast Cancer Res. *13*, R82.

63. Lin, P., Xiong, D.D., Dang, Y.W., Yang, H., He, Y., Wen, D.Y., Qin, X.G., and Chen, G. (2017). The anticipating value of PLK1 for diagnosis, progress and prognosis and its prospective mechanism in gastric cancer: a comprehensive investigation based on high-throughput data and immunohistochemical validation. Oncotarget *8*, 92497–92521.
64. Mobley, A., Zhang, S., Bondaruk, J., Wang, Y., Majewski, T., Caraway, N.P., Huang, L., Shoshan, E., Velazquez-Torres, G., Nitti, G., et al. (2017). Aurora Kinase A is a Biomarker for Bladder Cancer Detection and Contributes to its Aggressive Behavior. Sci. Rep. *7*, 40714.
65. Hughes, J.P., Rees, S., Kalindjian, S.B., and Philpott, K.L. (2011). Principles of early drug discovery. Br. J. Pharmacol. *162*, 1239–1249.
66. Plenge, R.M., Scolnick, E.M., and Altshuler, D. (2013). Validating therapeutic targets through human genetics. Nat. Rev. Drug Discov. *12*, 581–594.
67. Gashaw, I., Ellinghaus, P., Sommer, A., and Asadullah, K. (2012). What makes a good drug target? Drug Discov. Today *17*, S24–S30.
68. Carithers, L.J., and Moore, H.M. (2015). The Genotype-Tissue Expression (GTEx) Project. Biopreserv. Biobank. *13*, 307–308.
69. Uhlén, M., Fagerberg, L., Hallström, B.M., Lindskog, C., Oksvold, P., Mardinoglu, A., Sivertsson, Å., Kampf, C., Sjöstedt, E., Asplund, A., et al. (2015). Proteomics. Tissue-based map of the human proteome. Science *347*, 1260419.
70. Du, Z., and Lovly, C.M. (2018). Mechanisms of receptor tyrosine kinase activation in cancer. Mol. Cancer *17*, 58.
71. Liu, Y., Beyer, A., and Aebersold, R. (2016). On the Dependency of Cellular Protein Levels on mRNA Abundance. Cell *165*, 535–550.
72. Schmidt, M., Rohe, A., Platzer, C., Najjar, A., Erdmann, F., and Sippl, W. (2017). Regulation of G2/M Transition by Inhibition of WEE1 and PKMYT1 Kinases. Molecules *22*, E2045.
73. Ma'ayan, A. (2019). PKMYT1 is a computationally predicted target for kidney cancer. bioRxiv. https://doi.org/10.1101/677245.
74. Santos, R., Ursu, O., Gaulton, A., Bento, A.P., Donadi, R.S., Bologa, C.G., Karlsson, A., Al-Lazikani, B., Hersey, A., Oprea, T.I., and Overington, J.P. (2017). A comprehensive map of molecular drug targets. Nat. Rev. Drug Discov. *16*, 19–34.
75. Markham, A. (2019). Erdafitinib: First Global Approval. Drugs *79*, 1017–1021.
76. Collado-Torres, L., Nellore, A., Kammers, K., Ellis, S.E., Taub, M.A., Hansen, K.D., Jaffe, A.E., Langmead, B., and Leek, J.T. (2017). Reproducible RNA-seq analysis using recount2. Nat. Biotechnol. *35*, 319–321.
77. Ritchie, M.E., Phipson, B., Wu, D., Hu, Y., Law, C.W., Shi, W., and Smyth, G.K. (2015). limma powers differential expression analyses for RNA-sequencing and microarray studies. Nucleic Acids Res. *43*, e47.
78. Amin, M.B., Greene, F.L., Edge, S.B., Compton, C.C., Gershenwald, J.E., Brookland, R.K., Meyer, L., Gress, D.M., Byrd, D.R., and Winchester, D.P. (2017). The Eighth Edition AJCC Cancer Staging Manual: Continuing to build a bridge from a population-based to a more "personalized" approach to cancer staging. CA Cancer J. Clin. *67*, 93–99.
79. Liu, J., Lichtenberg, T., Hoadley, K.A., Poisson, L.M., Lazar, A.J., Cherniack, A.D., Kovatich, A.J., Benz, C.C., Levine, D.A., Lee, A.V., et al. (2018). An Integrated TCGA Pan-Cancer Clinical Data Resource to Drive High-Quality Survival Outcome Analytics. Cell *173*, 400–416.e11.
80. Grossman, R.L., Heath, A.P., Ferretti, V., Varmus, H.E., Lowy, D.R., Kibbe, W.A., and Staudt, L.M. (2016). Toward a Shared Vision for Cancer Genomic Data. N. Engl. J. Med. *375*, 1109–1112.
81. Gentleman, R.C., Carey, V.J., Bates, D.M., Bolstad, B., Dettling, M., Dudoit, S., Ellis, B., Gautier, L., Ge, Y., Gentry, J., et al. (2004). Bioconductor: open software development for computational biology and bioinformatics. Genome Biol. *5*, R80.
82. Colaprico, A., Silva, T.C., Olsen, C., Garofano, L., Cava, C., Garolini, D., Sabedot, T.S., Malta, T.M., Pagnotta, S.M., Castiglioni, I., et al. (2016). TCGAbiolinks: an R/Bioconductor package for integrative analysis of TCGA data. Nucleic Acids Res. *44*, e71.
83. Therneau, T.M., and Grambsch, P.M. (2000). Modeling survival data: extending the Cox model (Springer).
84. Porta-Pardo, E., and Godzik, A. (2014). e-Driver: a novel method to identify protein regions driving cancer. Bioinformatics *30*, 3109–3114.
85. Cerami, E., Gao, J., Dogrusoz, U., Gross, B.E., Sumer, S.O., Aksoy, B.A., Jacobsen, A., Byrne, C.J., Heuer, M.L., Larsson, E., et al. (2012). The cBio cancer genomics portal: an open platform for exploring multidimensional cancer genomics data. Cancer Discov. *2*, 401–404.

STAR★METHODS

KEY RESOURCES TABLE

REAGENT or RESOURCE	SOURCE	IDENTIFIER
Software and Algorithms		
TCGA data portal	(Collins, 2007)	https://portal.gdc.cancer.gov/
TCGAbiolinks pipeline	(Colaprico et al., 2016)	https://bioconductor.org/packages/release/bioc/html/TCGAbiolinks.html
R/Bioconductor	R packages	https://www.bioconductor.org/
cBioportal for cancer genomics	(Cerami et al., 2012; Gao et al., 2013)	https://www.cbioportal.org/
FireHose Broad GDAC	Broad Institute of MIT & Harvard	https://gdac.broadinstitute.org/
recount2	(Collado-Torres et al., 2017)	http://www.nature.com/articles/nbt.3838
Pharos	(Nguyen et al., 2017)	PMID: 27903890

RESOURCE AVAILABILITY

Lead Contact

Further information and requests for resources should be directed to and will be fulfilled by the Lead Contact, Stephan Schürer (sschurer@med.miami.edu)

Materials Availability

This study did not generate new unique reagents.

Data and Code Availability

The published article includes all datasets generated in this study. In addition, data is available for further exploration at the CKI App, https://schurerlab.shinyapps.io/CKIApp/. Code has been deposited to https://github.com/schurerlab/CKI.

METHOD DETAILS

Inclusion Criteria: TCGA Datasets

The TCGA data portal contains the molecular data of over 20,000 tumor and matched normal samples for 33 cancer types from over 11,000 patients (https://www.cancer.gov/about-nci/organization/ccg/research/structural-genomics/tcga). Our inclusion criteria for kinase analyses were based on the availability of data including normal samples available for differential gene expression analysis, mutational hotspot analysis, clinical data and survival data. First, we only considered solid tumors, thus excluding LAML and DLBC. 9 Cancers (OV, THYM, UVM, SARC, PCPG, UCEC, GBM, UCS and LGG) have been excluded from the complete scoring system due to lack of clinical data (TNM staging). 5 Cancers have only clinical and survival data (ACC, SKCM, PAAD, MESO, and TGCT) but not enough normal samples (< 3 samples) to perform a differential gene expression analysis or mutational analysis. 20 cancers had full gene expression data with normal and tumor samples for DGE analysis. 17 Cancers had the full requirements for scoring for this paper, including clinical and survival data. An overview of the TCGA dataset with number of normal samples, tumor samples and data availability is compiled in the Supplementary material (Table S1). Abbreviations for all the cancer types used in this paper are in accord with the TCGA data portal.

Inclusion Criteria: Kinases

The complete list of kinases used in this analysis was obtained from Pharos (https://pharos.nih.gov/).[12] As of June 2019, 634 kinases were included in our initial analyses; this list contained protein kinases, non-protein kinases and pseudokinases. Several of the kinases annotated in Pharos have different gene names in the TCGA dataset, and needed to be manually curated for our data. Kinase names that could not be mapped to Pharos or whose expression levels were undetectable after expression normalization were excluded from our analyses. Target development levels (TDL) were also obtained from Pharos. Kinases were additionally grouped as "Understudied" or Studied, based off of the IDG designation also available from Pharos. As of June 2019, 151 kinases were annotated as Understudied. However, only 149 of these were included in our analyses (MAP3K21 and STK19 were excluded for above reasons).

Kinases were annotated, per cancer, as MOA targets of approved drugs indicated for the treatment of that specific cancer. For example, while BRAF may have a Tclin annotation, it is not a primary clinical drug target for Breast Cancer (BRCA) treatment.

Data for drug target MOA and drug indication was obtained from DrugCentral (https://drugcentral.org/)[1,3] and “A Comprehensive Map of Molecular Targets”[75] (Table S2). BLCA was modified to include FGFR1/2/3/4 due to the 2019 approval of Erdafitinib.[76] Kinase group and family information were obtained from Kinase Drug Target Ontology (DTO) database.[18]

Tchem and Tclin kinases were further annotated by clinical trial status using the PKIDB[14] in conjunction with Drug Bank[13] and Drug Central.[1,3] For example, if a kinase target was under a clinical trial investigation, the cancer indication was also mapped to the CKI data (Table S2).

Data Extraction and Preprocessing

The TCGA data were downloaded from recount2[77] where TCGA clinical data are also available for all 31 cancers. Differential gene expression analysis was performed for 20 of the 31 cancer types. Datasets with at least three normal (control) samples and three tumor samples were considered, and all other datasets were discarded. For 12 cancers (ACC, LGG, SARC, DLBC, THYM, OV, SKCM, LAML, UVM, PAAD, TGCT, UCS) data were not available for normal samples or were below three normal samples thus preventing the comparison between normal and tumor samples. The data downloaded from recount2 was pre-processed by removing the duplicated TCGA patient IDs or barcodes in each cancer type. Further, the counts were scaled and the lowly expressed genes filtered out using R limma package.[78] The resultant datasets for each cancer were used for differential expression (DE) analysis, survival analysis, identification of mutational hotspots, and analysis of clinical significance (TNM, pathology, histology).

Clinical Data

Patient data for AJCC pathological stage[9] and histological grade for each cancer type was obtained from the standardized clinical dataset, the TCGA Pan-Cancer Clinical Data Resource[79] T,N, and M staging was obtained from the GDC[80] database using the R/Bioconductor[81] package recount2.[77] Unique patient IDs were matched using R for each cancer type to include only primary tumor samples for clinical analyses (Sample code “01”). Secondary or metastatic tumors were not considered in this analysis.

Differential Gene Expression Analysis

For the differential expression analysis, we applied the TCGAbiolinks pipeline on the filtered genes for 20 cancers.[82] Genes with $log_2FC > 1$ and FDR threshold of 0.01 were considered significantly differentially expressed. The significantly differentially expressed genes were mapped to the kinases per cancer. Specifically, only the overexpressed kinases from each cancer type were further considered for the scoring analysis.

Survival Analysis

In addition to gene expression analysis across cancer samples, survival analysis based on gene expression levels was performed. Available TCGA patient data were used to generate Kaplan-Meier (KM) survival plots. For the plots, patient clinical data was obtained with, i) patient vital status (alive or dead), ii) time (if patient is al then, “days_to_last_follow_up,” if patient is dead then, “days_to_death”), and iii) expression level. Patients with no vital status or follow_up data were considered censored.

For each kinase, the Kaplan-Meier (KM) survival plot was generated across each cancer type, applying the survival R package.[83] The patients were categorized into two distinct groups, high expression (upper quartile) and low expression (lower quartile). The resulted KM survival curves were compared by log-rank test obtaining a P value, which indicates statistical significance of survival samples. Kinases with high expression and significant P value were used for score analysis.

Mutational Hotspot Analysis

Mutation somatic variants data was obtained from the GDC data portal in the Mutation Annotation Format (MAF) for each cancer type.[53] The pre-compiled TCGA MAF objects including somatic mutation along with clinical information were downloaded from the TCGAmutation R package.[81] All Kinases were mapped to each cancer type using R. In addition, the function oncodrive was applied to identify cancer genes (kinase drivers) from a given MAF file.[53] Oncodrive is a function based on oncodriveCLUST algorithm, which has been implemented in Python.[84] For scoring analysis, specifically, only those kinases were selected for each cancer that had a minimum of 5 mutations and significant P values. Kaplan-Meier curves for mutated or amplified kinases were generated using cBioPortal.[85]

Clinical Analysis

For clinical analysis, Tumor, Node, Metastasis (TNM) staging system developed by American Joint Committee on Cancer AAJC[9]was used. The TNM system classifies cancer:

1. T: by the size, which is further grouped into t1- (t1a, t1a1, t1b, t1b1), t2- (t2a, t2a1, t2a2, t2b, t2c), t3- (t3a, t3b, t3c) and t4- (t4a, t4b, t4c, t4d, t4e).
2. N: involvement of regional lymph nodes, subtypes n0, n1- (n1a, n1b, n1c, n1mi), n2- (n2a, n2c, n3a) and n3 - (n3a, n3b, n3c).
3. M: presence or absence of distant metastasis, sub divided into m0 and m1.

We used ANOVA to identify significant differences between t1-t4 for each of the four metrics. Subsequently, ANOVA was also performed for N and M to see if there are any significant changes between n0-n3 and m0-m1. In addition, the histological grade was also considered for scoring. The grade is a qualitative assessment for differentiated cells under microscope. The differentiated cells are low grade (g1, g2) and dysmorphic and de-differentiated cells are considered high grade (g3, g4). The statistical significance differences between histological grade g1-g4 was calculated using ANOVA.

Scoring

We have developed the Clinical Kinase Index (CKI) to evaluate the prognostic and clinical value of the mRNA expression levels of each kinase in the human kinome. RNASeq data obtained from normal and solid-tumor samples was used to correlate RPKM with advanced tumor staging in clinical, pathological and histological classifications. Since we are mostly interested in the understudied kinome, we opted to not include RPPA or protein expression data since this was unavailable due to lack of specific and validated antibodies for such kinases. Additionally, we recognize that levels of messenger RNA do not necessarily correlate linearly with kinase activity[10] and a future study of the phospho-kinome would offer more insight into effects these kinases have on cancer progression.

The Clinical Kinase Index (CKI) score was generated for each kinase per cancer using 4 parameters: Differential Gene Overexpression, Overall Survival (OS), Mutational Hotspots and Clinical/Pathological Progression. If the kinase was shown to be significantly overexpressed, correlated with negative survival outcomes or significantly mutated, it received a score of 1 (per parameter). A Clinical score was created using an average per each clinical/pathological parameter which include clinical stage, T, N and M staging, and histological staging/grade. For example, ANOVA analysis was used to determine significant differences in the means of expression for each kinase in each T stage (t1 versus t2, t2 versus t3 etc.). If a kinase expression was significantly increased between two T stages, it received a score of 1. If it was significant between multiple T stages, the scores were averaged to have a maximum score of 1. This was repeated for M and N stages as well as other clinical parameters including histological grade, pathological stage and clinical stage. The total clinical score was a sum of these scores, with a maximum score varying per cancer due the differing availability of clinical and pathological data in the TCGA datasets. Final scores were determined by summing all parameters and dividing by the maximum possible score and multiplying by 100% to normalize across all cancer cohorts. In short, a score is only assigned to a kinase if its increased expression correlates with a progression in staging, a decrease in survival or has significant mutations.

Clinical Kinase Index (CKI) Web Application (https://schurerlab.shinyapps.io/CKIApp/)

The Clinical Kinase Index and application was developed and written in R language R 3.3 or higher using the R packages: shiny, RColorBrewer, gplots, plyr, ggplot2, limma, TCGAbiolinks, tidyverse, recount2, edgeR, SummarizedExperiment, devtools, superheat, xlsx, survival, RTCGA.rnaseq, RTCGA.clinical, survminer, maftools, corrplot, colorRamp, and plotly. The construction of the App utilizes Shiny, a framework to build and deploy interactive Web applications in R. The data have been processed using the R Bioconductor databases and packages included within other requirements. The CKI homepage includes the mRNA expression heatmaps of all kinases (and understudied kinases). The application includes a table which can be filtered based off of user input and contains the kinase gene name, cancer of interest, clinical kinase index score, ranking and other meta-data annotations for the target. Through the gene page users can explore the score of a particular kinases of interest taking advantage of all the information available for specific dataset, which can be filtered: TDL, Kinase type, Kinase Group, MOA targets. Additionally, the CKI disease page allows users to generate 1) a table with specific cancer of interest and filter by TDL and understudied kinases. It also develops a plot of particular cancer including 2) Volcano plots to display differentially expressed kinases per cancer. Next, the clinical Data page produces different plots of particular cancer and gene of interest including 1) Boxplots showing the expression of kinases according to TNM staging, and 2) Kaplan Meier curve representing the overall survival levels of each gene per cancer. The data to reproduce the plots can be downloaded on the Download page for future analysis. This includes some data that has not been visualized in the app, such as mutational and CNA analyses. Code is available on GitHub https://github.com/schurerlab/CKI.

DepMap Analysis

Data was downloaded from DepMap Portal.[37] The datasets used were CRISPR (Avana) Public 19Q2, Sanger CRISPR and Combined RNAi, along with the cell line metadata. Using DepMap Cell line metadata, cell lines were extracted using “disease” and “disease subtype” terms to match the corresponding TCGA cohort. Genes were manually annotated as “dependent” for our analyses if the dependency score was less than -1.

External Tools

Gene amplification data was obtained from cBioPortal using all TCGA Provisional Datasets. Focal amplifications were identified using the GISTIC tool from the BROAD Institute.[59] Gene Set Enrichment Analysis was performed using MSigDB.[35] The top 25% scoring kinases for each cancer were used as input. Positional Gene Sets, chemical and genetic perturbations, canonical pathways, KEGG gene sets, cancer modules and oncogenic signatures were selected for analysis. The FDR q-value threshold was 0.05 and the top 100 enriched gene sets were saved.

QUANTIFICATION AND STATISTICAL ANALYSIS

Statistical analyses used are outlined in the paper. Statistical significance was broadly defined as having a p value < 0.05 unless otherwise stated. All reported statistical analyses are available in the supplemental tables with associated p values and adjusted p values.

ADDITIONAL RESOURCES

For further analysis and graphical display of the data generated in this study, the Clinical Kinase Index (CKI) app is available for use: https://schurerlab.shinyapps.io/CKIApp.

Cell Reports Medicine, Volume *1*

Supplemental Information

The Clinical Kinase Index:

A Method to Prioritize Understudied Kinases

as Drug Targets for the Treatment of Cancer

Derek Essegian, Rimpi Khurana, Vasileios Stathias, and Stephan C. Schürer

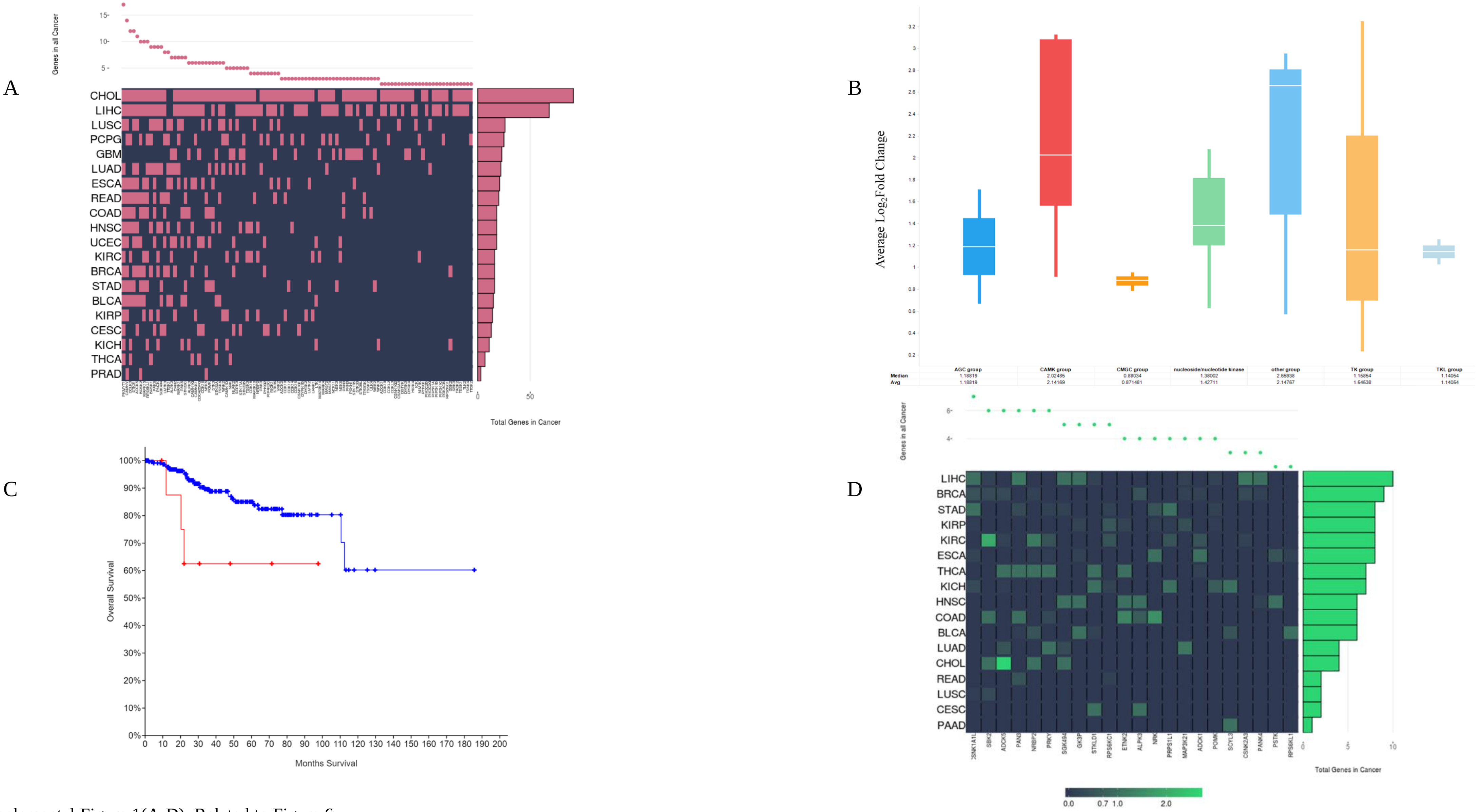

Supplemental Figure 1(A-D). Related to Figure 6.

A: Differential overexpression of Understudied kinases in each cancer cohort. 102 Understudied are significantly overexpressed in at-least two cancers.

B: Several kinase groups appear to be enriched in their overexpression in various cancers. Averaging the log2FC of all kinases across all cancers demonstrates that certain kinase transcripts are consistently very highly upregulated. Kinases from the understudied CAMK group have the highest average log2FC (2.14) and kinases from the Other group have the highest median log2FC (2.66).

C: Dark Kinase DYRK1B was mutated in 4.3% of Endometrial Carcinoma samples (UCEC) with significantly worse overall survival for patients harboring the mutated kinase ($p<.05$).

D: *Tdark* Kinases are potential prognostic biomarkers for several cancers. TNM staging scores (0-3) show that dark kinases contribute to tumor progression.

Supplemental Data 1 (A-P) Analysis of Clinically Relevant Understudied Kinases. Related to Figure 5: CKI scores of targets who have been under or are currently under clinical trial investigation versus those that have no been clinically evaluated in humans. Significant differences in distributions are signified by p-values <.05.

1A.

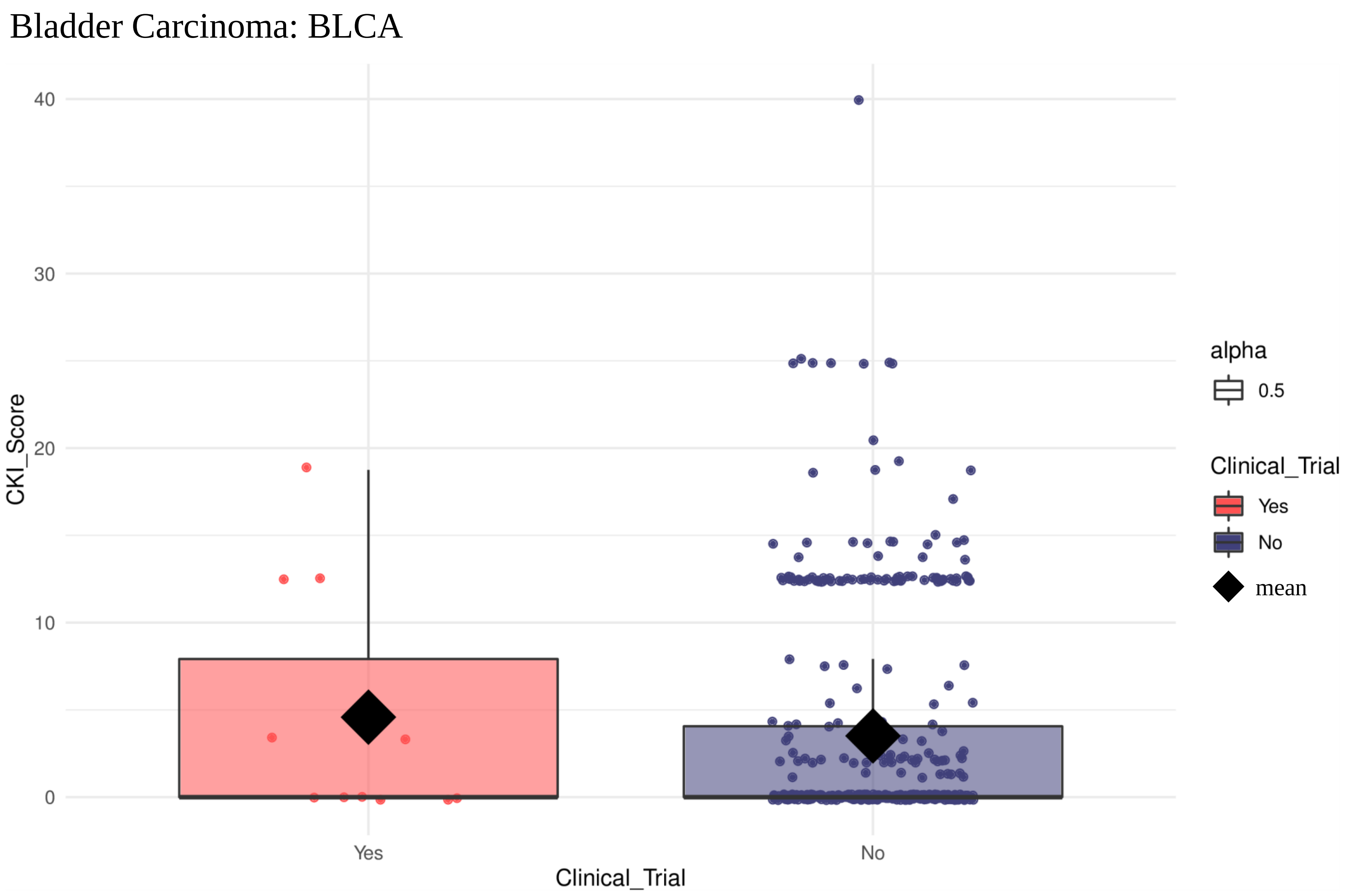
Bladder Carcinoma: BLCA
40
30
20
10
0
CKI_Score
Yes
No
Clinical_Trial
alpha
0.5
Clinical_Trial
Yes
No
mean

1B.

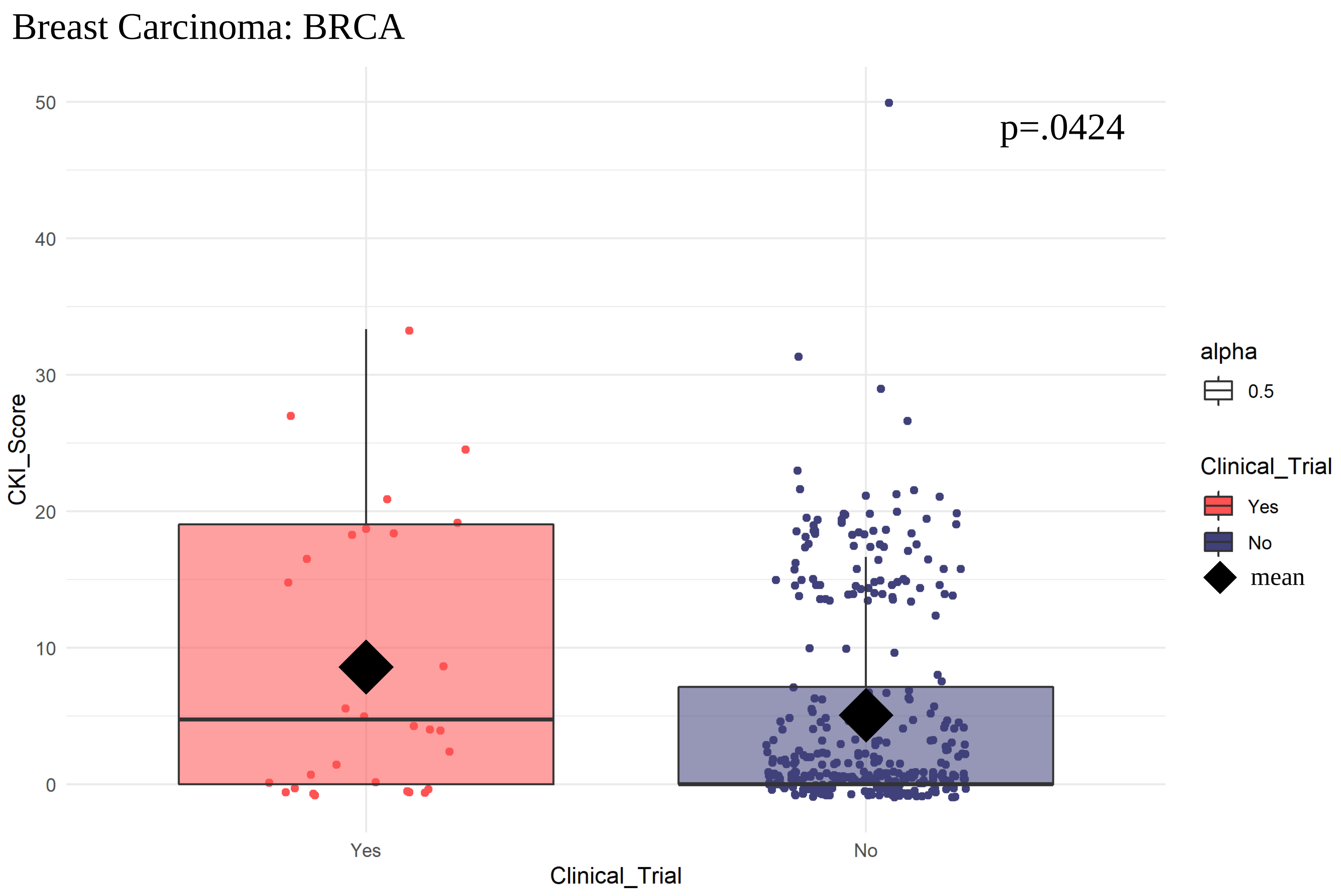

Breast Carcinoma: BRCA
p=.0424
CKI_Score
50
40
30
20
10
0
Yes
No
Clinical_Trial
alpha
0.5
Clinical_Trial
Yes
No
mean

1C.

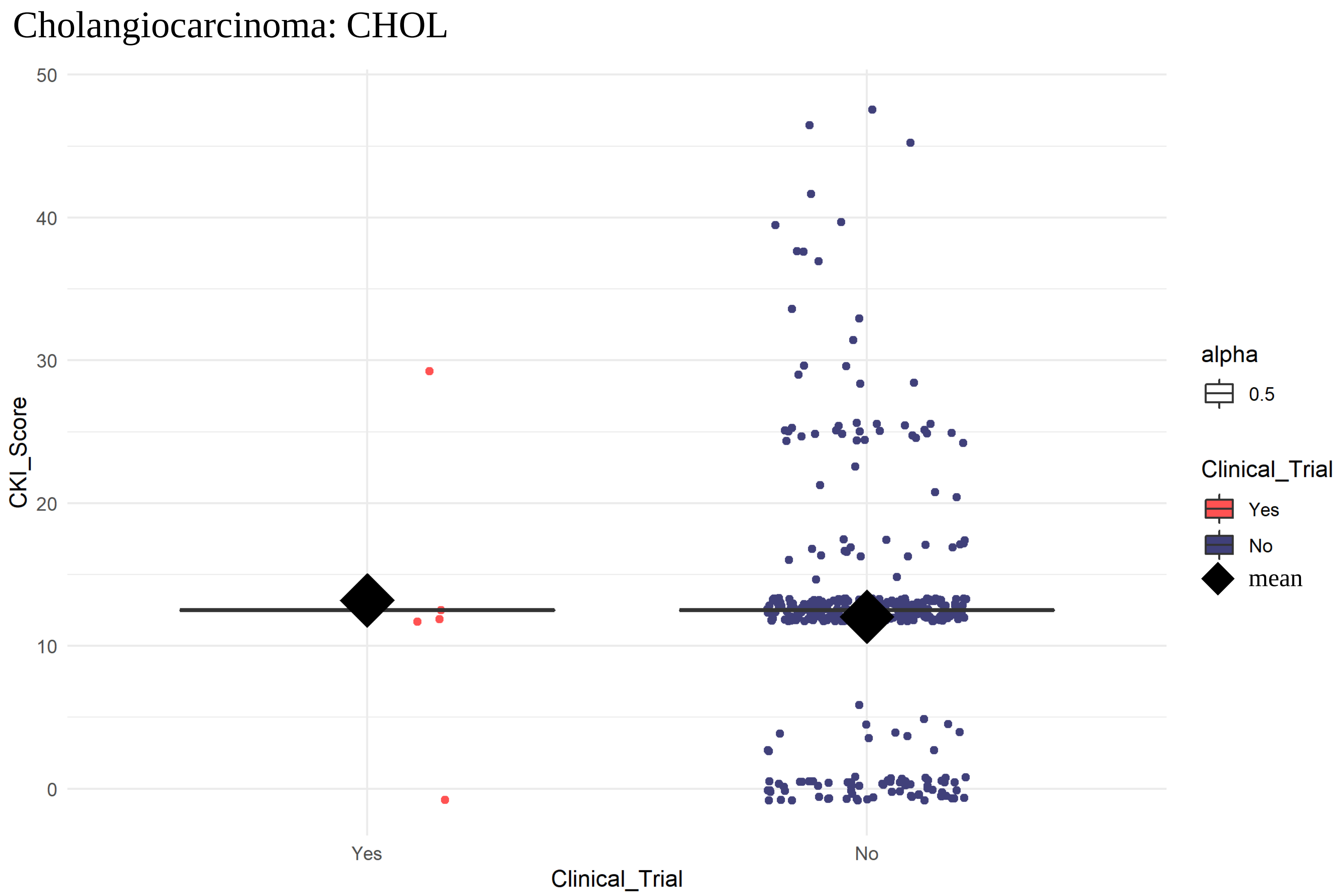

Cholangiocarcinoma: CHOL
50
40
30
20
10
0
CKI_Score
Yes
No
Clinical_Trial
alpha
0.5
Clinical_Trial
Yes
No
mean

1D.

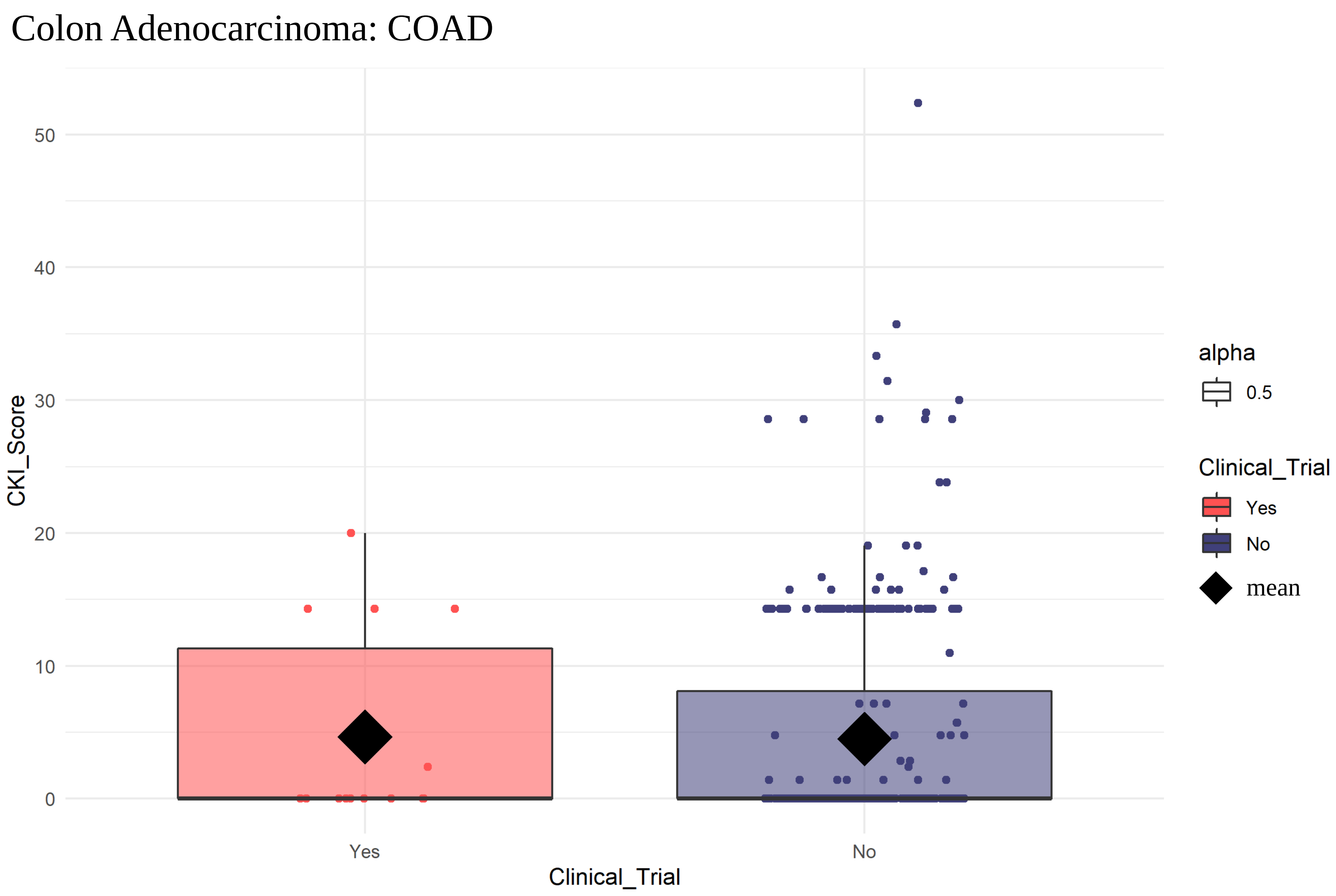
Colon Adenocarcinoma: COAD
50
40
30
20
10
0
CKI_Score
Yes
No
Clinical_Trial
alpha
0.5
Clinical_Trial
Yes
No
mean

1E.

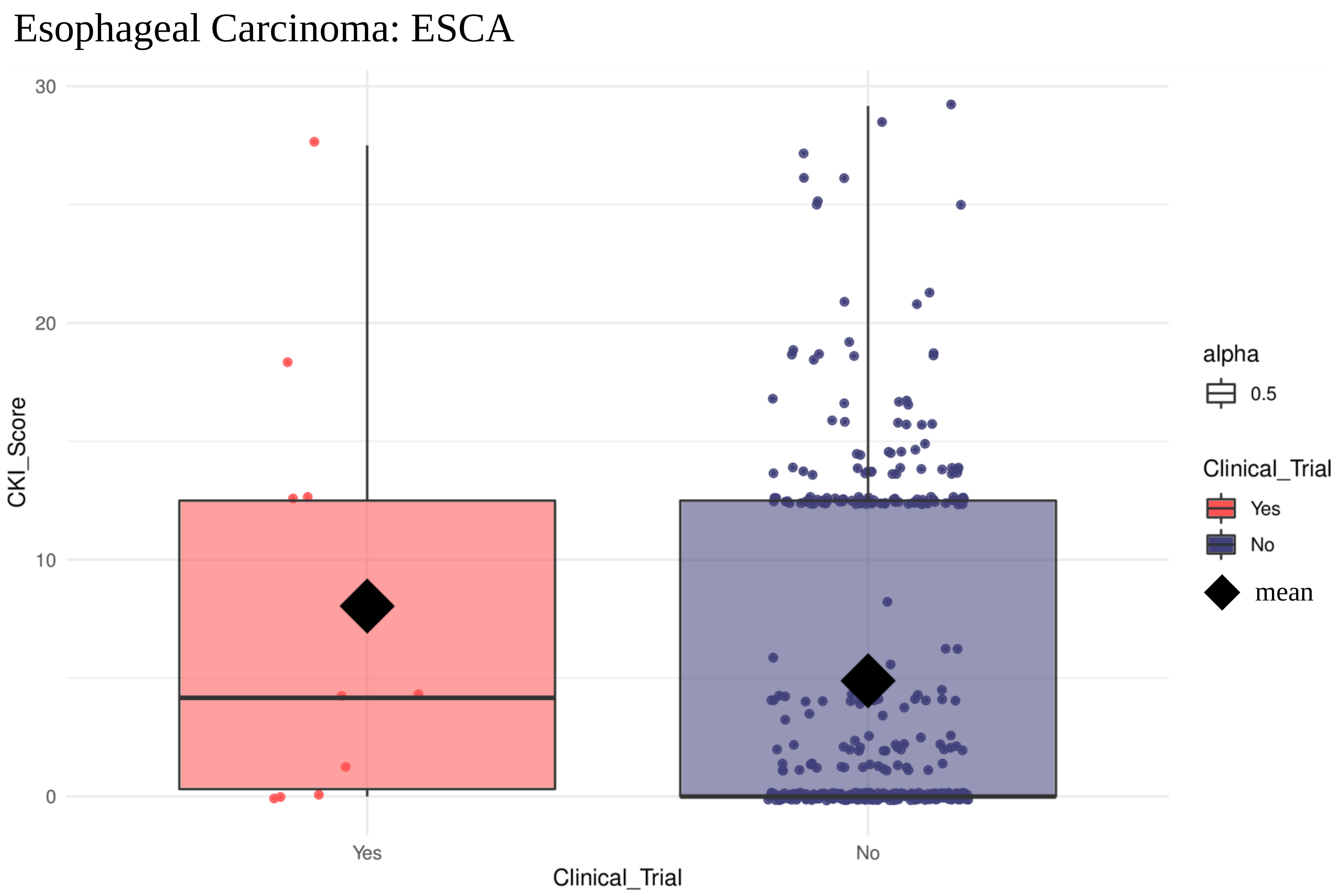

Esophageal Carcinoma: ESCA
30
20
10
0
CKI_Score
Yes
No
Clinical_Trial
alpha
0.5
Clinical_Trial
Yes
No
mean

1F.

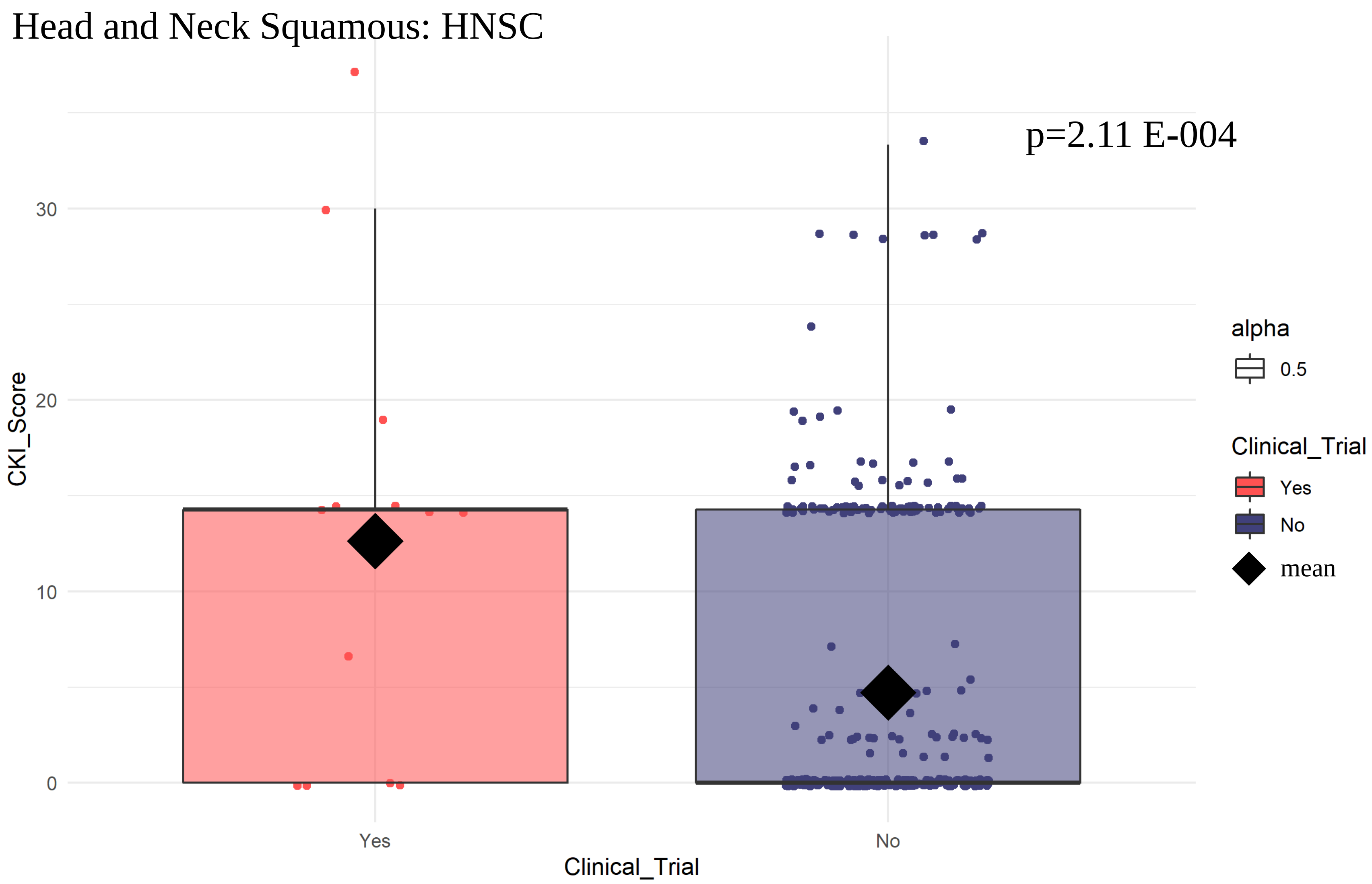
Head and Neck Squamous: HNSC
p=2.11 E-004
30
20
10
0
CKI_Score
Yes
No
Clinical_Trial
alpha
0.5
Clinical_Trial
Yes
No
mean

1G.

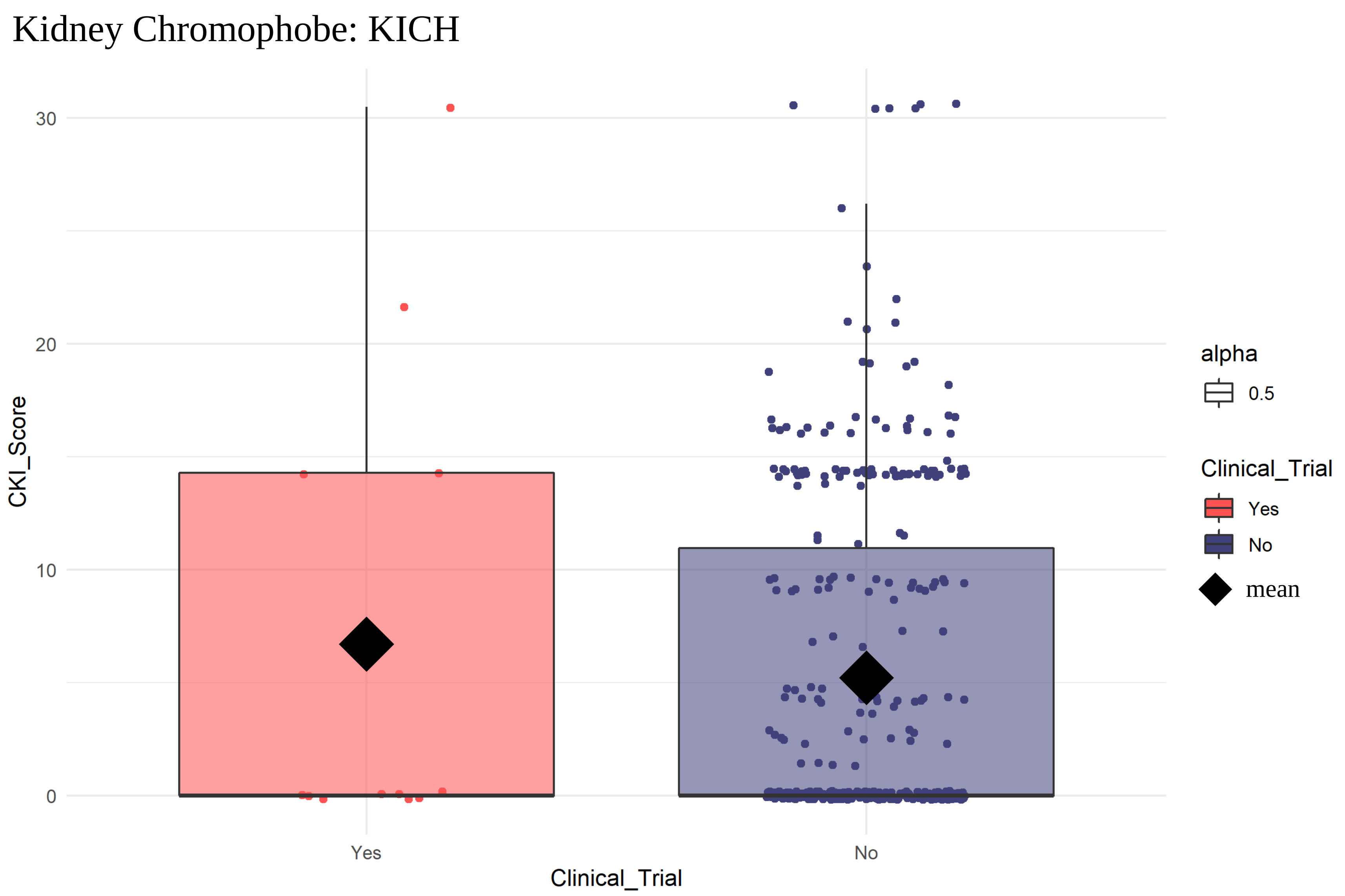
Kidney Chromophobe: KICH
30
20
10
0
CKI_Score
Yes
No
Clinical_Trial
alpha
0.5
Clinical_Trial
Yes
No
mean

1H.

Kidney Renal Clear Cell: KIRC

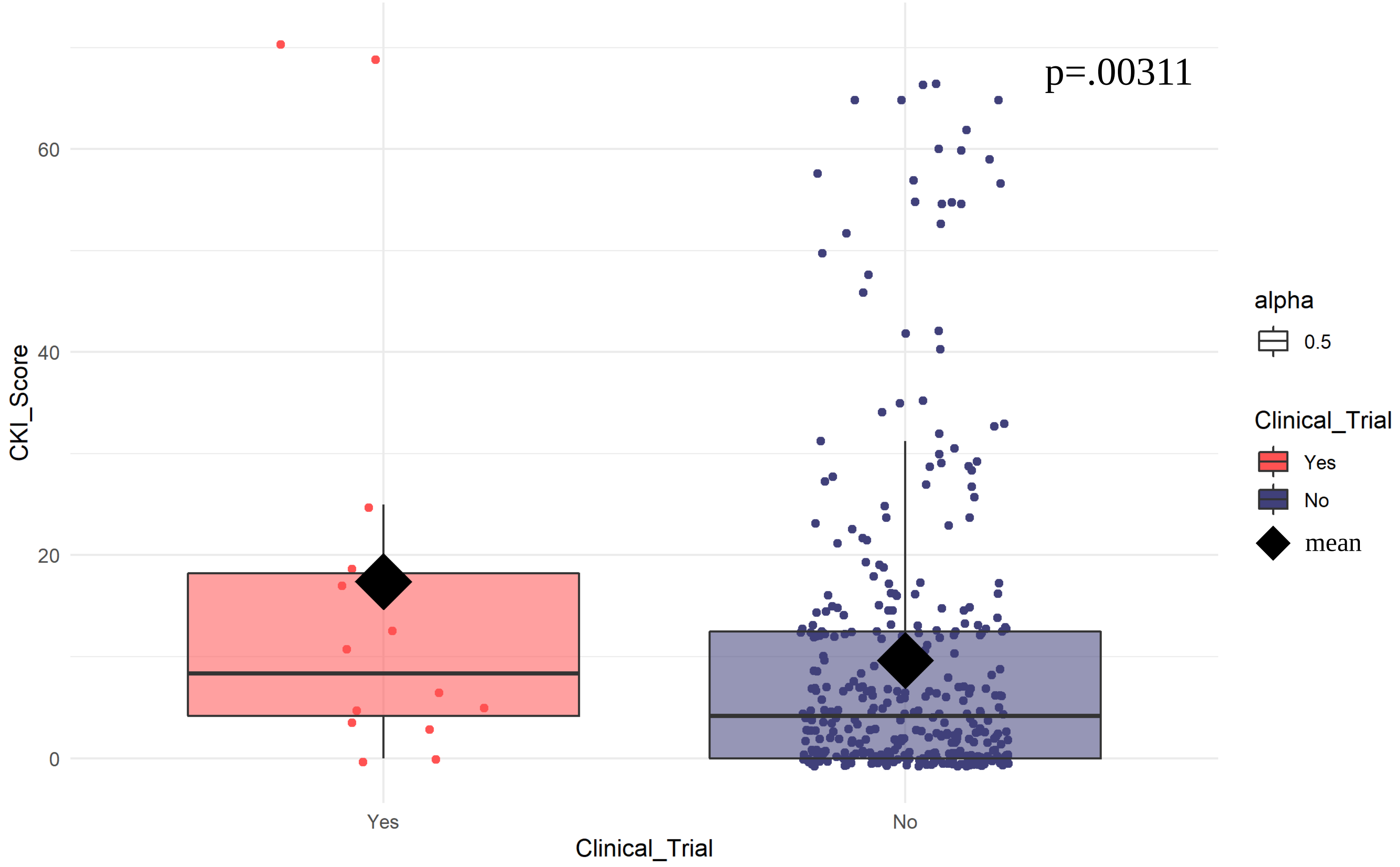

1I.

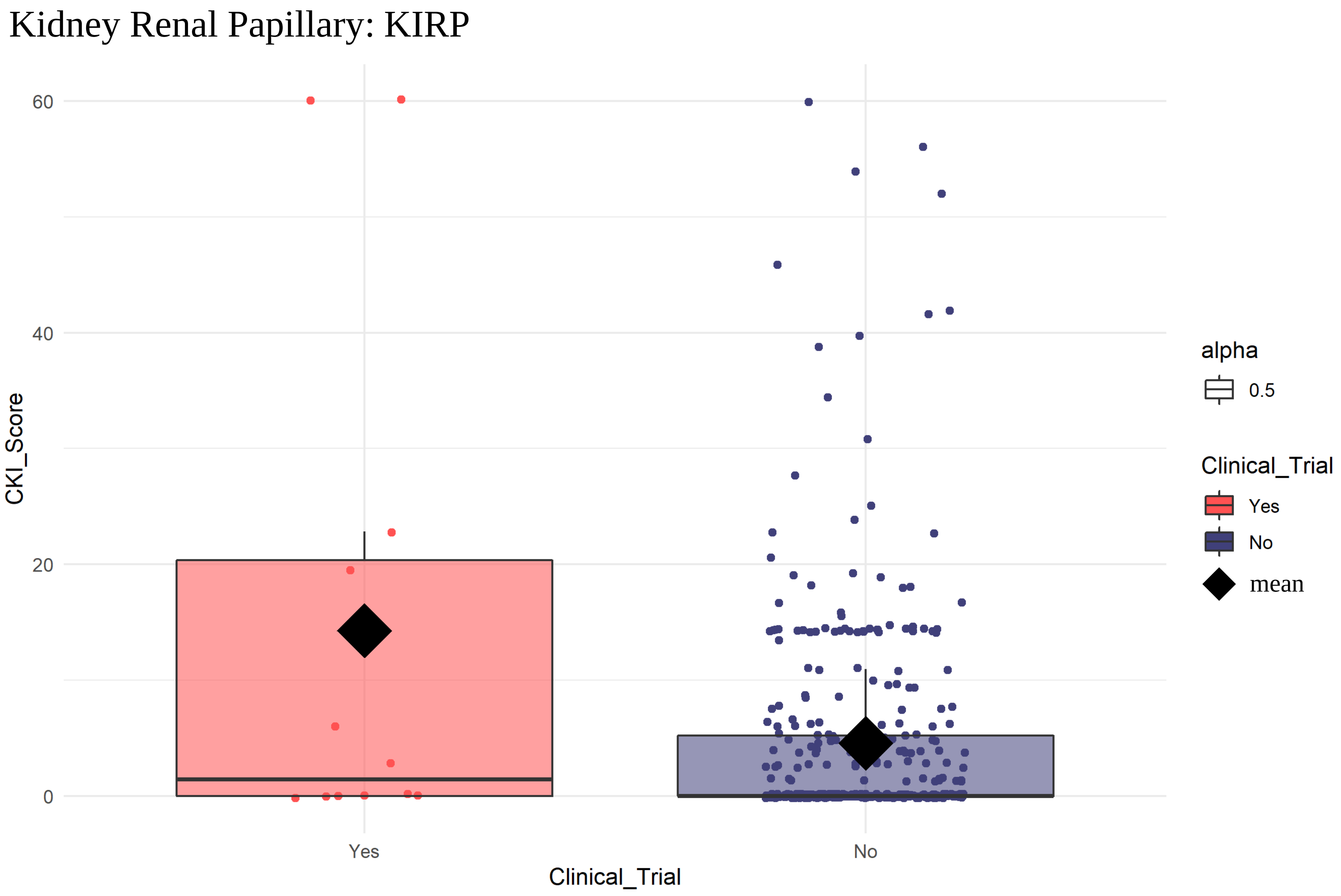

Kidney Renal Papillary: KIRP
60
40
20
0
CKI_Score
Yes
No
Clinical_Trial
alpha
0.5
Clinical_Trial
Yes
No
mean

1J.

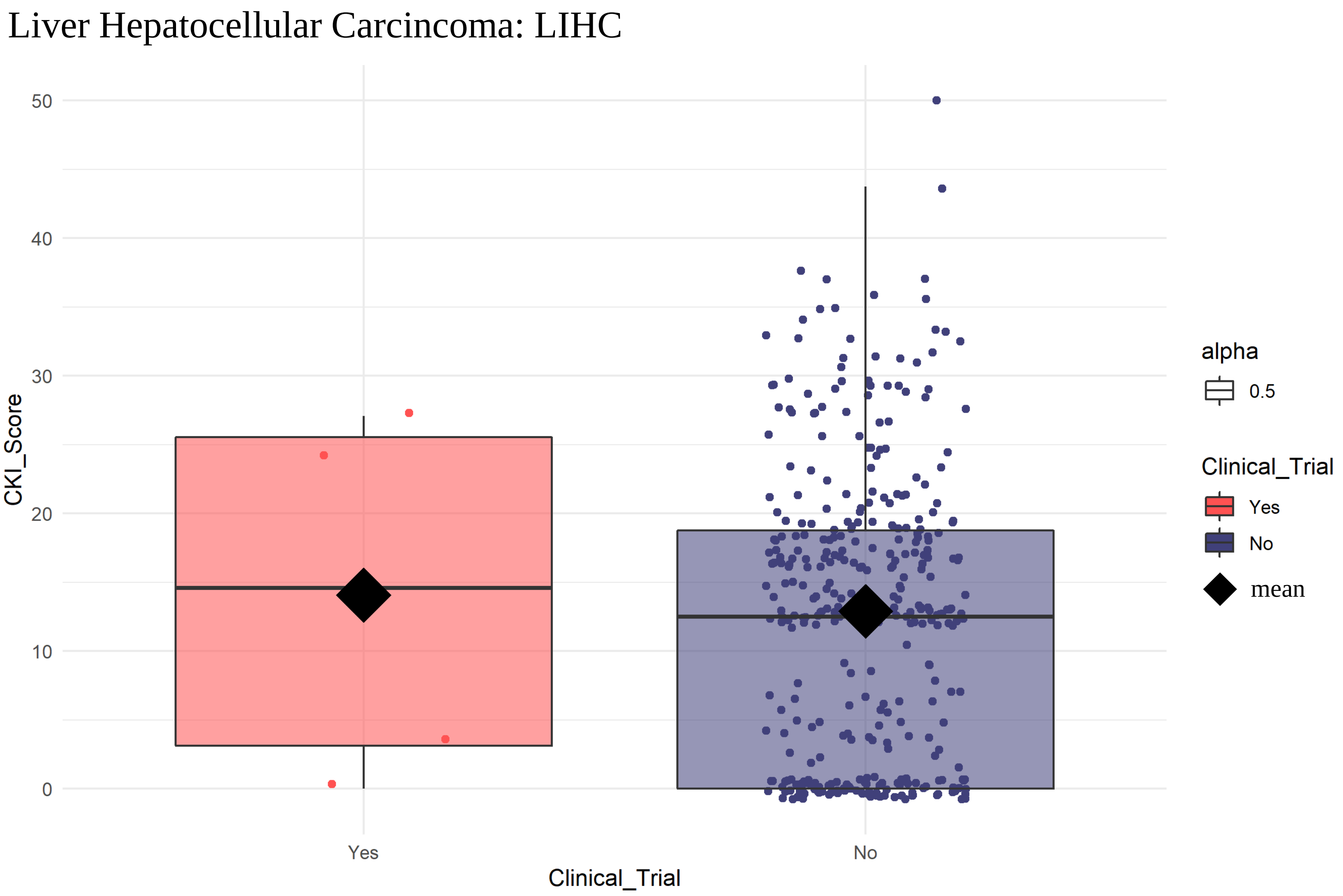

Liver Hepatocellular Carcincoma: LIHC
CKI_Score
50
40
30
20
10
0
Yes
No
Clinical_Trial
alpha
0.5
Clinical_Trial
Yes
No
mean

1K.

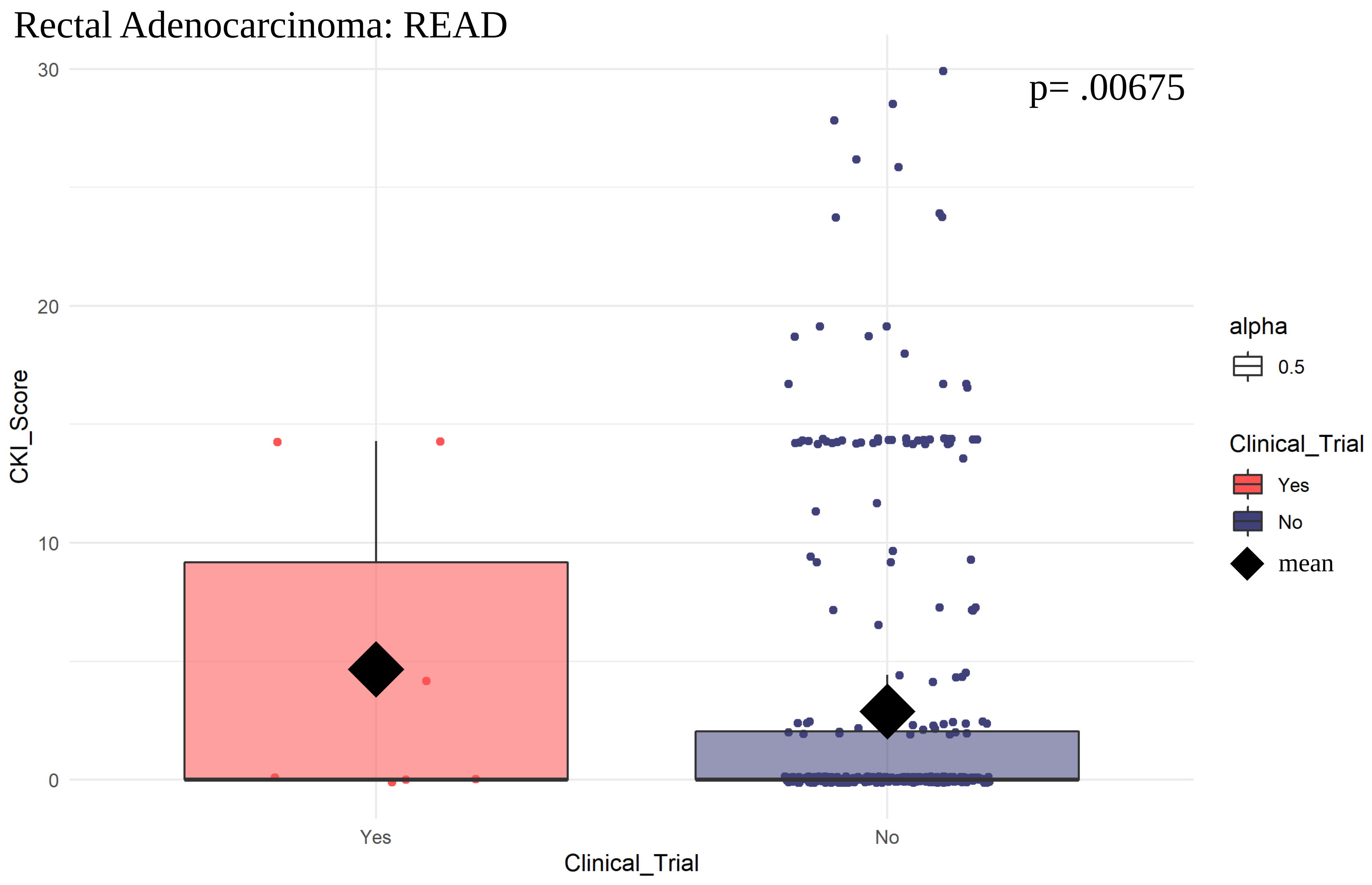

Rectal Adenocarcinoma: READ
p= .00675
30
20
10
0
CKI_Score
Yes
No
Clinical_Trial
alpha
0.5
Clinical_Trial
Yes
No
mean

1L.

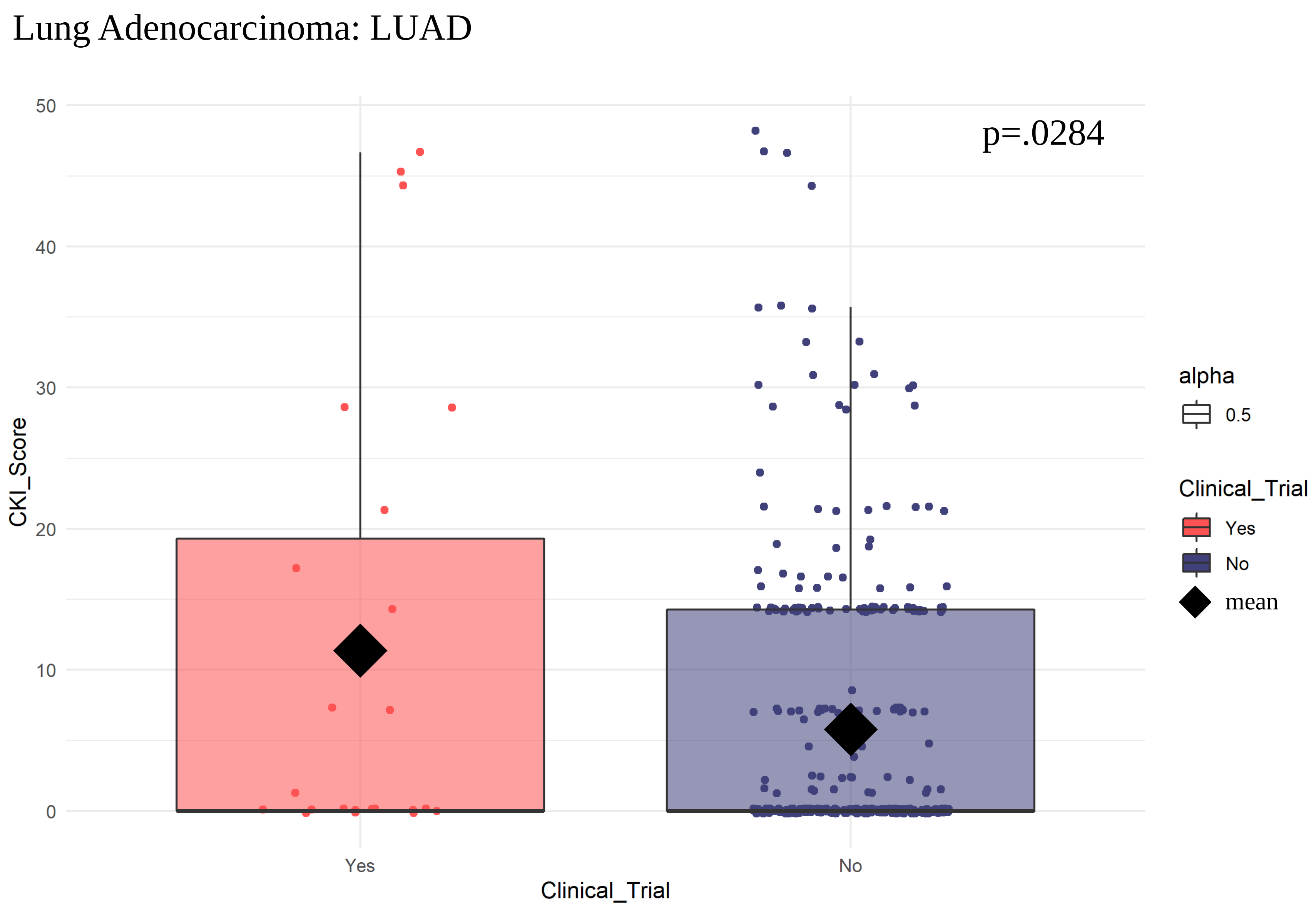

Lung Adenocarcinoma: LUAD
p=.0284
CKI_Score
0
10
20
30
40
50
Yes
No
Clinical_Trial
alpha
0.5
Clinical_Trial
Yes
No
mean

1M.

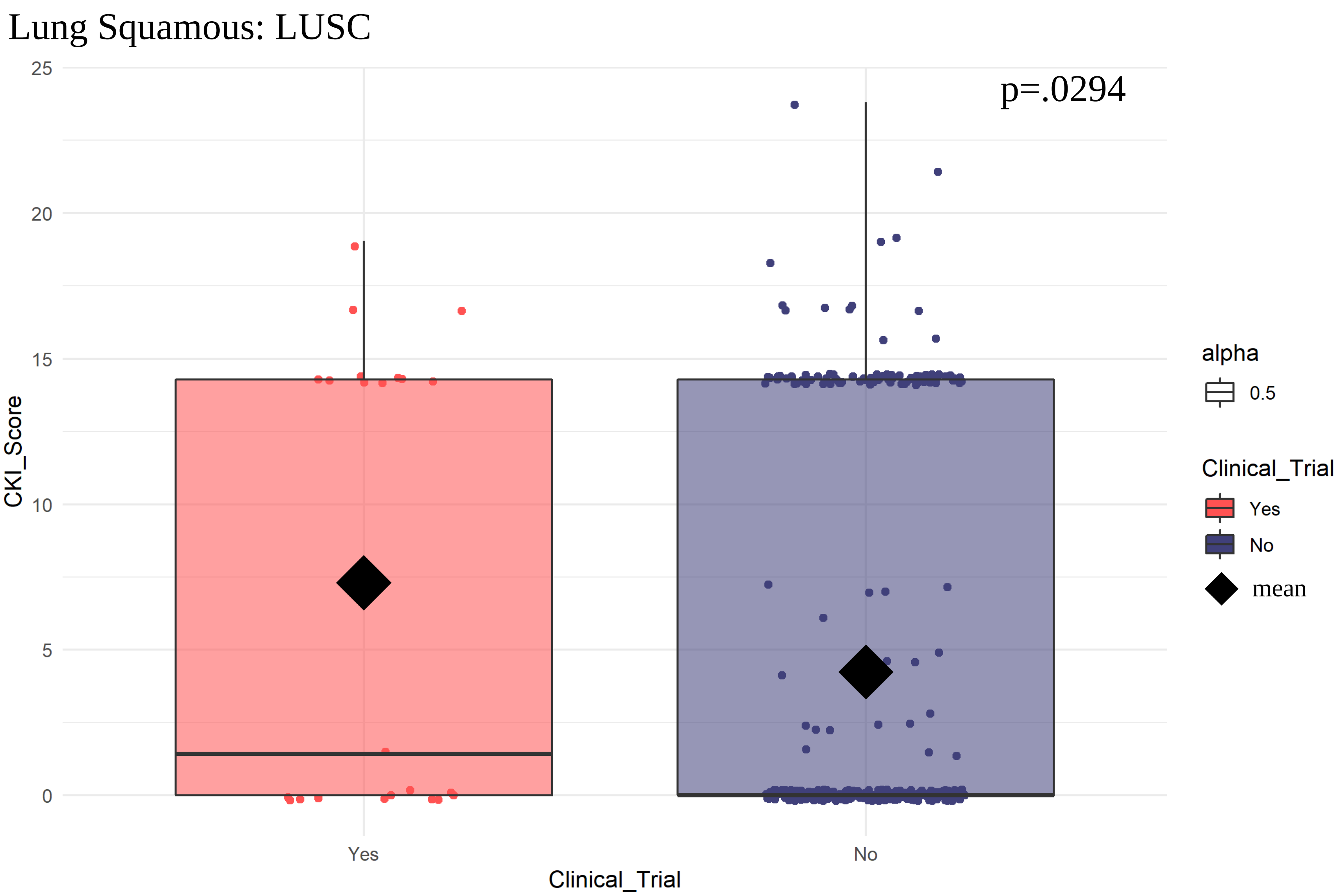

Lung Squamous: LUSC
p=.0294
25
20
15
10
5
0
CKI_Score
Yes
No
Clinical_Trial
alpha
0.5
Clinical_Trial
Yes
No
mean

1N.

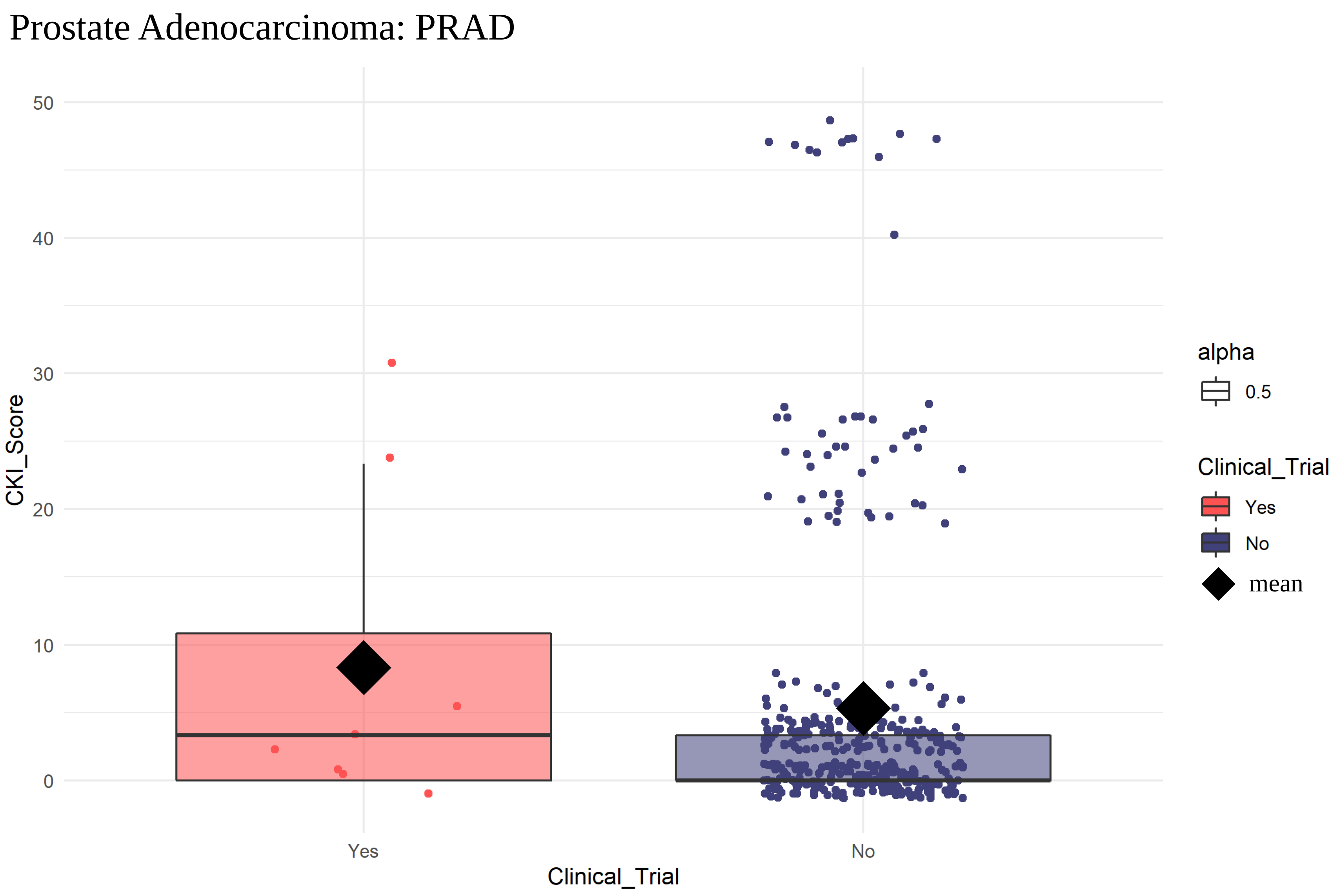

Prostate Adenocarcinoma: PRAD
CKI_Score
50
40
30
20
10
0
Yes
No
Clinical_Trial
alpha
0.5
Clinical_Trial
Yes
No
mean

1O.

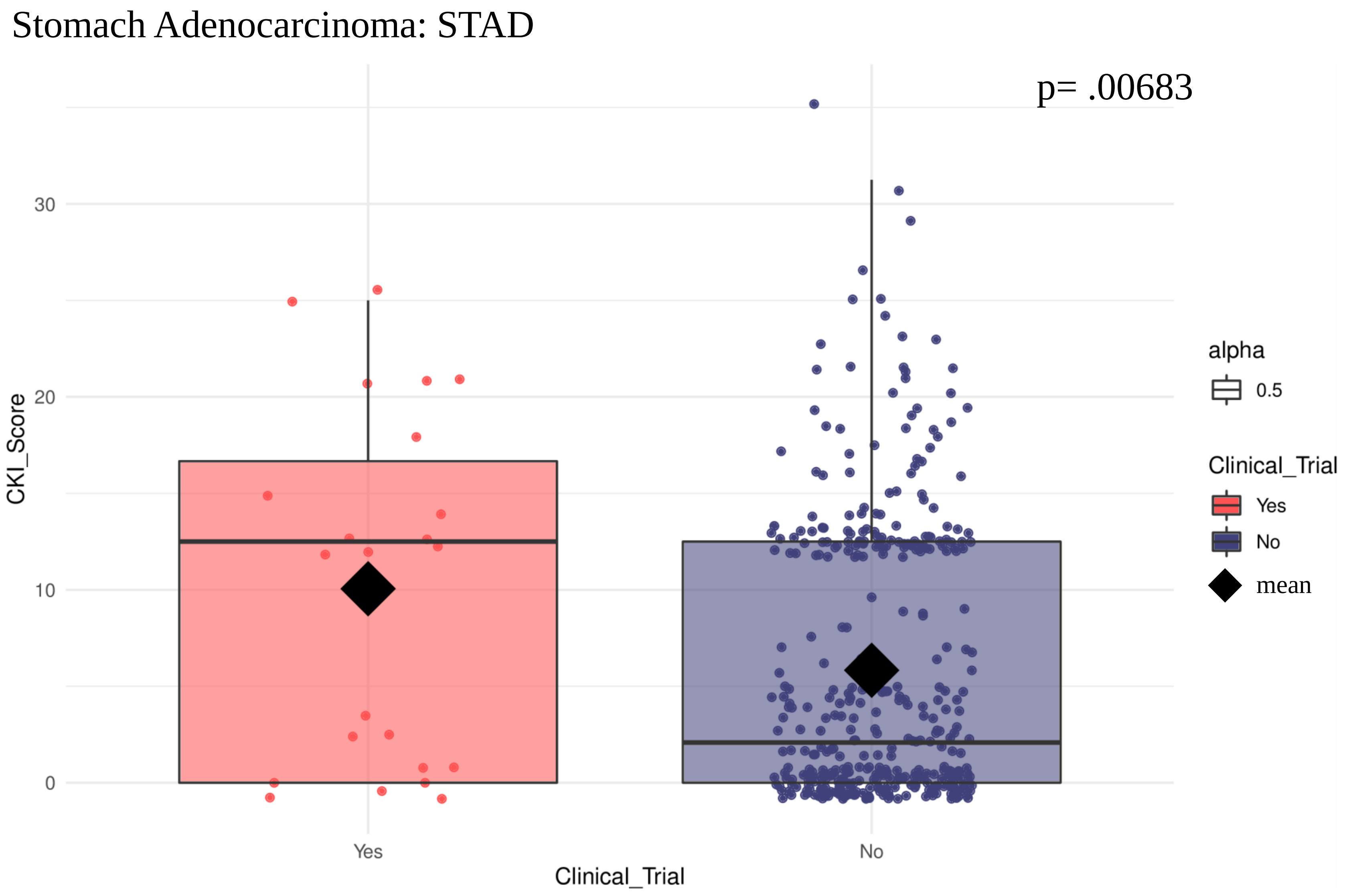

Stomach Adenocarcinoma: STAD
p= .00683
CKI_Score
30
20
10
0
Yes
No
Clinical_Trial
alpha
0.5
Clinical_Trial
Yes
No
mean

1P.

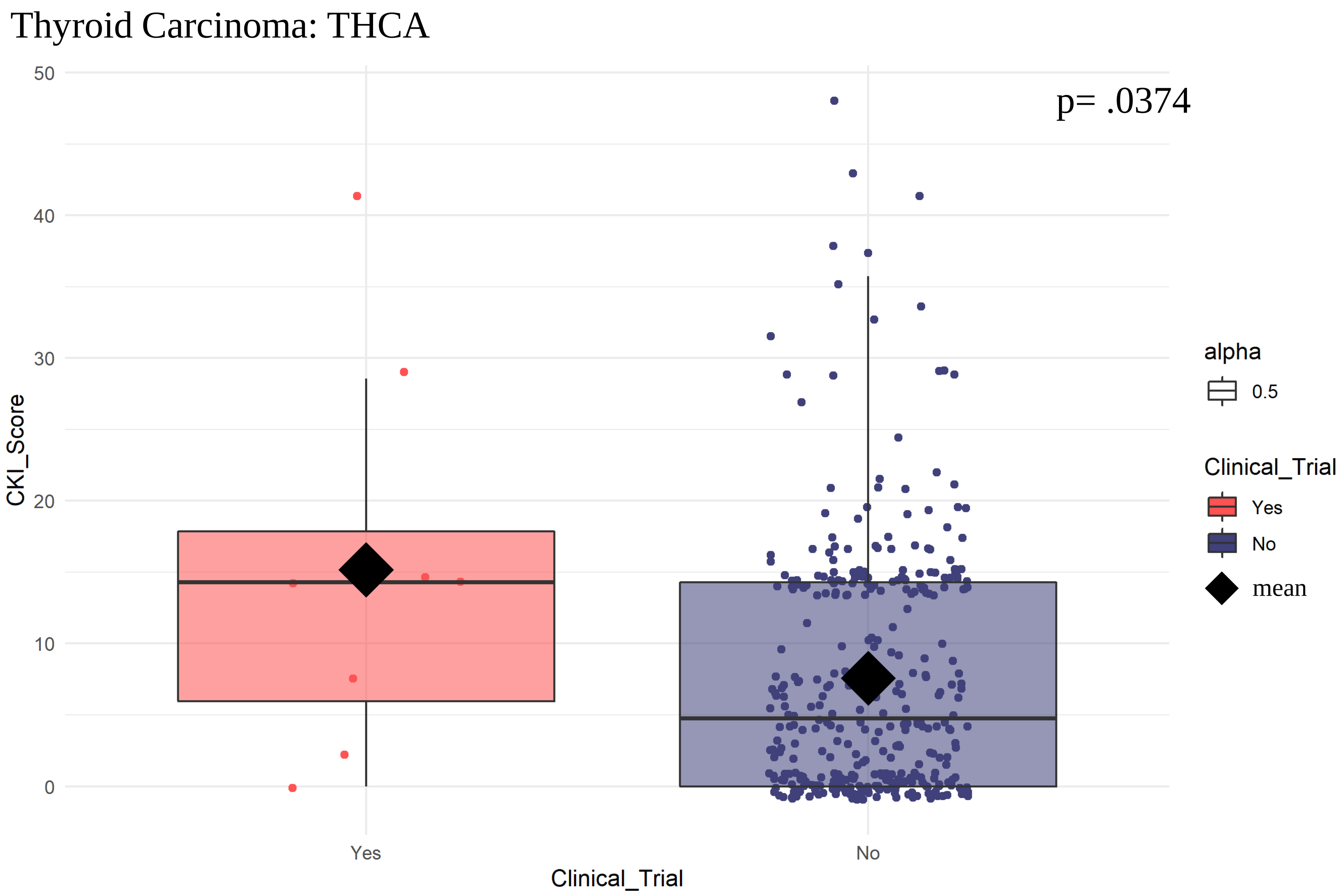
Thyroid Carcinoma: THCA
p= .0374
CKI_Score
50
40
30
20
10
0
Yes
No
Clinical_Trial
alpha
0.5
Clinical_Trial
Yes
No
mean

Supplemental Data 2 (A-N) CKI Score for MOA Targets. Related to Figure 5.

2A.

Average CKI Score per Cancer, TDL

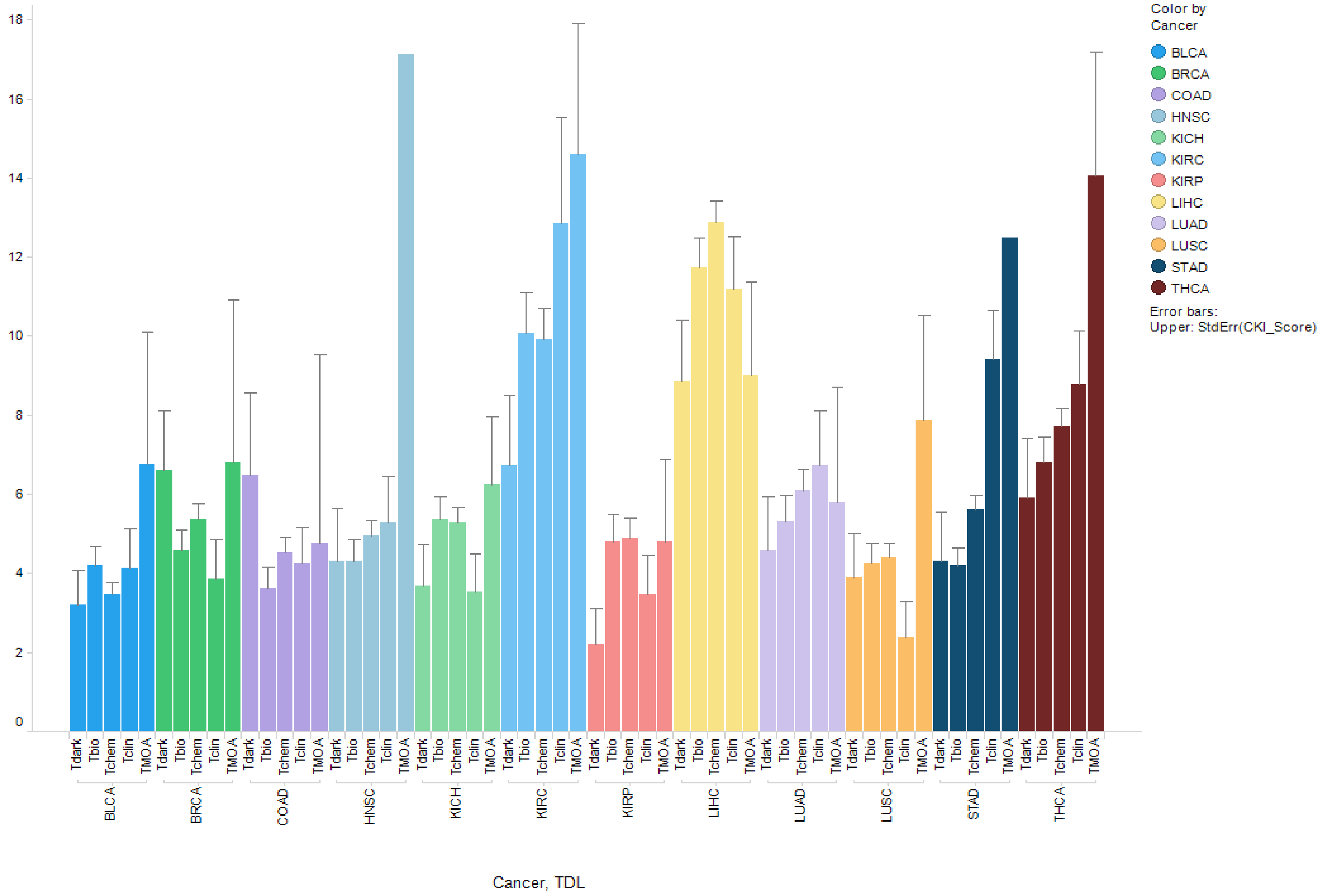

2B.

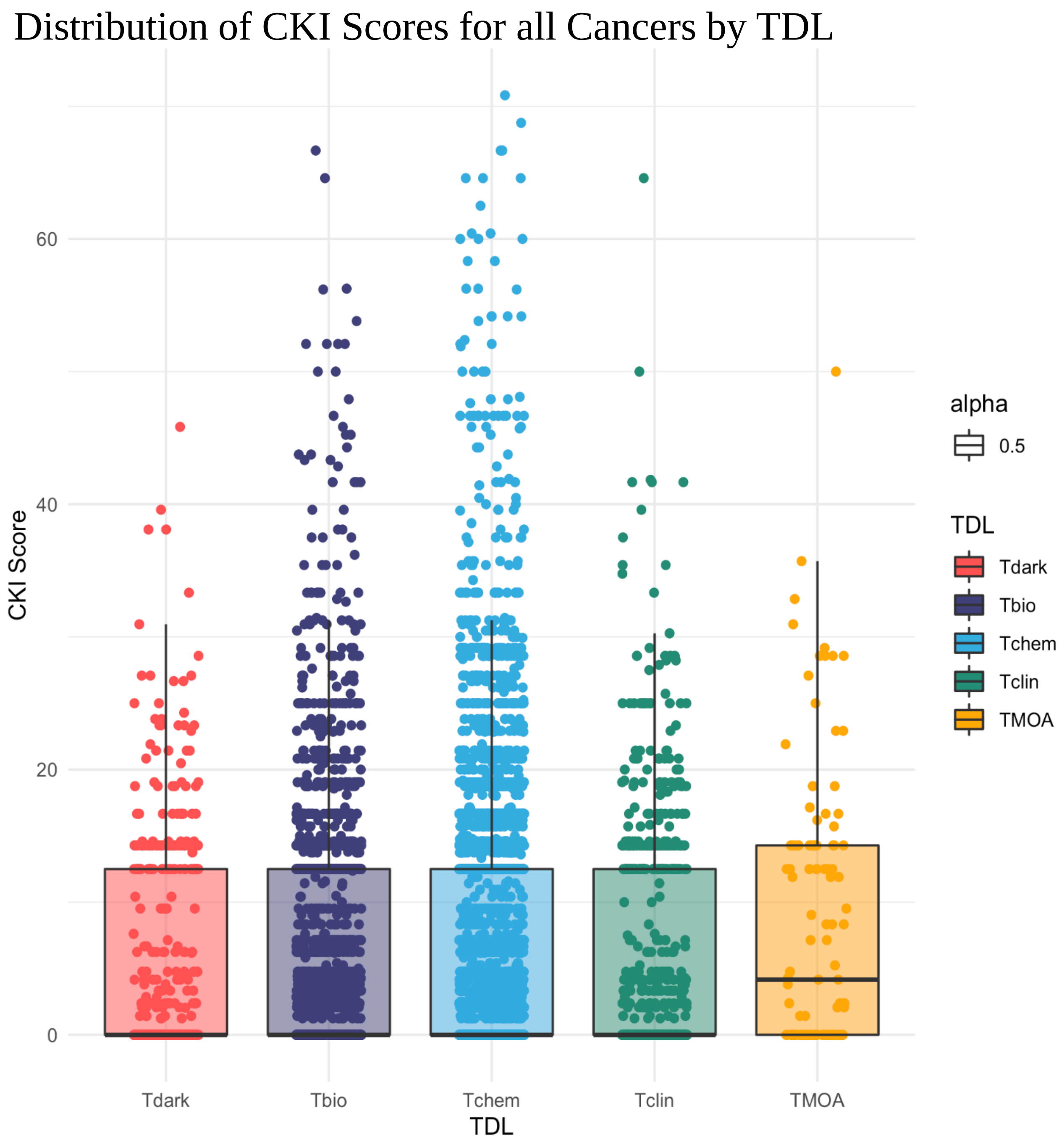
Distribution of CKI Scores for all Cancers by TDL
CKI Score
0
20
40
60
Tdark
Tbio
Tchem
Tclin
TMOA
TDL
alpha
0.5
TDL
Tdark
Tbio
Tchem
Tclin
TMOA

2C.

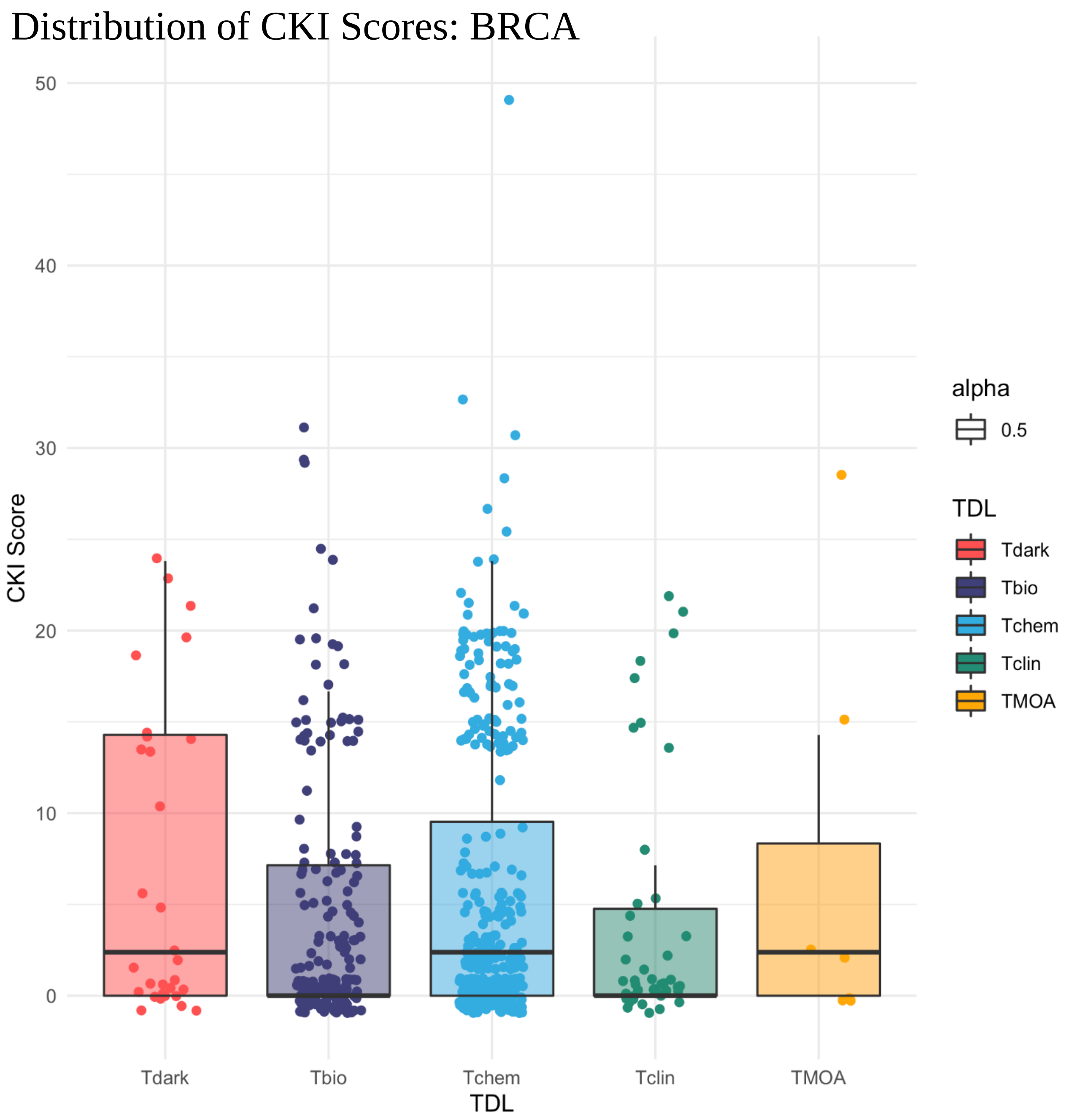
Distribution of CKI Scores: BRCA
CKI Score
50
40
30
20
10
0
Tdark
Tbio
Tchem
Tclin
TMOA
TDL
alpha
0.5
TDL
Tdark
Tbio
Tchem
Tclin
TMOA

2D.

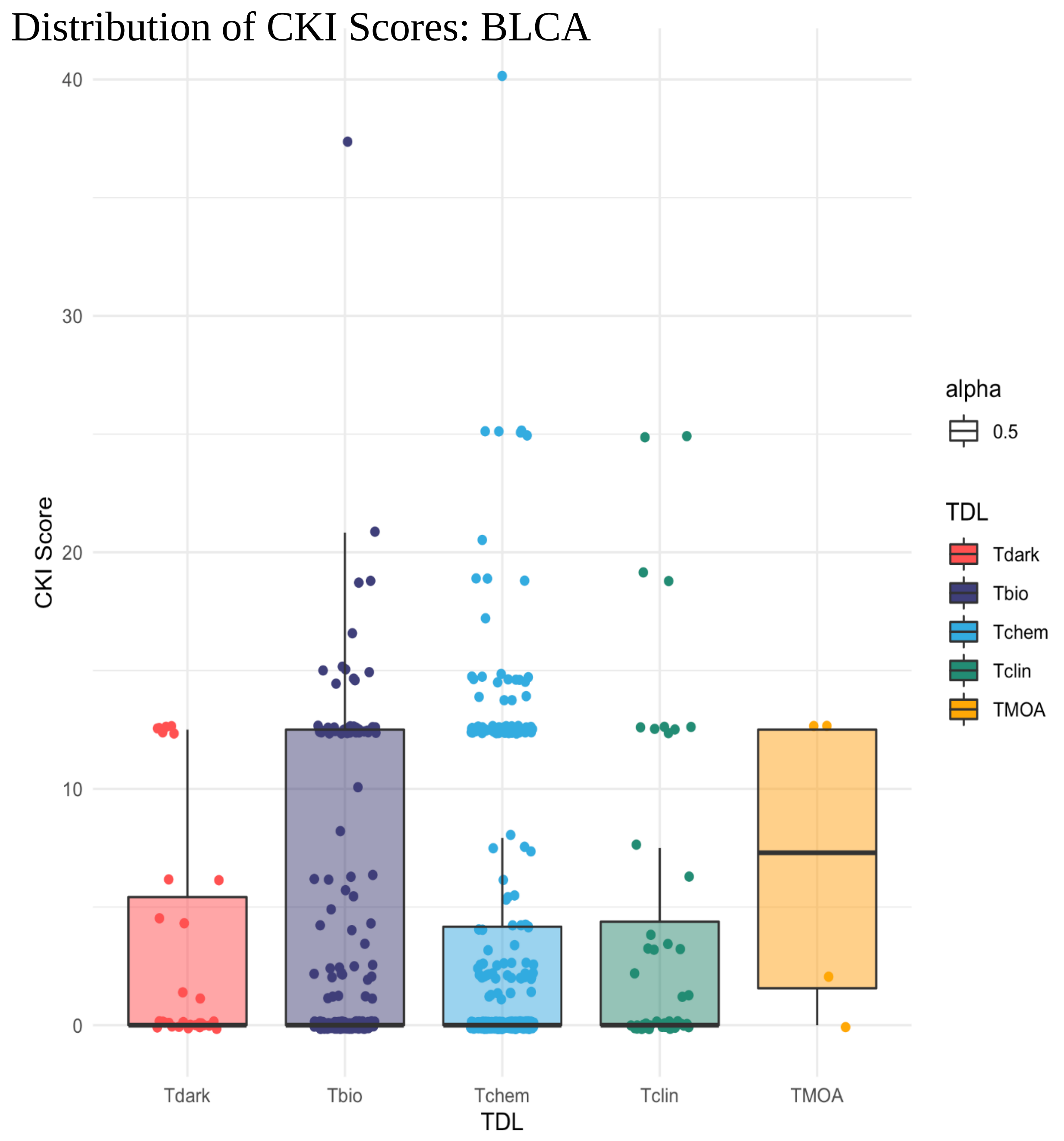
Distribution of CKI Scores: BLCA
CKI Score
40
30
20
10
0
Tdark
Tbio
Tchem
Tclin
TMOA
TDL
alpha
0.5
TDL
Tdark
Tbio
Tchem
Tclin
TMOA

2E.

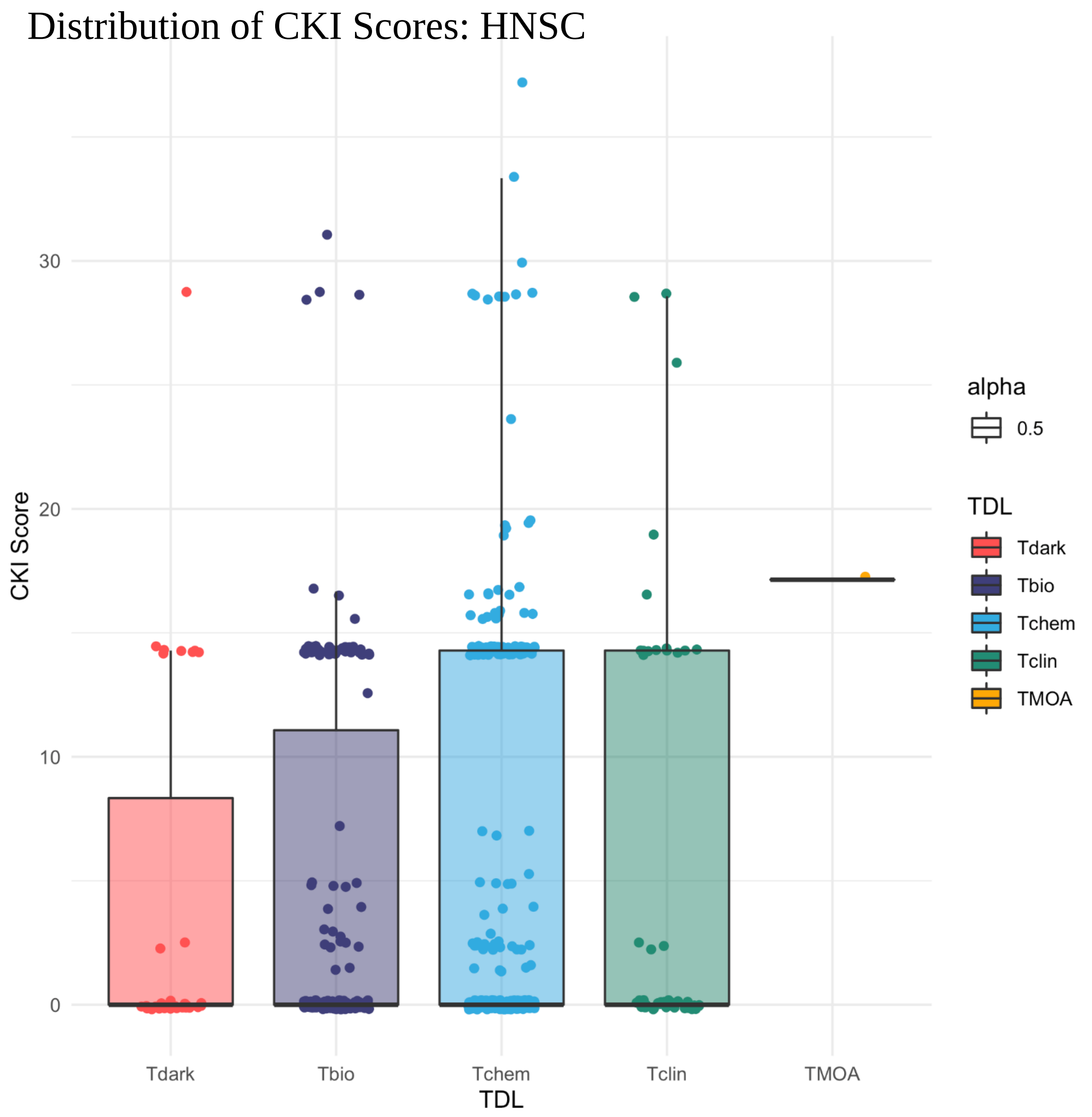

Distribution of CKI Scores: HNSC
CKI Score
0
10
20
30
Tdark
Tbio
Tchem
Tclin
TMOA
TDL
alpha
0.5
TDL
Tdark
Tbio
Tchem
Tclin
TMOA

2F.

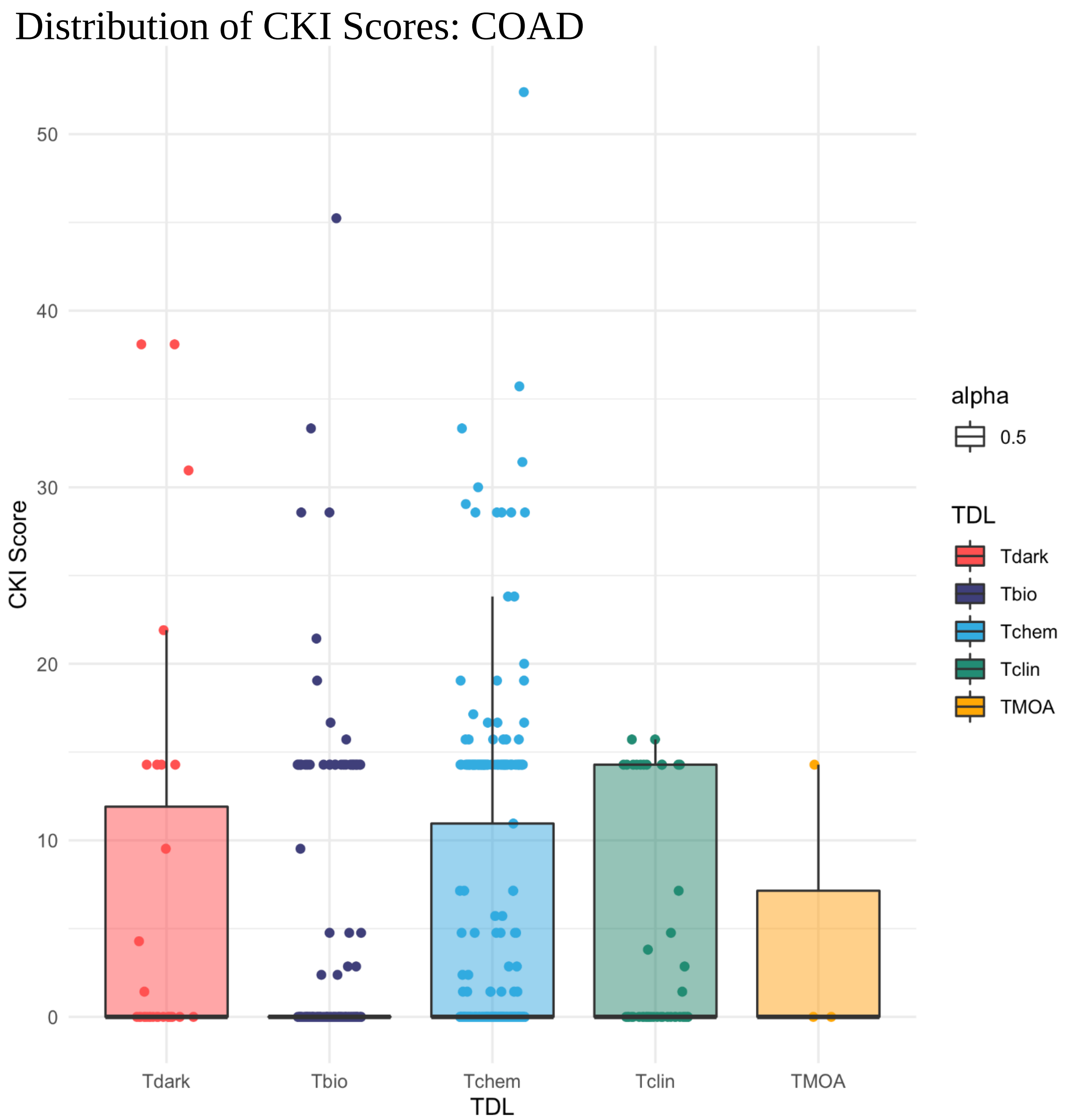
Distribution of CKI Scores: COAD
CKI Score
50
40
30
20
10
0
Tdark
Tbio
Tchem
Tclin
TMOA
TDL
alpha
0.5
TDL
Tdark
Tbio
Tchem
Tclin
TMOA

2G.

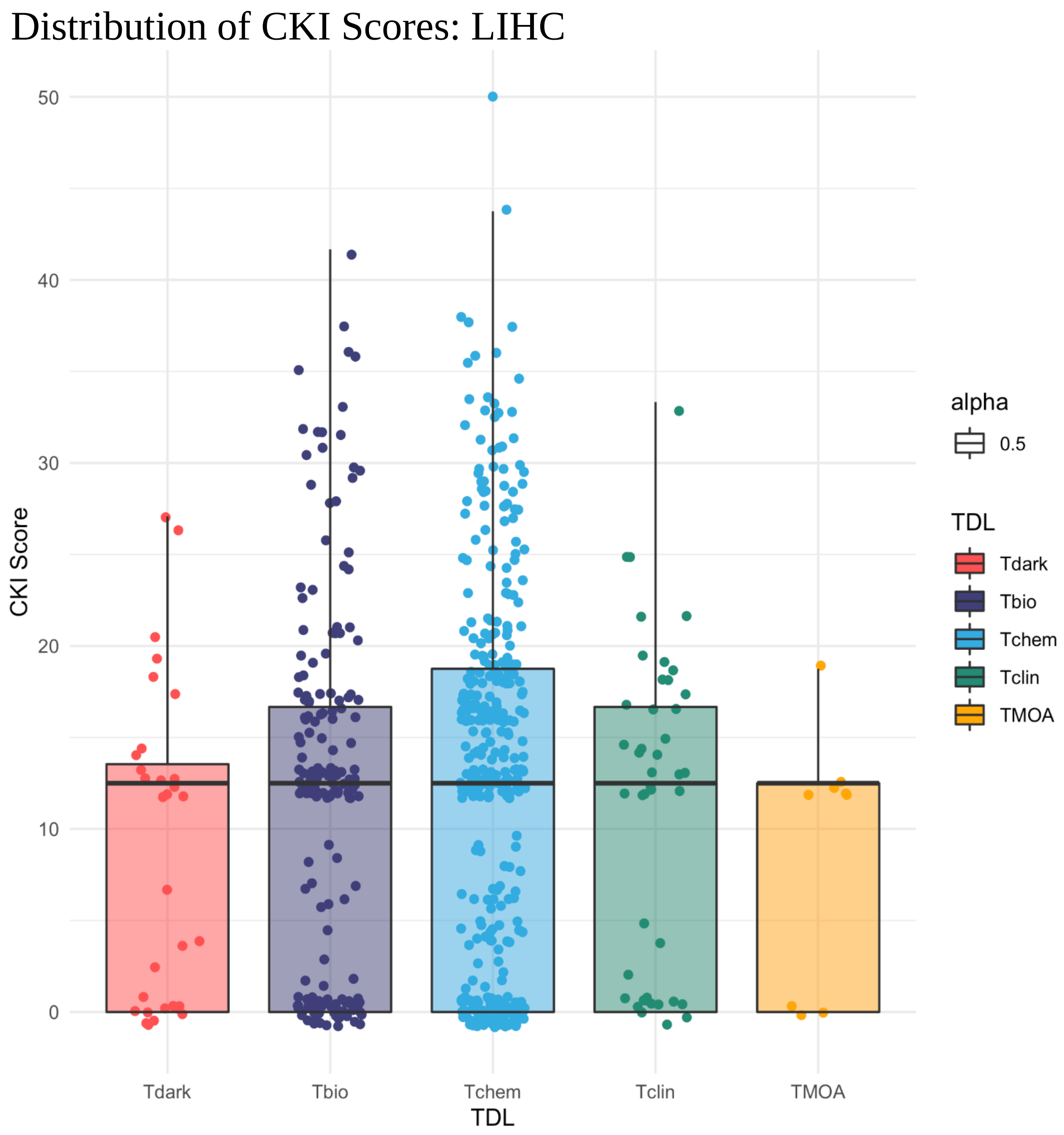

Distribution of CKI Scores: LIHC
CKI Score
50
40
30
20
10
0
Tdark
Tbio
Tchem
Tclin
TMOA
TDL
alpha
0.5
TDL
Tdark
Tbio
Tchem
Tclin
TMOA

2H.

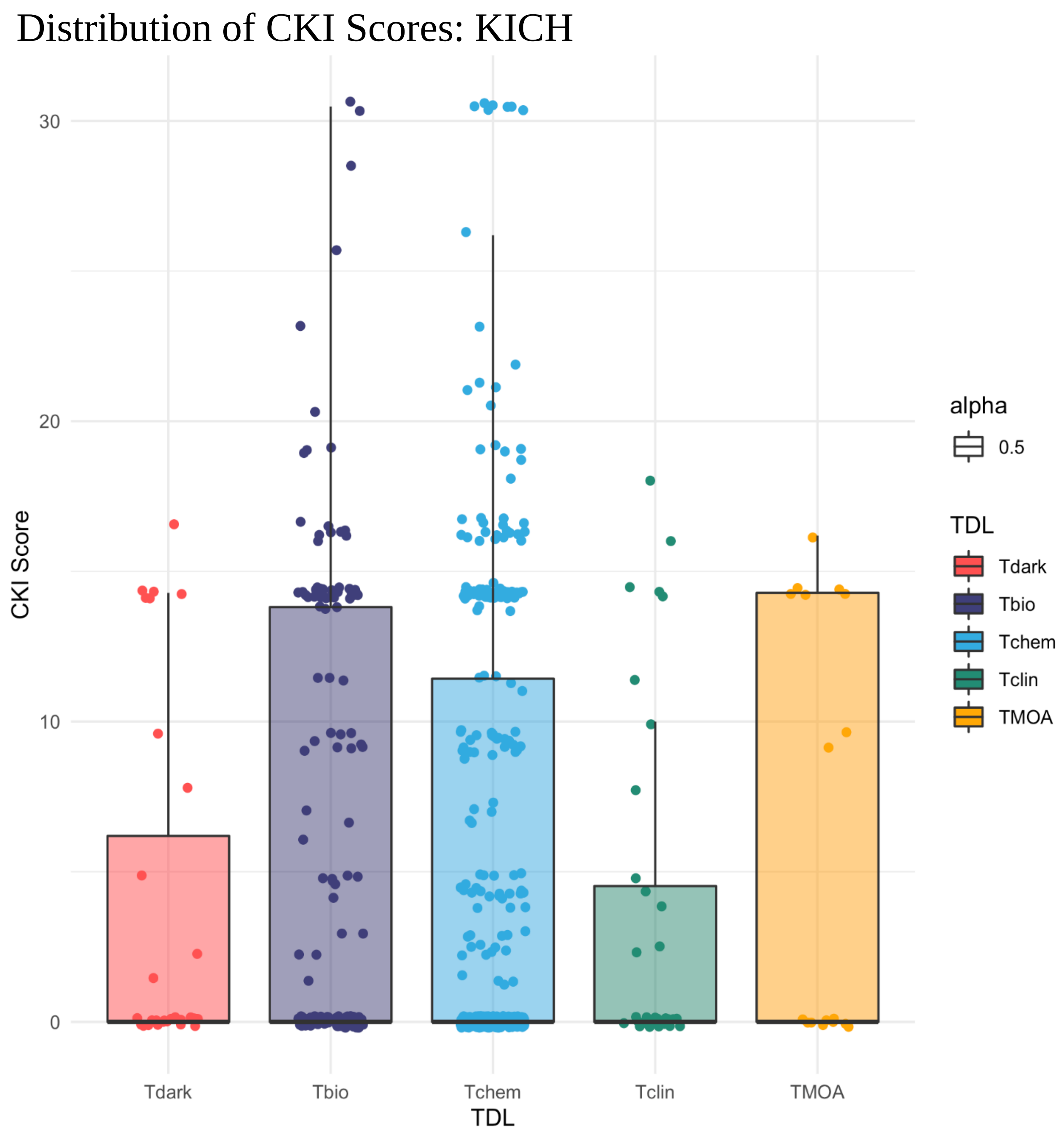
Distribution of CKI Scores: KICH
CKI Score
30
20
10
0
Tdark
Tbio
Tchem
Tclin
TMOA
TDL
alpha
0.5
TDL
Tdark
Tbio
Tchem
Tclin
TMOA

2I.

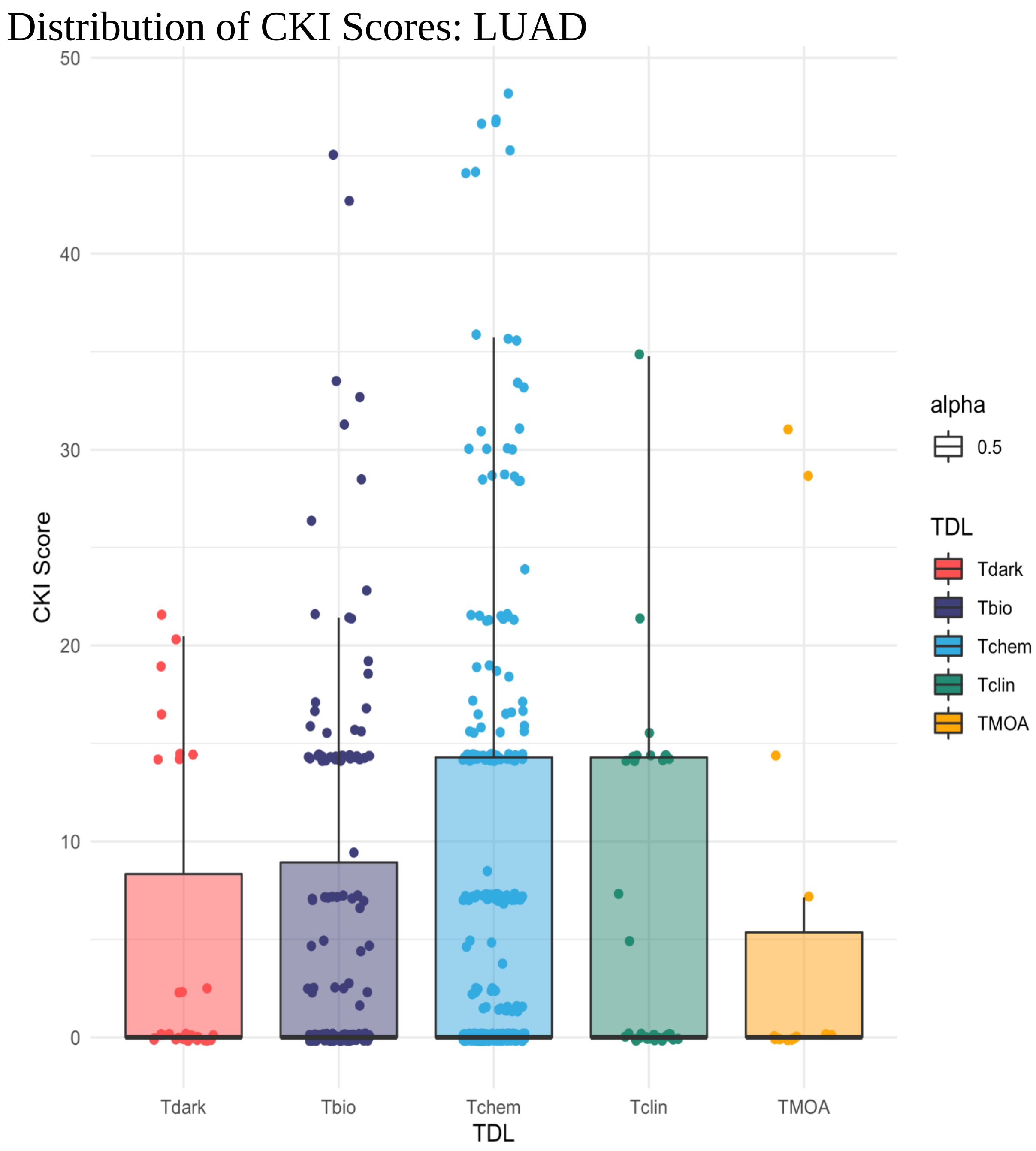

Distribution of CKI Scores: LUAD
50
40
30
20
10
0
CKI Score
Tdark
Tbio
Tchem
Tclin
TMOA
TDL
alpha
0.5
TDL
Tdark
Tbio
Tchem
Tclin
TMOA

2J.

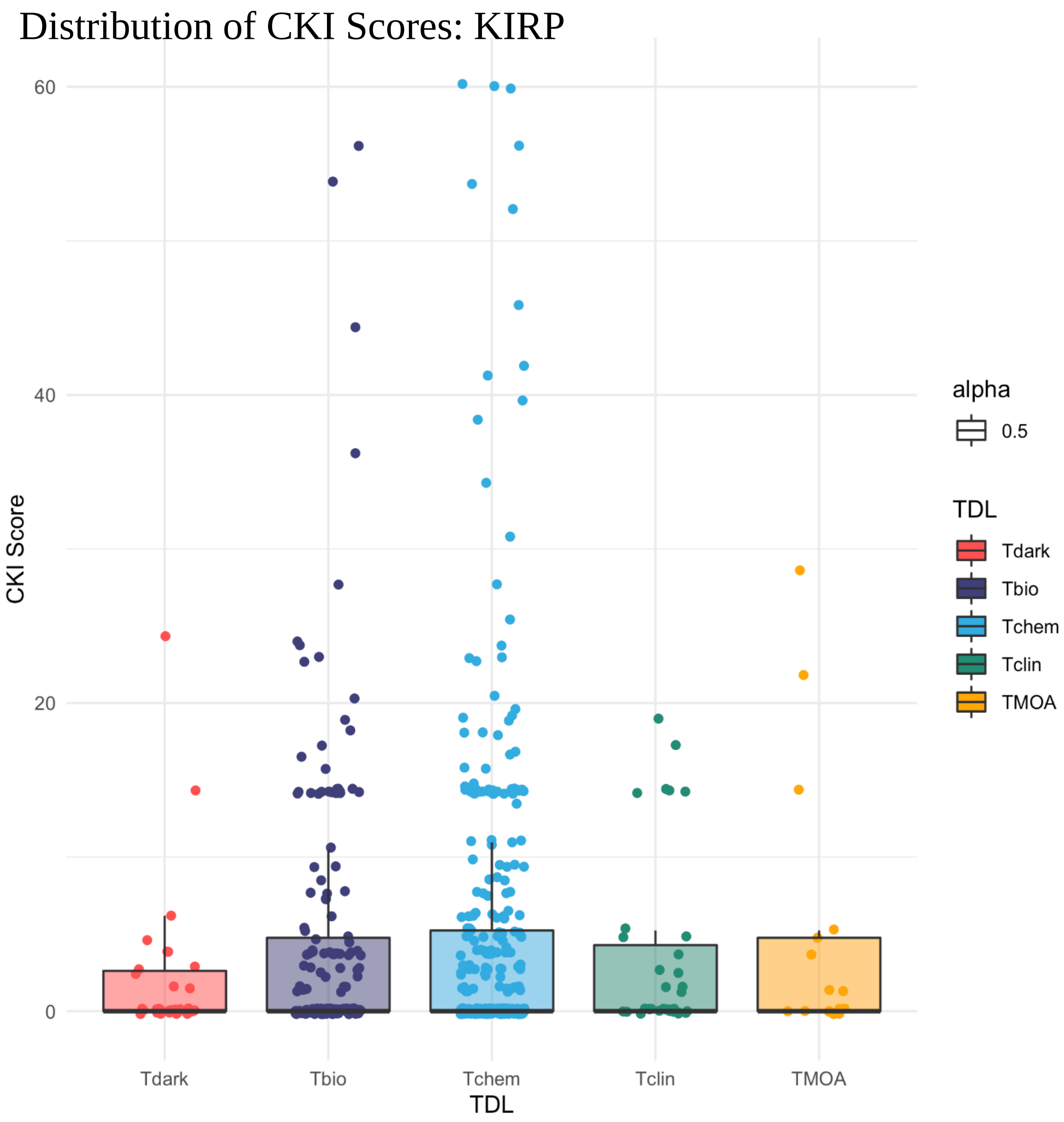
Distribution of CKI Scores: KIRP
60
40
20
0
CKI Score
Tdark
Tbio
Tchem
Tclin
TMOA
TDL
alpha
0.5
TDL
Tdark
Tbio
Tchem
Tclin
TMOA

2K.

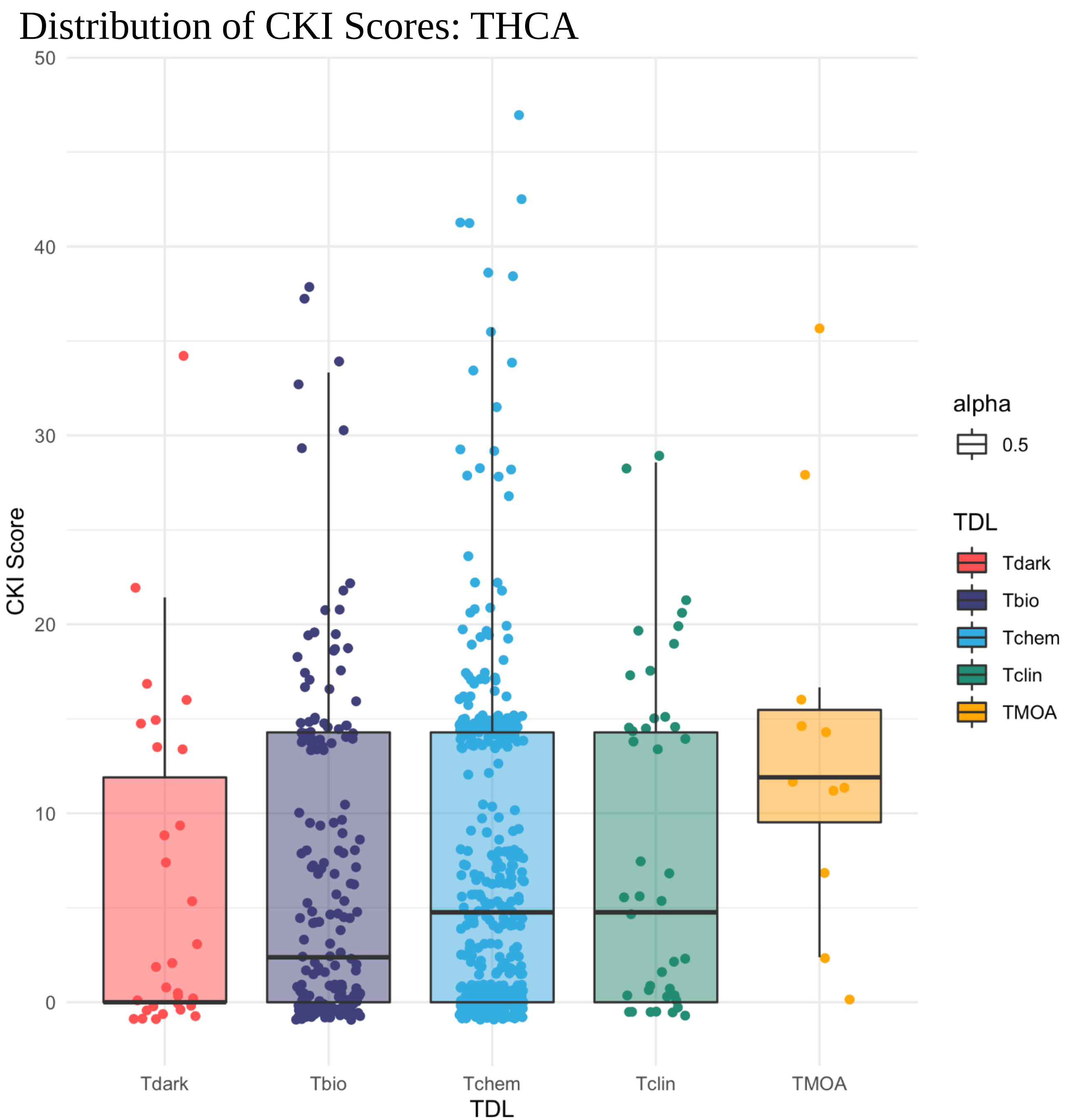
Distribution of CKI Scores: THCA
CKI Score
50
40
30
20
10
0
Tdark
Tbio
Tchem
Tclin
TMOA
TDL
alpha
0.5
TDL
Tdark
Tbio
Tchem
Tclin
TMOA

2L.

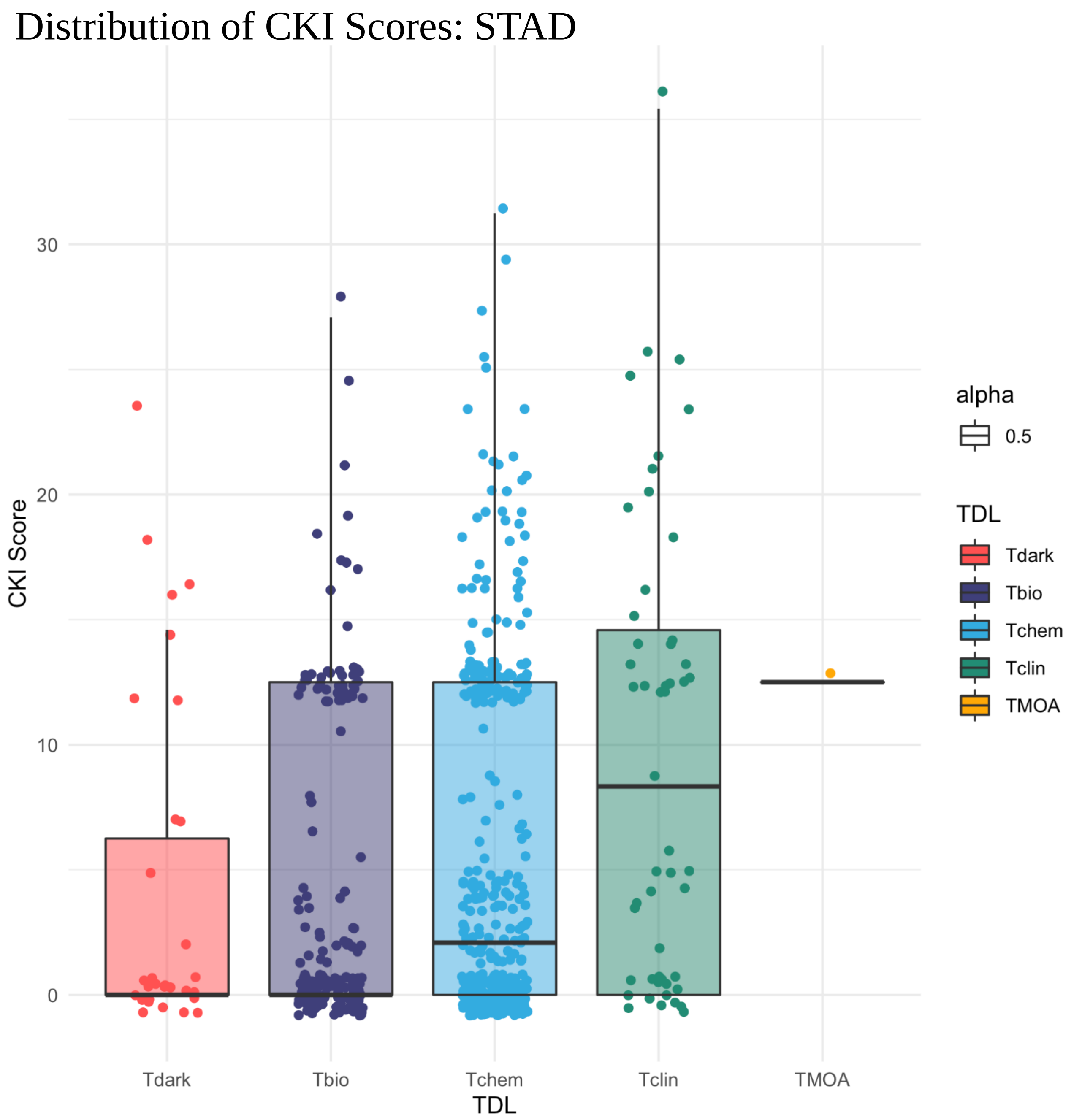

Distribution of CKI Scores: STAD
CKI Score
30
20
10
0
Tdark
Tbio
Tchem
Tclin
TMOA
TDL
alpha
0.5
TDL
Tdark
Tbio
Tchem
Tclin
TMOA

2M.

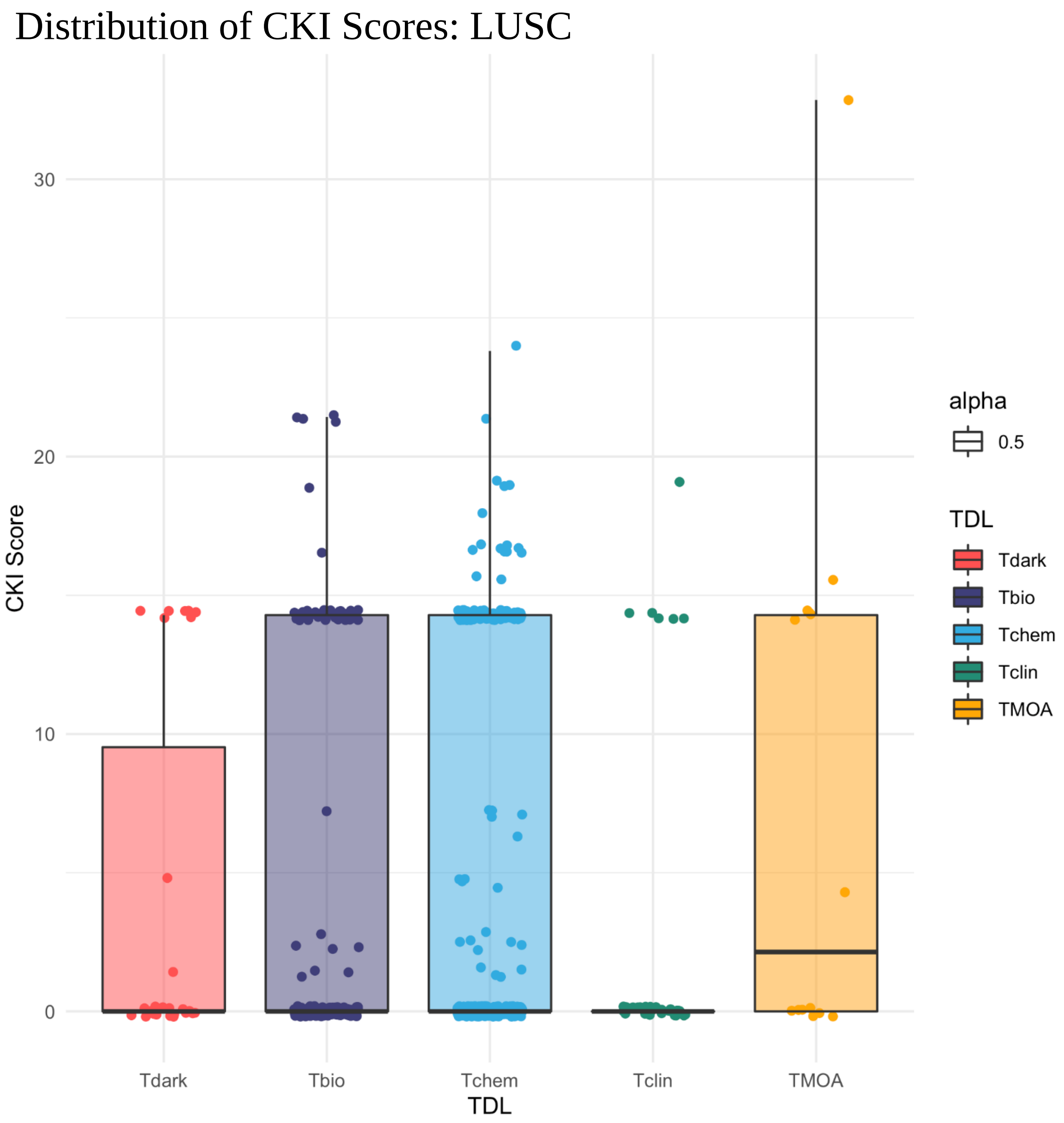

Distribution of CKI Scores: LUSC
CKI Score
30
20
10
0
Tdark
Tbio
Tchem
Tclin
TMOA
TDL
alpha
0.5
TDL
Tdark
Tbio
Tchem
Tclin
TMOA

2N.

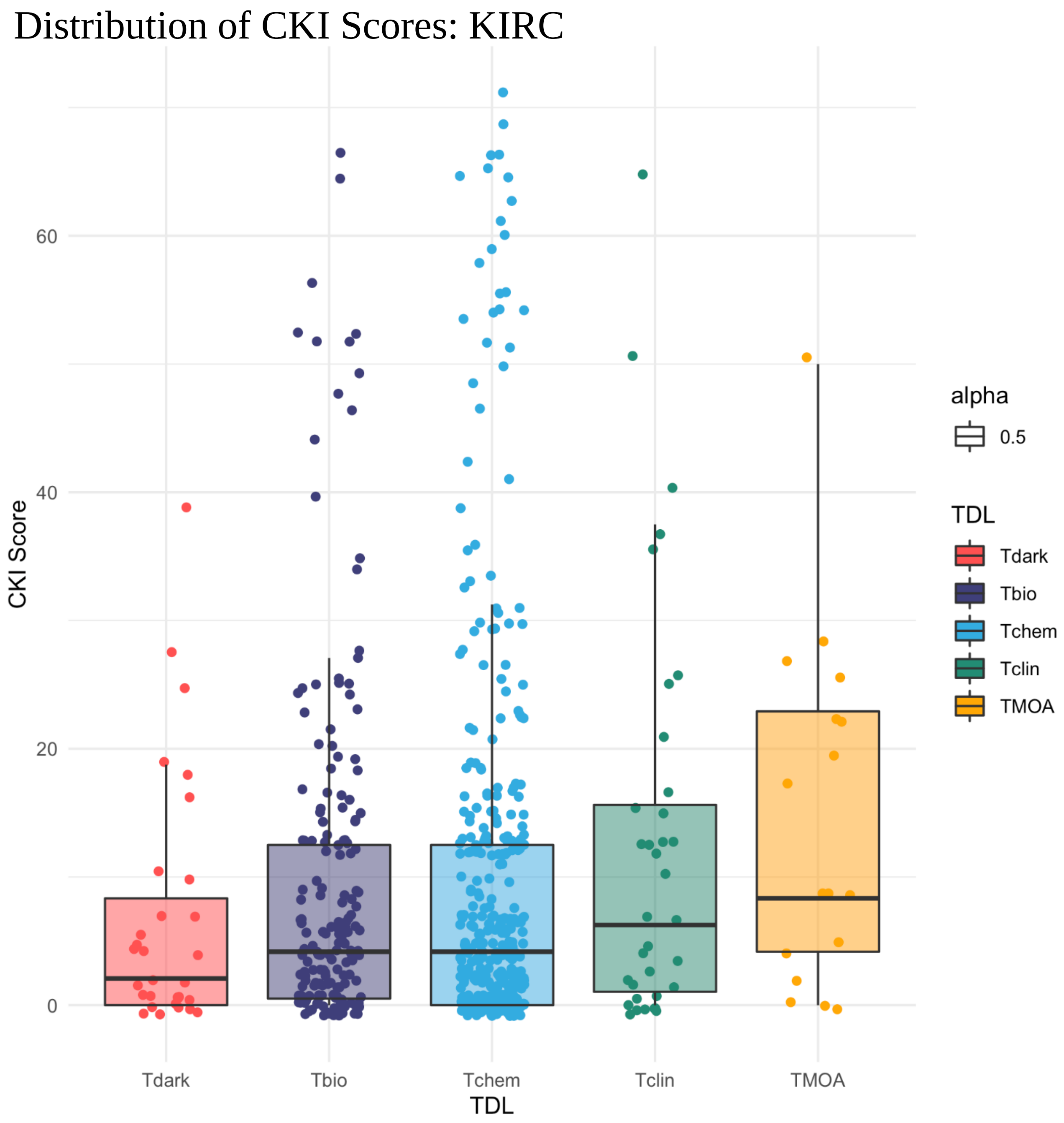
Distribution of CKI Scores: KIRC
CKI Score
60
40
20
0
Tdark
Tbio
Tchem
Tclin
TMOA
TDL
alpha
0.5
TDL
Tdark
Tbio
Tchem
Tclin
TMOA
